# Synthesis and Antimicrobial Activity of (*E*)-1-Aryl-2-(1H-tetrazol-5-yl)acrylonitrile Derivatives via [3+2] Cycloaddition Reaction Using Reusable Heterogeneous Nanocatalyst under Microwave Irradiation

**DOI:** 10.3390/molecules29184339

**Published:** 2024-09-12

**Authors:** Ayashkanta Nanda, Navneet Kaur, Manvinder Kaur, Fohad Mabood Husain, Haesook Han, Pradip K. Bhowmik, Harvinder Singh Sohal

**Affiliations:** 1 Medicinal and Natural Product Laboratory, Department of Chemistry, Chandigarh University, Gharuan, Mohali 140413, Punjab, India; 2 Department of Food Science and Nutrition, College of Food and Agriculture Sciences, King Saud University, Riyadh 11451, Saudi Arabia; 3 Department of Chemistry and Biochemistry, University of Nevada Las Vegas, 4505 S. Maryland Parkway, Box 454003, Las Vegas, NV 89154, USA

**Keywords:** magnetic nanoparticles, heterogenous catalyst, malononitrile derivatives, tetrazole, recyclable catalyst

## Abstract

The magnetically recoverable heterogeneous Fe_2_O_3_@cellulose@Mn nanocomposite was synthesized by a stepwise fabrication of Mn nanoparticles on cellulose-modified magnetic Fe_2_O_3_ nanocomposites, and the morphology of the nanocomposite was characterized through advanced spectroscopic techniques. This nanocomposite was investigated as a heterogeneous catalyst for the synthesis of medicinally important tetrazole derivatives through Knoevenagel condensation between aromatic/heteroaromatic aldehyde and malononitrile followed by [3+2] cycloaddition reaction with sodium azide. Thirteen potent (*E*)-1-aryl-2-(1H-tetrazol-5-yl)acrylonitrile derivatives are reported in this paper with very high yields (up to 98%) and with excellent purity (as crystals) in a very short period (3 min @ 120 W) using microwave irradiation. The present procedure offers several advantages over recent protocols, including minimal catalyst loading, quick reaction time, and the utilization of an eco-friendly solvent. Furthermore, the synthesized (*E*)-1-aryl-2-(1H-tetrazol-5-yl)acrylonitrile derivatives (**4b**, **4c**, and **4m**) are shown to have excellent resistance against various fungal strains over bacterial strains as compared to the standard drugs Cefixime (4 μg/mL) and Fluconazole (2 μg/mL).

## 1. Introduction

Heterocycles are crucial in various fields, including pharmaceuticals [1,2], natural resources [3], veterinary medicine [4], analytical reagents [5], agricultural products [6], and dyes [7]. Among the most widely utilized reactions for synthesizing five-membered heterocyclic compounds are [3+2] cycloaddition reactions [8,9]. [3+2] cycloaddition reactions involving three-atom components and alkene/alkyne molecular systems provide a broad spectrum of opportunities for synthesizing various types of five-membered heterocycles with one or more than one heteroatom [10,11]. Nowadays, synthesis of a variety of five- and six-member heterocyclic compounds via multicomponent reaction (MCR) is one of the most efficient techniques because it reduces human effort with the reduction in chemical waste [12]. 

Tetrazoles, nitrogen-rich heterocyclic compounds, have attracted considerable interest due to their extensive range of applications in pharmaceuticals, as well as biological science [13,14]. Additionally, tetrazoles display acidic characteristics owing to the existence of a free N–H group. This property facilitates the creation of various aliphatic and aromatic heterocyclic compounds via nucleophilic substitution reactions [15,16] as well as facilitates their binding to enzymes and receptors within organisms. These interactions occur through weak forces, including coordination bonds, hydrogen bonds, van der Waals forces, π–π stacking, hydrophobic effects, and cation–π interactions [17]. As a result, tetrazoles exhibit diverse biological activities and play a significant role in the pharmaceutical domain. Clinicians commonly employ tetrazole-based antimicrobial medications, such as Cefamandole, Ceftezole, Tedizolid [18], Flomoxef, Cefonicid [19], etc.

A variety of molecular systems are listed in the literature to catalyze the [3+2] cycloaddition reactions of azides effectively. Organocatalysts and heterogeneous catalysts were found to be best due to the enhanced efficiency, improved yields, and selectivity of these reactions [20,21] and other broader applicability in synthetic chemistry [22]. Traditionally, the synthesis of tetrazoles has involved methods such as the cycloaddition of azides with nitriles or the use of toxic reagents like sodium azide in the presence of strong acids [23]. Moreover, the regioselectivity in azide cycloaddition reactions is dictated by a combination of structural, steric, and electronic factors, making it a vital area of study in organic chemistry [24]. These conventional methods often pose environmental and safety hazards due to the usage of toxic chemicals and toxic reaction environments, along the production of hazardous side products [25,26]. Consequently, there is a pressing need for more sustainable and environmentally friendly approaches to tetrazole synthesis [27]. 

The nitrile triple bond typically exhibits inactivity in cycloaddition processes; therefore, typical or specific catalysts have been used for such transformations [25]. A unique example of a non-catalyzed [3+2] cycloaddition involving these bonds was demonstrated by P. Wolinski and co-workers, although the product yield was quite low [28]. To address this, heterogeneous catalysts have demonstrated remarkable effectiveness. Recently, employing heterogeneous catalysts has appeared as a promising approach for achieving greener chemical processes [29]. This approach has been further refined over the decades by utilizing different catalytic systems or modifying substrates [30,31]. Heterogeneous catalysts offer several advantages, including ease of separation, recyclability, and reduced environmental impact [32]. Among these, metal oxide nanoparticles have gained significant attention owing to their high surface area and catalytic efficiency [33]. Iron oxides (Fe_3_O_4_) synthesized using plant extracts nanoparticles have shown great potential as catalysts due to their magnetic properties, which facilitate easy recovery and reuse [34]. The development of greener synthesis methods for tetrazoles using heterogeneous catalysts is a critical step towards sustainable chemistry [35,36].

In our study, we introduce an approach to synthesize (*E*)-1-aryl-2-(1H-tetrazol-5-yl)acrylonitrile under microwave irradiation using an Fe_2_O_3_@cellulose@Mn nanocomposite, which is both an efficient and recyclable heterogeneous catalyst. Microwave irradiation offers the benefits of reduced reaction times and enhanced yields, making it an attractive alternative to conventional heating methods. Our results demonstrate the high catalytic activity and recyclability of Fe_2_O_3_@cellulose@Mn nanocomposites, highlighting their potential for sustainable chemical processes.

## 2. Results and Discussion

### 2.1. Characterization of Fe_2_O_3_@Cellulose@Mn Nanoparticle

#### 2.1.1. FT-IR Characterization

**Figure 1:**
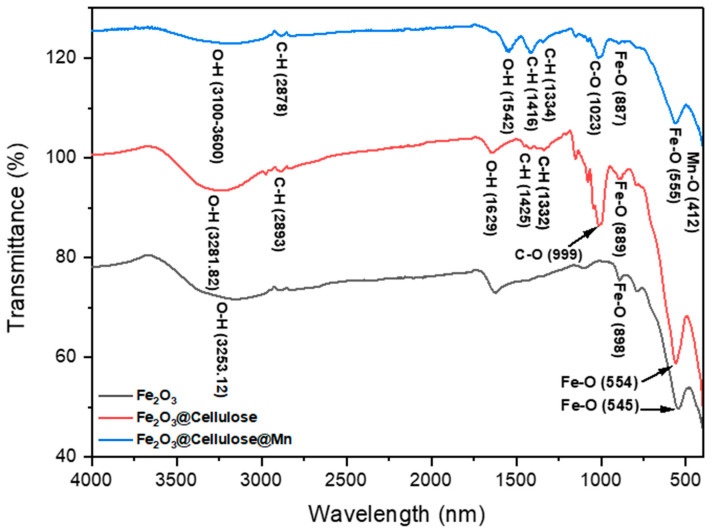
FT-IR characterization of Fe_2_O_3_, Fe_2_O_3_@cellulose, and Fe_2_O_3_@cellulose@Mn nanocomposites.

The FT-IR spectra of Fe_2_O_3_, Fe_2_O_3_@cellulose, and Fe_2_O_3_@cellulose@Mn nanoparticles show distinct differences due to the incorporation of cellulose and Mn, as shown in Figure 1. Fe_2_O_3_ nanoparticles exhibit strong Fe-O stretching vibrations at 898 cm^−1^ and 545 cm^−1^, along with peaks for O-H stretching vibrations at 3253.12 cm^−1^. In contrast, Fe₂O₃@cellulose nanoparticles display additional peaks characteristic of cellulose, such as broad O-H stretching vibrations around 3340–3400 cm^−1^, C-H stretching at 2893, 1425, and 1332 cm^−1^, the band at 1629 cm^−1^ is attributed to O-H bending arising from absorbed water, and C-O stretching vibrations at 999 cm^−1^, with Fe-O stretching vibrations at 889 cm^−1^ and 554 cm^−1^ [37]. The Fe₂O₃@cellulose@Mn nanoparticles show similar cellulose peaks, such as broad O-H stretching vibrations around 3100–3600 cm^−1^, C-H stretching at 2878, 1416, and 1334 cm^−1^, the band at 1542 cm^−1^ is attributed to O-H bending arising from absorbed water, and C-O stretching vibrations at 1023 cm^−1^, with Fe-O stretching vibrations at 887 cm^−1^ and 555 cm^−1^ and additional Mn-O peaks in the range of 412 cm^−1^, indicating successful incorporation of Mn [38]. These differences reflect the interaction between Fe₂O₃, cellulose, and Mn, resulting in a more complex and less crystalline structure in the composite nanoparticles.

#### 2.1.2. SEM Characterization

**Figure 2:**
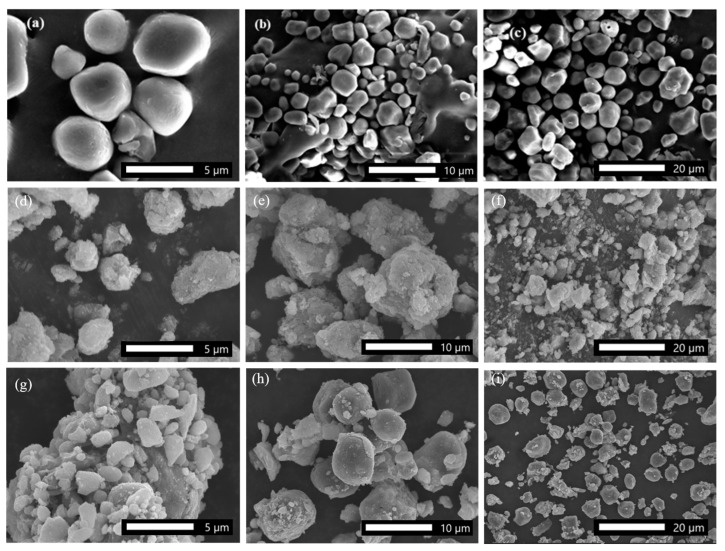
SEM images of (**a**–**c**) cellulose, (**d**–**f**) Fe_2_O_3_, and (**g**–**i**) Fe_2_O_3_@cellulose@Mn.

SEM characterization of cellulose, Fe_2_O_3_, and Fe_2_O_3_@cellulose@Mn offers valuable insights into the surface morphology and structural features, as shown in Figure 2a–i. The surface roughness observed via SEM significantly influences catalysis. Rough surfaces tend to enhance the number of active sites available for reactions. However, alongside this benefit, nanoparticles can also agglomerate, which may impact material properties, altering surface area and reactivity. In Figure 2a–c, SEM illustrates the spherical, smooth, and uniform surface before fabrication [39]. For Fe_2_O_3_, in Figure 2d–f, SEM typically reveals its nanoparticle or microsphere form with distinct surface roughness and low particle agglomeration, reflecting its high surface area and catalytic potential. When Fe₂O₃ is coated with cellulose and manganese (Fe_2_O_3_@cellulose@Mn), as shown in Figure 2g–i, SEM can illustrate the uniform distribution of the cellulose layer and the presence of manganese particles or clusters on the surface. This multilayered composite structure often shows a more complex morphology with improved stability and enhanced properties compared to the bare Fe_2_O_3_ [37]. 

**Figure 3:**
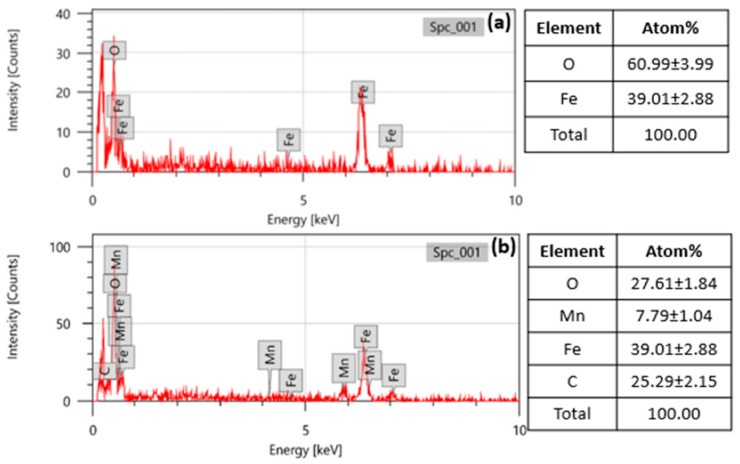
EDS characterization of (**a**) Fe_2_O_3_ and (**b**) Fe_2_O_3_@cellulose@Mn.

Through elemental investigation conducted on the microscope sector of Fe_2_O_3_ and Fe_2_O_3_@cellulose@Mn using EDS, the atomic percentages of O and Fe were determined to be 60.99 ± 3.99% and 39.01 ± 2.88%, respectively, for Fe_2_O_3_, while the atomic percentages of C, O, Mn, and Fe were determined to be 25.29 ± 2.15%, 27.61 ± 1.84%, 7.79 ± 1.04%, and 39.01 ± 2.88%, respectively, for Fe_2_O_3_@cellulose@Mn nanocomposite. These results confirm the composite nature of Fe_2_O_3_@cellulose@Mn as depicted in Figure 3.

#### 2.1.3. XRD Characterization

**Figure 4:**
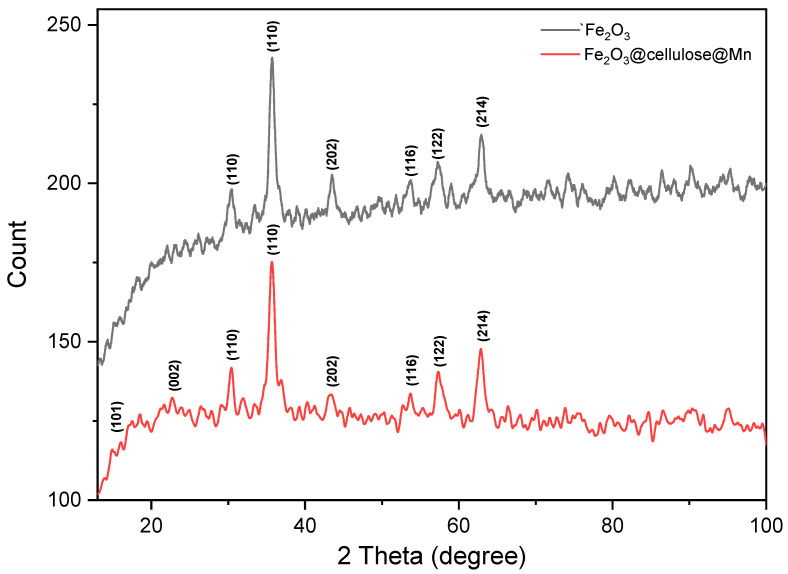
XRD characterization of Fe_2_O_3_ and Fe_2_O_3_@cellulose@Mn nanocomposites.

Figure 4 displays the XRD patterns of the Fe_2_O_3_ and Fe_2_O_3_@cellulose@Mn nanocomposites within the range of 2θ = 0–100°. The XRD peak at 40 KV was utilized to identify crystalline phases and estimate crystalline sizes. The XRD spectra of Fe_2_O_3_ and Fe_2_O_3_@cellulose@Mn nanoparticles (Figure 4) exhibit distinct differences due to the incorporation of cellulose and Mn. The Fe_2_O_3_ nanoparticles display characteristic sharp peaks at 2θ values around 30.46, 35.72, 43.47, 53.77, 57.25, and 62.94, corresponding to the (110), (110), (202), (116), (122), and (214) planes [37], respectively, indicating high crystallinity. These findings are consistent with previous studies on Fe_2_O_3_ nanoparticles.

In contrast, the Fe_2_O_3_@cellulose@Mn nanoparticles show additional peaks due to the presence of cellulose and Mn, with cellulose peaks appearing around 2θ values of 15° and 22.5°, corresponding to the (101) and (002) [38], respectively, which is consistent with the literature on cellulose nanomaterials. The crystallinity of the composite nanoparticles is generally lower, as evidenced by broader and less intense peaks, reflecting a more disordered structure. This reduction in crystallinity is attributed to the amorphous nature of cellulose and the interaction between Fe_2_O_3_ and Mn, which disrupts the ordered crystal structure of Fe_2_O_3_.

#### 2.1.4. VSM Characterization

**Figure 5:**
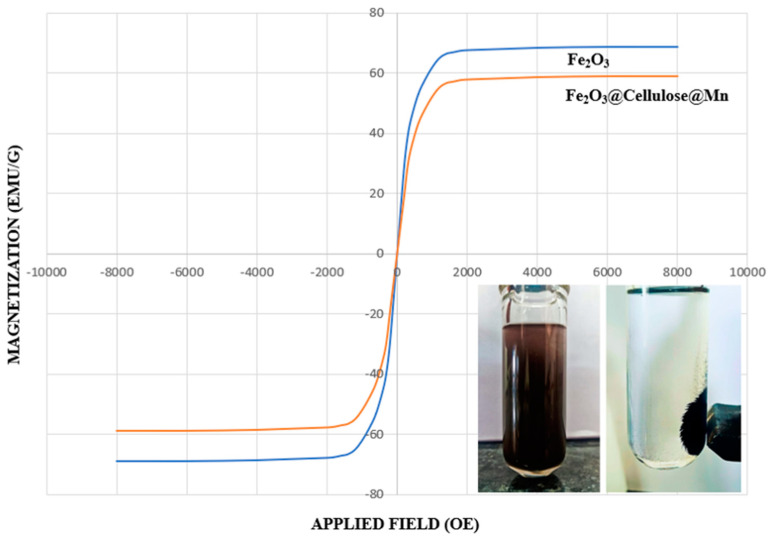
VSM characterization of Fe_2_O_3_ and Fe_2_O_3_@cellulose@Mn nanocomposites.

The synthesized nanocomposite displays magnetic characteristics, allowing for its separation using a magnet. VSM was employed to analyze the magnetic behavior of the Fe_2_O_3_@cellulose@Mn nanocomposite. The hysteresis loop, plotted within a range of 10,000 to −10,000 Oersted fields (Figure 5), reveals that the magnetization curve of the particles originates from the origin. Notably, no observable coercive field or residual magnetization indicates the nanoparticles possess superparamagnetic properties. The saturation magnetization value, measured at 58.8/68.8 emu/g, further supports this characterization.

### 2.2. One-Pot Synthesis of (Z)-3-Phenyl-2-(1H-tetrazol-5-yl)acrylonitrile (***4a***) Using Fe_2_O_3_@cellulose@Mn Nanocatalyst

In our preliminary experiments, we observed the presence of Fe₂O₃ catalyst produced (*Z*)-3-phenyl-2-(1H-tetrazol-5-yl)acrylonitrile (**4a**) in low yields (Table 1, entry 1), while in the presence of cellulose alone there is no change in the yield of the reaction (Table 1, entry 2). The synergistic effect of Fe_2_O_3_ and cellulose in our nanocatalyst formulation significantly enhances the reactivity of the reactants, leading to the desired product formation. Additionally, we incorporated Mn into the catalyst to further enhance its reactivity. This modification with Mn significantly improved the catalytic performance, demonstrating the importance of each component in the overall effectiveness of the nanocatalyst.

To optimize the conditions, we established a model reaction, in which a mixture of aldehyde **1** (1 mmol), malononitrile **2** (1 mmol), and sodium azide **3** (1.1 mmol) was added successively in ethanol (5 mL) containing Fe_2_O_3_@cellulose@Mn nanocatalyst at 100 watts in a monowave reactor for 10 min to synthesize desired product **4a**. We confirmed the reaction progress using TLC (hexane/ethyl acetate (8:2)) and validated the product by comparing its melting point to literature data.

Initially, the influence of catalyst loading for Fe_2_O_3_@cellulose@Mn nanocatalyst at different levels of catalytic loading was tested, and it was observed that increasing the amount of catalyst increased the yield, as shown in Table 1. The optimal amount of Fe_2_O_3_@cellulose@Mn nanocatalyst was found to be 15 mg (Table 1, entry 5). Furthermore, the increase in the amount of Fe_2_O_3_@cellulose@Mn nanocatalyst exhibits minimal impact on percentage yield.

For screening the effect of solvent, several solvents, including methanol, glycerol, water, ethanol, ethylene glycol, and PEG, were tested on a model reaction in the presence of 15 mg of Fe_2_O_3_@cellulose@Mn nanocatalyst. Among all these solvents, ethanol emerged as the most effective solvent, as demonstrated in Table 2, entry 4, yielding an excellent product.

Finally, the optimization of power was examined for the condensation reaction between aldehyde **1** (1 mmol), malononitrile **2** (1 mmol), and sodium azide **3** (1.1 mmol) conducted in the presence of 15 mg of Fe_2_O_3_@cellulose@Mn nanocatalyst in ethanol (5 mL), as depicted in Table 3. It was observed that with an increase in power, the product yield showed a notable increase over time. Conversely, elevating the power beyond 120 watts led to a decline in product yield, attributed to the decomposition of products at higher temperatures. As a result of this, the optimal power for the modified model reaction is 120 watts (Table 3, entry 4).

After the optimization of reaction conditions, it is important to investigate the recyclability of the Fe_2_O_3_@cellulose@Mn nanocatalyst (Table 4). After the separation from the previous reaction, the Fe_2_O_3_@SiO_2_ nanocatalyst was washed with dry acetone and vacuum-dried before being reused for the synthesis of compound **4a**. Impressively, product **4a** was obtained with a remarkable yield of 98%. The catalyst maintained its efficiency over four runs, showing no significant decrease in yield, as shown in Table 4, which confirms its continued utility.

The synthesis of (*E*)-1-aryl-2-(1H-tetrazol-5-yl)acrylonitrile **4a**–**m** in a one-pot reaction involved the sequential addition of sodium azide **3** (1.1 mmol) to previously synthesized 2-benzylidene malononitrile derivatives **4a**–**m** under the optimized conditions. Furthermore, a screening of reaction time was conducted to optimize this step. By varying the duration of the reaction time, the ideal timeframe for the conversion of the 2-benzylidene malononitrile derivatives to the desired (*E*)-1-aryl-2-(1H-tetrazol-5-yl)acrylonitrile was identified. At different time intervals, the progress of the reaction was monitored, using TLC with a solvent system of hexane/ethyl acetate (8:2), to determine the optimal conditions for maximum yield and efficiency. From Table 5, it is evident that as the reaction time increases from 1 to 3 min, there is a notable enhancement in the yield percentage, with a significant jump from 75% at 1 min to 98% at 3 min. This suggests that the synthesis reaction reaches an optimal state at around 3 min, resulting in the highest yield achieved in the experiment (Table 5, entry 3). Beyond the optimal condition, prolonged exposure to the reaction conditions causes the product to start decomposing, which results in a reduction in the overall yield of the desired product.

The reaction yielded excellent results with various aromatic or heterocyclic aldehydes **1a**–**m**, as depicted in Table 6. Notably, high to excellent yields of the desired products **4a**–**m** were achieved within short reaction durations. Aromatic aldehydes with carbocyclic rings possessing electron-withdrawing groups **1a**–**g** were tested (entries **1**–**7**, Table 4), alongside aromatic aldehydes with carbocyclic rings containing electron-donating groups **1h**–**l** and electron-rich heterocycle **1m**. These substrates successfully engaged under optimized conditions to produce the corresponding (*E*)-1-aryl-2-(1H-tetrazol-5-yl)acrylonitrile derivatives **4h**–**m** (entries **8**–**13**, Table 4).

### 2.3. Characterization of (Z)-3-Phenyl-2-(1H-tetrazol-5-yl)acrylonitrile ***4a*** Using Fe_2_O_3_@cellulose@Mn Nanocomposite

The structure of the compound is identified through the utilization of different spectroscopic methodologies such as FT-IR, ^1^H NMR, and ^13^C NMR. In the FT-IR spectrum of compound **4a**, stretching peaks at 3032.93 cm^−1^ have been observed for sp^2^ hybridized C-H bond, and peaks at 2222.61 cm^−1^ belong to C≡N stretching. The ^1^H NMR spectra (500 MHz, CDCl_3_) of compound **4a** indicate the formation of a final product with high purity, singles at δ 7.78 ppm and 3.14 ppm were observed for C-H and N-H, respectively, and singles from δ 7.52 to 7.91 have been observed for the aromatic protons [8]. In ^13^C NMR (125 MHz, CDCl_3_), the most down-shielded peak at δ 159.05 ppm and 81.70 ppm was observed for the C-1 and C-2 carbon. Peaks at δ 133.62 (C-1′), 129.91 (C-2′ and C-6′), 129.71 (C-3′ and C-5′), and 128.61 (C-4′) ppm were observed for the aromatic region. The peaks of one C≡N group and C-3 were observed at 112.74 and 111.59, respectively [8]. Further, the melting point of the synthesized compound **4a** (165–167 °C) was compared with its melting point in the literature (168–170 °C), also indicating the formation of the final product **4a**.

### 2.4. Plausible Mechanism

**Scheme 1:**
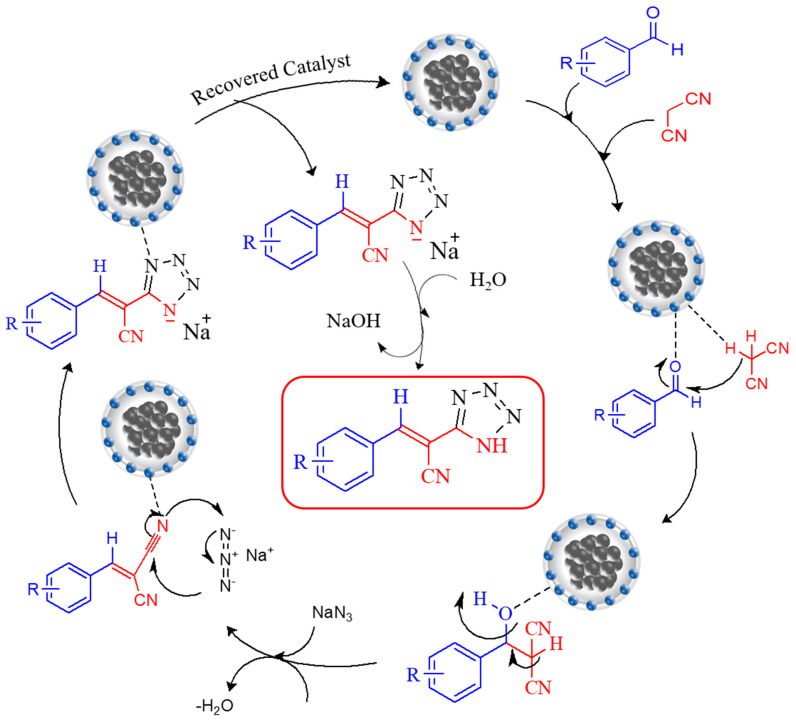
Proposed mechanism for the synthesis of (*E*)-1-aryl-2-(1H-tetrazol-5-yl)acrylonitrile derivatives **4a**–**m** using Fe_2_O_3_@cellulose@Mn nanocatalyst.

Scheme 1 illustrates the cascade one-pot synthesis pathway for the production of (*E*)-1-aryl-2-(1H-tetrazol-5-yl)acrylonitrile derivatives **4a**–**m** utilizing the magnetic Fe_2_O_3_@cellulose@Mn nanocomposites. In the initial step, aromatic aldehydes **1a**–**m** are incrementally activated by magnetic Fe_2_O_3_@cellulose@Mn nanocomposites, leading to their condensation with the C–H of malononitrile **2**, resulting in the formation of a Knoevenagel aryl/heteroarylidene malononitrile intermediate. Subsequently, this intermediate participates in a concerted [3+2] cycloaddition reaction with sodium azide **3**. This cycloaddition event results in the formation of a five-membered tetrazole ring, ultimately yielding the desired product [44].

### 2.5. Antimicrobial Activity

The antimicrobial activities of the synthesized (*E*)-1-aryl-2-(1H-tetrazol-5-yl)acrylonitrile derivatives **4a**–**m** were examined by the minimum inhibitory concentration (MIC) method. Cefixime (4 μg/mL) and Fluconazole (2 μg/mL) were taken as standard drugs to compare the effectiveness of the prepared (*E*)-1-aryl-2-(1H-tetrazol-5-yl)acrylonitrile derivatives **4a**–**m** against the mentioned bacterial and fungal strains. From Table 7, it is evident that all the (*E*)-1-aryl-2-(1H-tetrazol-5-yl)acrylonitrile derivatives **4a**–**m** exhibit excellent interaction with the mentioned microbes as compared to the standard drugs. Interestingly, all the 1H-tetrazole derivatives show excellent resistance against the fungal strains (*A. janus*, *S. aureus*, and *A. niger*) as compared to the bacterial strains. Further, (*E*)-1-aryl-2-(1H-tetrazol-5-yl)acrylonitrile derivatives **4a**–**m** which have polar groups attached to the phenyl ring (**4b**, **4c**, and **4m**) were found to be the most efficient drugs against all the tested strains with almost equal MIC values because they tend to form H-binding with the microbe’s protein. Compounds containing non-polar groups (**4h**, **4i**, and **4j**) were also efficient but at higher MIC values (16–64 μg/mL).

### 2.6. Comparison of the Catalytical Activity of Fe_2_O_3_@cellulose@Mn Nanocatalyst with Other Heterogeneous Catalysts in the Synthesis of Tetrazole Derivatives

Table 8 compares previously reported methods for synthesizing tetrazole **4a** and the current protocol. The advantages of our method are evident, including high to excellent yields, the avoidance of toxic or carcinogenic solvents like DMF, shorter reaction times, and the easy separation of the catalyst from the reaction mixture. These benefits make our protocol superior to most of the previously reported methods.

## 3. Materials and Methods

### 3.1. Materials and Instruments

The chemicals utilized in this study were sourced from Sigma Aldrich (Uttar Pradesh, India) and were used without further purification, whereas solvents were procured from Changshu Song Sheng Fine Chemical (Mumbai, India). The melting points of all resulting products were measured using a digital melting point apparatus employing the open capillary method. FT-IR spectra of the targeted compound were obtained using the ATR mode on a Perkin Elmer Spectrum II instrument (Chandigarh University, Gharuan, Punjab, India). ^1^H NMR and ^13^C NMR spectra were recorded on a Bruker Avance NEO 500 MHz NMR spectrometer (Punjab University, Chandigarh, Punjab, India), utilizing CDCl_3_ as the solvent. The thin-layer chromatography (TLC) technique was employed to monitor reaction progress and assess compound purity, with TLC visualized using a UV chamber. SEM micrographs were acquired using a JSM IT500 scanning electron microscope (Chandigarh University, Gharuan, Punjab, India). Elemental analysis of microscopic sections of the Fe_2_O_3_@Cellulose@Mn sample was performed via EDS. SEM images were captured under high vacuum mode, ranging from 30 nm (30 kV) to 15.0 nm (1.0 kV). XRD patterns of dried (lyophilized) samples were obtained at room temperature using a Bruker D8 advanced instrument (Chandigarh University, Gharuan, Punjab, India). The magnetic properties of compounds were investigated using a Lake Shore 7410 Series vibrating sample magnetometer (Punjab Agricultural University, Ludhiana, Punjab, India).

### 3.2. Synthesis of Fe_2_O_3_ MNPs

**Scheme 2:**
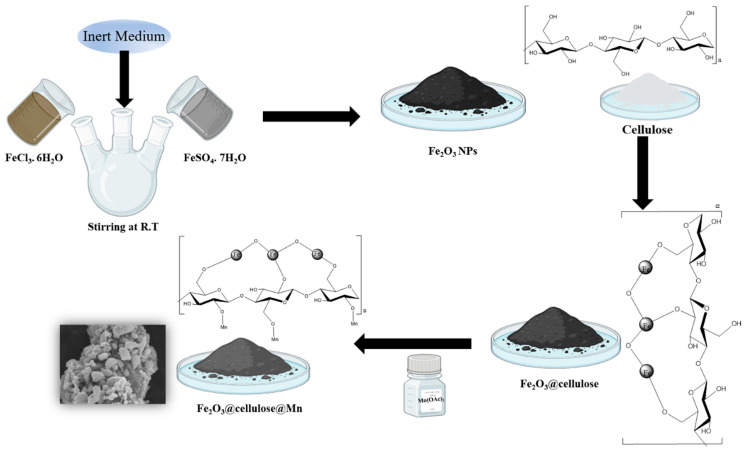
Graphical illustration of the synthesis of Fe_2_O_3_@cellulose@Mn nanocatalyst.

The synthesis of iron magnetic nanoparticles (MNPs) followed the co-precipitation method outlined in Scheme 2. Fe_2_O_3_ MNPs were prepared by combining 12.0 g of FeCl_3_ (0.4 M) and 6.0 g of FeSO_4_·7H_2_O (0.2 M) dissolved in 100 mL of deionized (DI) water. The mixture was purged with N_2_ gas and stirred for approximately one hour. Subsequently, liquid ammonia (25%) was added dropwise to the flask. The pH of the solution was further adjusted to ~10 by the addition of 2.0 M NaOH solution. The solution temperature was then raised to 70 °C with continuous stirring and N_2_ gas purging for 5 h, resulting in the formation of a black precipitate in the flask. The precipitate was filtered, washed with acetone, and then rinsed with DI water until reaching a neutral pH. Finally, the precipitate was dried at 60 °C in a vacuum oven, and the solid Fe_2_O_3_ MNPs were isolated by filtration.

### 3.3. Synthesis of Fe_2_O_3_@Cellulose

The preparation of Fe_2_O_3_@Cellulose was conducted following the procedure (Scheme 2). Fe_2_O_3_ MNPs (1.5 g) were placed in 250 mL of ethanol/water (1:1) with sonication for 30 min, and then 2 g of cellulose was added. Under the N_2_ atmosphere, the reaction mixture was stirred at 40 °C for 6 h. The nanoparticle product (Fe_2_O_3_@Cellulose) was dried at room temperature. This solid was filtered to isolate the solid Fe_2_O_3_@Cellulose.

### 3.4. Synthesis of Fe_2_O_3_@Cellulose@Mn

Fe_2_O_3_@Cellulose (2.5 g) in absolute ethanol (30 mL) was mixed with Mn(OAc)_2_ (5 mmol) and stirred under reflux for 8 h. Magnetic decantation was then used to separate the synthesized nanosolid (Fe_2_O_3_@cellulose@Mn) (Scheme 2). Several times, absolute ethanol was used to wash and dry the nanomagnetic catalyst at room temperature under a vacuum.

### 3.5. Synthesis of (E)-1-Aryl-2-(1H-tetrazol-5-yl)acrylonitrile Derivatives (***4a**–**m***)

**Scheme 3:**
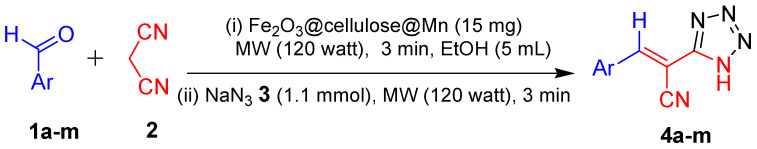
Synthesis of (*E*)-1-aryl-2-(1H-tetrazol-5-yl)acrylonitrile derivatives **4a**–**m** using Fe_2_O_3_@cellulose@Mn under MW irradiations.

A mixture containing aldehyde **1a**–**m** (1 mmol) and malononitrile **2** (1 mmol) in the presence of Fe_2_O_3_@cellulose@Mn magnetic nanocatalyst was exposed to microwave irradiation at 120 watts for 3 min in 5 mL ethanol (Scheme 3). The reaction progress was monitored via TLC using ethyl acetate/*n*-hexane (1:3) while the product confirmation was achieved by comparing its melting point to the literature values. The consistency with the literature values indicates successful synthesis.

After the reaction completion, (*E*)-1-aryl-2-(1H-tetrazol-5-yl)acrylonitrile derivatives **4a**–**m** were synthesized by sequentially adding sodium azide **3** (1.1 mmol) to the reaction mixture and then exposing them to MW irradiations at 120 watts for 3 min. The progression of the reaction was monitored using TLC (*n*-hexane/ethyl acetate/8:2); then, to isolate the crude product, distilled water was added to the reaction mixture, and then it was recrystallized using ethanol, dried, and the desired product was recovered with an efficiency of 97–98%.

*(Z)-3-phenyl-2-(1H-tetrazol-5-yl)acrylonitrile* (**4a**): Yield 98%; pale yellow crystals; mp: 165–167 °C, Lit. mp: 168–170 °C; FT-IR (ν, cm^−1^): 3252 (NH), 3032.93 (C-H), 2222.61 (C≡N). ^1^H NMR spectrum (δ, ppm, CDCl_3_, 500 MHz): 7.91–7.89 (d, 1H, Ar-H), 7.78 (s, 1H, CH), 7.65–7.61 (t, 2H, Ar-H), 7.55–7.52 (t, 2H, Ar-H), 3.1 (*br.s*, 1H, NH, exchangeable). ^13^C NMR spectrum (δ, ppm, CDCl_3_, 125 MHz): 159.05 (C-1), 133.62 (C-1′), 129.91 (C-2′ and C-6′), 129.71 (C-3′ and C-5′), 128.61 (C-4′), 112.74 (C≡N), 111.59 (C-3), 81.70 (C-2). Anal. calcd. for C_10_H_7_N_5_: C, 60.91%; H, 3.58%; N, 35.51%. Found: C, 60.89%; H, 3.55%; N, 35.56%; ESI-MS (*m*/*z*); M + 1 = 198.07.

### 3.6. Antimicrobial Studies

The synthesized (*E*)-1-aryl-2-(1H-tetrazol-5-yl)acrylonitrile derivatives **4a**–**m** were tested against three fungal strains (*Aspergillus janus* MTCC 2751, *Aspergillus niger* MTCC 281, and *Aspergillus sclerotiorum* MTCC 1008), three Gram-negative bacteria (*Staphylococcus aureus* MTCC 96, *Klebsiella pneumonia* MTCC 3384, and *Escherichia coli* MTCC 443), and two Gram-positive bacteria (*Bacillus subtilis* MTCC 441 and *Streptococcus pyogenes* MTCC 442). Fungal strains were cultured in malt extract medium at 28 °C for 72 h, while bacterial samples were grown in nutrient broth at 37 °C for 24 h. We conducted triplicate tests on each synthesized compound, dissolving it in DMSO at varying concentrations (2, 4, 8, 16, 32, 64, and 128 g/mL) using a serial dilution method.

## 4. Conclusions

In summary, the magnetically recoverable Fe_2_O_3_@cellulose@Mn nanocatalyst serves as an efficient heterogeneous catalyst for synthesizing medicinally important (*E*)-1-aryl-2-(1H-tetrazol-5-yl)acrylonitrile derivatives. The procedure involves sequential [3+2] cycloaddition reactions and cascade condensation of sodium azide with 2-benzyldiene malononitrile derivatives. Remarkably, this procedure offers rapid reaction times, high yields, and eco-friendly conditions. By enabling rapid synthesis of (*E*)-1-aryl-2-(1H-tetrazol-5-yl)acrylonitrile derivatives with high yields and excellent purity using microwave irradiations, this procedure offers advantages over existing protocols. Notably, the reusability of nanocatalysts enhances their practical applicability. Further, the synthesized (*E*)-1-aryl-2-(1H-tetrazol-5-yl)acrylonitrile derivatives with polar groups on the phenyl ring (**5b**, **5c**, and **5m**) exhibited excellent resistance against the mentioned fungal strains over the bacterial strains as compared to the standard drugs Fluconazole (**2** μg/mL).

## Figures and Tables

**Table 1:** Impact of the amount of catalyst on the model reaction.

Entry	(mg)	Yield * (%)
1	Fe_2_O_3_ (5 mg)	low
2	Cellulose (5 mg)	-
3	Fe_2_O_3_@cellulose@Mn (5 mg)	71
4	Fe_2_O_3_@cellulose@Mn (10 mg)	83
5	Fe_2_O_3_@cellulose@Mn (15 mg)	92
6	Fe_2_O_3_@cellulose@Mn (20 mg)	92
7	Fe_2_O_3_@cellulose@Mn (25 mg)	93

* Yield refers to the cumulative production output of all crops. Reaction conditions: benzaldehyde (1 mmol), malononitrile (1 mmol), sodium azide (1.1 mmol), ethanol (5 mL), 100 watts.

**Table 2:** Influence of solvents on the synthesis of compound **4a**.

Entry	Solvent	Yield * (%)
1	Methanol	88
2	Glycerol	80
3	Water	-
4	Ethanol	92
5	Ethylene glycol	85
6	PEG	78

* Yield refers to the cumulative production output of all crops. Reaction conditions: benzaldehyde (1 mmol), malononitrile (1 mmol), sodium azide (1.1 mmol), Fe_2_O_3_@cellulose@Mn nanocatalyst (15 mg), 100 watts.

**Table 3:** Impact of power on the percentage yield of compound **4a**.

Entry	Watt	Time (min)	Yield * (%)
1	Reflux	600	66
2	100	10	92
3	110	7	93
4	120	3	98
5	130	3	96
6	140	3	92

* Yield refers to the cumulative production output of all crops. Reaction conditions: benzaldehyde (1 mmol), malononitrile (1 mmol), sodium azide (1.1 mmol), ethanol (5 mL), Fe_2_O_3_@cellulose@Mn nanocatalyst (15 mg).

**Table 4:** Reusability of Fe_2_O_3_@cellulose@Mn nanocomposites for the synthesis of compound **4a**.

Cycle	Yield * (%)
1	98
2	95
3	94
4	91
5	90

* Reaction conditions: benzaldehyde (1 mmol), malononitrile (1 mmol), sodium azide (1.1 mmol), ethanol (5 mL), Fe_2_O_3_@cellulose@Mn nanocatalyst (15 mg), 100 watts.

**Table 5:** Optimization of reaction time for the synthesis of (*E*)-1-aryl-2-(1H-tetrazol-5-yl)acrylonitrile derivatives **4a**–**m**.

Entry	Time (min)	Yield * (%)
1	1	75
2	2	80
3	3	95
4	4	96
5	5	94

* Yield refers to the cumulative production output of all crops. Reaction conditions: benzaldehyde (1 mmol), malononitrile (1 mmol), sodium azide (1.1 mmol), ethanol (5 mL), Fe_2_O_3_@cellulose@Mn nanocatalyst (15 mg), 100 watts.

**Table 6:** Synthesis of (*E*)-1-aryl-2-(1H-tetrazol-5-yl)acrylonitrile derivatives **4a**–**m** using Fe_2_O_3_@cellulose@Mn in ethanol under MW irradiations.

Entry	Aldehydes (1a–m)	Product (4a–m)	Time (min)	Yield ^a^ (%)	Obs. mp (°C)	Lit. mp (°C)	References
1	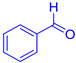 **(1a)**	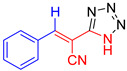 **(4a)**	3	98	165–167	168–170	[8]
2	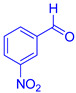 **(1b)**	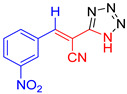 **(4b)**	2	97	161–163	159–163	[40]
3	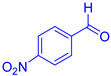 **(1c)**	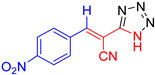 **(4c)**	2	96	168–169	166–168	[41]
4	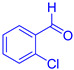 **(1d)**	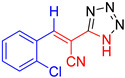 **(4d)**	3	97	171–173	175–177	[42]
5	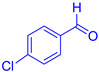 **(1e)**	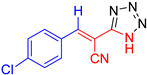 **(4e)**	3	95	160–161	158–159	[40]
6	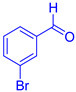 **(1f)**	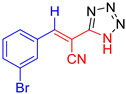 **(4f)**	3	96	163–164	165–167	[41]
7	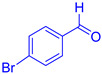 **(1g)**	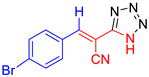 **(4g)**	3	93	165–167	168–169	[8]
8	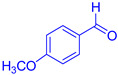 **(1h)**	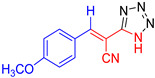 **(4h)**	2	97	152–153	153–155	[41]
9	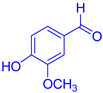 **(1i)**	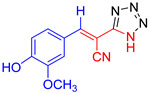 **(4i)**	3	96	161–164	159–161	[43]
10	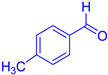 **(1j)**	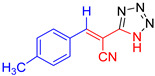 **(4j)**	3	95	188–190	189–191	[40]
11	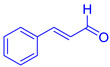 **(1k)**	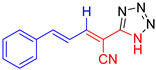 **(4k)**	3	98	167–169	168–170	[8]
12	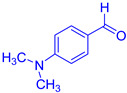 **(1l)**	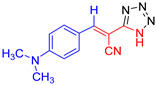 **(4l)**	2	94	170–171	171–172	[41]
13	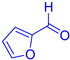 **(1m)**	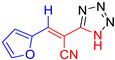 **(4m)**	3	98	87–89	85–86	[40]

^a^ Yield refers to the cumulative production output of all crops. Reaction conditions: benzaldehyde (1 mmol), malononitrile (1 mmol), sodium azide (1.1 mmol), ethanol (5 mL), Fe_2_O_3_@cellulose@Mn nanocatalyst (15 mg), 100 watts.

**Table 7:** Minimum inhibitory concentration (MIC in μg/mL) of synthesized (*E*)-1-aryl-2-(1H-tetrazol-5-yl)acrylonitrile derivatives **4a**–**m** against various microbial agents.

Entry	Gram (−ve) Bacteria		Gram (+ve) Bacteria			Fungi		
	*K. pneumonia*	*E. coli*	*S. pyogenes*	*B. subtilis*	*A. sclerotiorum*	*A. janus*	*S. aureus*	*A. niger*
**4a**	8	8	8	16	8	8	16	4
**4b**	16	8	16	16	4	4	4	8
**4c**	4	8	4	8	4	4	8	4
**4d**	8	4	4	4	8	16	8	8
**4e**	8	8	16	16	–	8	16	16
**4f**	8	16	8	8	8	16	16	16
**4g**	8	8	32	16	–	8	4	16
**4h**	16	32	16	64	16	32	-	64
**4i**	32	32	32	16	4	8	8	4
**4j**	64	8	16	32	32	64	64	16
**4k**	32	16	16	8	8	16	8	8
**4l**	16	8	8	8	8	8	8	16
**4m**	32	16	16	16	4	4	4	4
Cefixime	4	4	4	4	–	–	4	–
Fluconazole	–	–	–	–	2	2	–	2

**Table 8:** Comparison of Fe_2_O_3_@cellulose@Mn nanocatalyst with other heterogeneous catalysts in the synthesis of tetrazole derivatives.

Entry	Catalyst	Catalyst Loading (mol%)	Conditions	Time (min)	Yield 4a (%)	Reference
**1**	Silica molybdic acid	0.1	H_2_O/MW/70 °C	20	91	[43]
**2**	Fe_3_O_4_@PMO-ICS-ZnO	10	EtOH/reflux	180	98	[36]
**3**	Fe_3_O_4_@fibroin-SO_3_H	10	Solvent-free/100 °C	40	89	[45]
**4**	Fe_2_O_3_@cellulose@Mn	0.003	EtOH/MW/120 W	3	98	This work

## Data Availability

All data generated or analyzed during this study are included in this published article (and its Supplementary Materials).

## Supplementary Materials

The following supporting information can be downloaded at: https://www.mdpi.com/article/10.3390/molecules29184339/s1, Scheme S1: Graphical illustration for the synthesis of Fe_2_O_3_@cellulose@Mn nanocomposite; Scheme S2: Synthesis of the (*E*)-1-aryl-2-(1H-tetrazol-5-yl)acrylonitrile derivatives **4a**–**m** using Fe_2_O_3_@cellulose@Mn under MW irradiations; Table S1: Synthesis of (*E*)-1-aryl-2-(1H-tetrazol-5-yl)acrylonitrile derivatives **4a**–**m** using Fe_2_O_3_@cellulose@Mn under MW irradiations; Figure S1: FT-IR spectra of cellulose; Figure S2: FT-IR spectra of Fe2O3@cellulose@Mn nanocomposite; Figure S3: SEM images of (a–c) cellulose, (d–f) Fe_2_O_3_, and (g–i) Fe_2_O_3_@cellulose@Mn; Figure S4: EDS characterization of (a) Fe_2_O_3_ and (b) Fe_2_O_3_@cellulose@Mn; Figure S5: XRD characterization of Fe_2_O_3_ and Fe_2_O_3_@cellulose@Mn nanocomposite; Figure S6: VSM characterization of Fe_2_O_3_ and Fe_2_O_3_@cellulose@Mn nanocomposite; Figure S7: FT-IR spectra of (*Z*)-3-phenyl-2-(1H-tetrazol-5-yl)acrylonitrile (**4a**); Figure S8: 1H NMR spectra of (*Z*)-3-phenyl-2-(1H-tetrazol-5-yl)acrylonitrile (**4a**); Figure S9: 1H NMR spectra of (*Z*)-3-phenyl-2-(1H-tetrazol-5-yl)acrylonitrile (**4a**); Figure S10: 13C NMR spectra of (*Z*)-3-phenyl-2-(1H-tetrazol-5-yl)acrylonitrile (**4a**); Figure S11: FT-IR spectra of (*Z*)-3-(3-nitrophenyl)-2-(1H-tetrazol-5-yl)acrylonitrile (**4b**); Figure S12: 1H NMR spectra of (*Z*)-3-(3-nitrophenyl)-2-(1H-tetrazol-5-yl)acrylonitrile (**4b**); Figure S13: 1H NMR spectra of (*Z*)-3-(3-nitrophenyl)-2-(1H-tetrazol-5-yl)acrylonitrile (**4b**); Figure S14: 13C NMR spectra of (*Z*)-3-(3-nitrophenyl)-2-(1H-tetrazol-5-yl)acrylonitrile (**4b**); Figure S15: FT-IR spectra of (*Z*)-3-(4-nitrophenyl)-2-(1H-tetrazol-5-yl)acrylonitrile (**4c**); Figure S16: 1H-NMR spectra of (*Z*)-3-(4-nitrophenyl)-2-(1H-tetrazol-5-yl)acrylonitrile (**4c**); Figure S17: 1H-NMR expanded spectra of (*Z*)-3-(4-nitrophenyl)-2-(1H-tetrazol-5-yl)acrylonitrile (**4c**); Figure S18: 13C-NMR spectra of (*Z*)-3-(4-nitrophenyl)-2-(1H-tetrazol-5-yl)acrylonitrile (**4c**); Figure S19: FT-IR spectra of (*Z*)-3-(2-chlorophenyl)-2-(1H-tetrazol-5-yl)acrylonitrile (**4d**); Figure S20: 1H NMR spectra of (*Z*)-3-(2-chlorophenyl)-2-(1H-tetrazol-5-yl)acrylonitrile (**4d**); Figure S21: 1H NMR expanded spectra of (*Z*)-3-(2-chlorophenyl)-2-(1H-tetrazol-5-yl)acrylonitrile (**4d**); Figure S22: 13C NMR spectra of (*Z*)-3-(2-chlorophenyl)-2-(1H-tetrazol-5-yl)acrylonitrile (**4d**); Figure S23: FT-IR spectra of (*Z*)-3-(4-chlorophenyl)-2-(1H-tetrazol-5-yl)acrylonitrile (**4e**); Figure S24: 1H NMR spectra of (*Z*)-3-(4-chlorophenyl)-2-(1H-tetrazol-5-yl)acrylonitrile (**4e**); Figure S25: 1H NMR expanded spectra of (*Z*)-3-(4-chlorophenyl)-2-(1H-tetrazol-5-yl)acrylonitrile (**4e**); Figure S26: 13C NMR spectra of (*Z*)-3-(4-chlorophenyl)-2-(1H-tetrazol-5-yl)acrylonitrile (**4e**); Figure S27: FT-IR spectra of (*Z*)-3-(3-bromophenyl)-2-(1H-tetrazol-5-yl)acrylonitrile (**4f**); Figure S28: 1H NMR spectra of (*Z*)-3-(3-bromophenyl)-2-(1H-tetrazol-5-yl)acrylonitrile (**4f**); Figure S29: 1H NMR expanded spectra of (*Z*)-3-(3-bromophenyl)-2-(1H-tetrazol-5-yl)acrylonitrile (**4f**); Figure S30: 13C NMR spectra of (*Z*)-3-(3-bromophenyl)-2-(1H-tetrazol-5-yl)acrylonitrile (**4f**); Figure S31: FT-IR spectra of (*Z*)-3-(4-bromophenyl)-2-(1H-tetrazol-5-yl)acrylonitrile (**4g**); Figure S32: 1H-NMR spectra of (*Z*)-3-(4-bromophenyl)-2-(1H-tetrazol-5-yl)acrylonitrile (**4g**); Figure S33: 1H-NMR expanded spectra of (*Z*)-3-(4-bromophenyl)-2-(1H-tetrazol-5-yl)acrylonitrile (**4g**); Figure S34: 13C-NMR spectra of (*Z*)-3-(4-bromophenyl)-2-(1H-tetrazol-5-yl)acrylonitrile (**4g**); Figure S35: FT-IR spectra of (*Z*)-3-(4-methoxyphenyl)-2-(1H-tetrazol-5-yl)acrylonitrile (**4h**); Figure S36: 1H-NMR spectra of (*Z*)-3-(4-methoxyphenyl)-2-(1H-tetrazol-5-yl)acrylonitrile (**4h**); Figure S37: 1H-NMR expanded spectra of (*Z*)-3-(4-methoxyphenyl)-2-(1H-tetrazol-5-yl)acrylonitrile (**4h**); Figure S38: 13C-NMR spectra of (*Z*)-3-(4-methoxyphenyl)-2-(1H-tetrazol-5-yl)acrylonitrile (**4h**); Figure S39: FT-IR spectra of (*Z*)-3-(4-hydroxy-3-methoxyphenyl)-2-(1H-tetrazol-5-yl)acrylonitrile (**4i**); Figure S40: 1H-NMR spectra of (*Z*)-3-(4-hydroxy-3-methoxyphenyl)-2-(1H-tetrazol-5-yl)acrylonitrile (**4i**); Figure S41: 1H-NMR expanded spectra of (*Z*)-3-(4-hydroxy-3-methoxyphenyl)-2-(1H-tetrazol-5-yl)acrylonitrile (**4i**); Figure S42: 13C-NMR spectra of (*Z*)-3-(4-hydroxy-3-methoxyphenyl)-2-(1H-tetrazol-5-yl)acrylonitrile (**4i**); Figure S43: FT-IR spectra of (*Z*)-3-(4-methoxyphenyl)-2-(1H-tetrazol-5-yl)acrylonitrile (**4j**); Figure S44: 1H-NMR spectra of (*Z*)-3-(4-methoxyphenyl)-2-(1H-tetrazol-5-yl)acrylonitrile (**4j**); Figure S45: 1H-NMR expanded spectra of (*Z*)-3-(4-methoxyphenyl)-2-(1H-tetrazol-5-yl)acrylonitrile (**4j**); Figure S46: 13C-NMR spectra of (*Z*)-3-(4-methoxyphenyl)-2-(1H-tetrazol-5-yl)acrylonitrile (**4j**); Figure S47: FT-IR spectra of (2Z,**4e**)-5-phenyl-2-(1H-tetrazol-5-yl)penta-2,4-dienenitrile (**4k**); Figure S48: 1H-NMR spectra of (2Z,**4e**)-5-phenyl-2-(1H-tetrazol-5-yl)penta-2,4-dienenitrile (**4k**); Figure S49: 1H-NMR expanded spectra of (2Z,**4e**)-5-phenyl-2-(1H-tetrazol-5-yl)penta-2,4-dienenitrile (**4k**); Figure S50: 13C-NMR spectra of (2Z,**4e**)-5-phenyl-2-(1H-tetrazol-5-yl)penta-2,4-dienenitrile (**4k**); Figure S51: FT-IR spectra of (*Z*)-3-(4-(dimethylamino)phenyl)-2-(1H-tetrazol-5-yl)acrylonitrile (**4l**); Figure S52: 1H-NMR spectra of (*Z*)-3-(4-(dimethylamino)phenyl)-2-(1H-tetrazol-5-yl)acrylonitrile (**4l**); Figure S53: 1H-NMR expanded spectra of (*Z*)-3-(4-(dimethylamino)phenyl)-2-(1H-tetrazol-5-yl)acrylonitrile (**4l**); Figure S54: 13C-NMR spectra of (*Z*)-3-(4-(dimethylamino)phenyl)-2-(1H-tetrazol-5-yl)acrylonitrile (**4l**); Figure S55: FT-IR spectra of (*Z*)-3-(furan-2-yl)-2-(1H-tetrazol-5-yl)acrylonitrile (**4m**); Figure S56: 1H-NMR spectra of (*Z*)-3-(furan-2-yl)-2-(1H-tetrazol-5-yl)acrylonitrile (**4m**); Figure S57: 1H-NMR expanded spectra of (*Z*)-3-(furan-2-yl)-2-(1H-tetrazol-5-yl)acrylonitrile (**4m**); Figure S58: 13C-NMR spectra of (*Z*)-3-(furan-2-yl)-2-(1H-tetrazol-5-yl)acrylonitrile (**4m**); Figure S59: Structures of (*Z*)-3-phenyl-2-(1H-tetrazol-5-yl)acrylonitrile **5a**–**m**.
