# Supplementary material for: Synthesis and Antimicrobial Activity of (E)-1-Aryl-2-(1H-tetrazol-5-yl)acrylonitrile Derivatives via [3+2] Cycloaddition Reaction Using Reusable Heterogeneous Nanocatalyst under Microwave Irradiation
Source: Molecules. 2024 Sep 12;29(18):4339. doi: 10.3390/molecules29184339 (PMC11434072; doi:10.3390/molecules29184339)
Supplement: Supplementary file 1 [file molecules-29-04339-s001.zip › molecules-3175529-supplementary.pdf]

Supplementary Information file

1. Characterization of *(Z)-3-phenyl-2-(1H-tetrazol-5-yl)acrylonitrile* 4a using Fe_2O_3@cellulose@Mn nanocomposite

(Z)-3-phenyl-2-(1H-tetrazol-5-yl)acrylonitrile (4a): Yield 98%; pale yellow crystals; MP: 165-167 ºC, Lit. MP: 168-170 ºC; FT-IR (ν, cm^{-1}): 3252 (NH), 3032.93 (C-H), 2222.61 (C≡N). ^1H NMR spectrum, (δ, ppm, $CDCl_3$, 500 MHz): 7.91-7.89 (d, 1H, Ar-H), 7.78 (s, 1H, CH), 7.65-7.61 (t, 2H, Ar-H), 7.55-7.52 (t, 2H, Ar-H), 3.1 (*br.s*, 1H, NH, exchangeable). ^{13}C NMR spectrum, (δ, ppm, $CDCl_3$, 125 MHz): 159.05 (C-1), 133.62 (C-1'), 129.91 (C-2'& C-6'), 129.71 (C-3'& C-5'), 128.61 (C-4'), 112.74 (C≡N), 111.59 (C-3), 81.70 (C-2). Anal. calcd. for $C_{10}H_7N_5$: C, 60.91%; H, 3.58%; N, 35.51%. Found: C, 60.89%; H, 3.55%; N, 35.56%; ESI-MS(*m/z*); M+1 = 198.07.

(Z)-3-(3-nitrophenyl)-2-(1H-tetrazol-5-yl)acrylonitrile (4b): Yield 97%; pale yellow crystals; MP: 161-163 ºC, Lit. MP: 159-163 ºC; FT-IR (ν, cm^{-1}): 3268 (NH), 3084.98 (C-H), 2225.32 (C≡N), 1523.41 (NO_2). ^1H NMR spectrum, (δ, ppm, $CDCl_3$, 500 MHz): 8.78-8.77 (s, 1H, Ar-H), 8.72 (s, 1H, CH), 8.50-8.48 (d, 1H, Ar-H), 8.34-8.32 (d, 1H, Ar-H), 7.94-7.90 (t, 1H, Ar-H), 3.32 (*br.s*, 1H, NH, exchangeable). ^{13}C NMR spectrum, (δ, ppm, $CDCl_3$, 125 MHz): 159.18 (C-1), 147.92 (C-1'), 135.74 (C-2'), 132.34 (C-3'), 131.05 (C-4'), 127.80 (C-5'), 124.72 (C-6'), 113.54 (C≡N), 112.49 (C-3), 84.83 (C-2). Anal. calcd. for $C_{10}H_6N_6O_2$: C, 49.59%; H, 2.50%; N, 34.70%, O, 13.21%. Found: C, 49.50%; H, 2.53%; N, 34.77%, O, 13.20%; ESI-MS(*m/z*); M+1 = 243.05.

(Z)-3-(4-nitrophenyl)-2-(1H-tetrazol-5-yl)acrylonitrile (4c): Yield 96%; yellow crystal; MP: 168-169 ºC, Lit. MP: 166-168 ºC; FT-IR (ν, cm^{-1}): 3264 (NH), 3039.99 (C-H), 2230.32 (C≡N), 1504.70 (NO_2). ^1H NMR spectrum, (δ, ppm, $CDCl_3$, 500 MHz): 8.40-8.37 (d, 2H, Ar-H), 8.09-8.06 (d, 2H, Ar-H), 7.89 (s, 1H, CH), 3.52 (*br.s*, 1H, NH, exchangeable). ^{13}C NMR spectrum, (δ, ppm, $CDCl_3$, 125 MHz): 156.87 (C-1), 150.39 (C-1'), 135.81 (C-2' & C-6'), 131.32 (C-3'& C-5'), 124.66 (C-4'), 112.63 (C≡N), 111.60 (C-3), 87.57 (C-2). Anal. calcd. for $C_{10}H_6N_6O_2$: C, 49.59%; H, 2.50%; N, 34.70%, O, 13.21%. Found: C, 49.50%; H, 2.53%; N, 34.77%, O, 13.20%; ESI-MS(*m/z*); M+1 = 243.05.

(Z)-3-(2-chlorophenyl)-2-(1H-tetrazol-5-yl)acrylonitrile (4d)**:** Yield 97%; light yellow crystal; MP: 171-173 ºC, Lit. MP: 175-177 ºC; FT-IR (ν, cm^{-1}): 3225 (NH), 3077.75 (C-H), 2223.53 (C≡N), 820.68 (C-Cl). ^{1}H NMR spectrum, (δ, ppm, $CDCl_3$, 500 MHz): 8.25 (s, 1H, CH), 8.17-8.16 (d, 1H, Ar-H), 7.55-7.53 (d, 1H, Ar-H), 7.46-7.42 (t, 2H, Ar-H), 3.23 (*br.s*, 1H, NH, exchangeable). ^{13}C NMR spectrum, (δ, ppm, $CDCl_3$, 125 MHz): 156.10 (C-1), 136.31 (C-6'), 135.10 (C-5'), 130.73 (C-1'), 129.51 (C-4'), 129.08 (C-2'), 127.83 (C-3'), 113.27 (C≡N), 111.97 (C-3), 85.76 (C-2). Anal. calcd. for $C_{10}H_6ClN_5$: C, 51.85%; H, 2.61%; Cl, 15.31%; N, 30.23. Found: C, 51.80%; H, 2.63%; Cl, 15.32%; N, 30.25; ESI-MS(*m/z*); M+1 = 233.02.

(Z)-3-(4-chlorophenyl)-2-(1H-tetrazol-5-yl)acrylonitrile (4e)**:** Yield 95%; pale yellow crystal; MP: 160-161 ºC, Lit. MP: 158-159 ºC; FT-IR (ν, cm^{-1}): 3220 (NH), 3090.93 (C-H), 2223.04 (C≡N), 827.28 (C-Cl). ^{1}H NMR spectrum, (δ, ppm, $CDCl_3$, 500 MHz): 7.86-7.84 (d, 2H, Ar-H), 7.74 (s, 1H, CH), 7.53-7.50 (d, 2H, Ar-H), 3.47 (*br.s*, 1H, NH, exchangeable). ^{13}C NMR spectrum, (δ, ppm, $CDCl_3$, 125 MHz): 158.30 (C-1), 141.16 (C-1'), 131.85 (C-2'& C-6'), 130.08 (C-3'& C-5'), 129.29 (C-4'), 113.45 (C≡N), 112.35 (C-3), 83.36 (C-2). Anal. calcd. for $C_{10}H_6ClN_5$: C, 51.85%; H, 2.61%; Cl, 15.31%; N, 30.23. Found: C, 51.80%; H, 2.63%; Cl, 15.32%; N, 30.25; ESI-MS(*m/z*); M+1 = 233.02.

(Z)-3-(3-bromophenyl)-2-(1H-tetrazol-5-yl)acrylonitrile (4f)**:** Yield 96%; yellow crystal; MP: 163-164 ºC, Lit. MP: 165-167 ºC; FT-IR (ν, cm^{-1}): 3209 (NH), 3061.96 (C-H), 2223.90 (C≡N), 673.51 (C-Br). ^{1}H NMR spectrum, (δ, ppm, $CDCl_3$, 500 MHz): 7.97-7.96 (s, 1H, Ar-H), 7.90-7.88 (d, 1H, A-H), 7.76-7.74 (d, 1H, Ar-H), 7.71 (s, 1H, CH), 3.23 (*br.s*, 1H, NH, exchangeable). ^{13}C NMR spectrum, (δ, ppm, $CDCl_3$, 125 MHz): 158.09 (C-1), 137.24 (C-1'), 133.43 (C-4'), 132.57(C-6'), 131.08 (C-3'), 128.63(C-2'), 123.64 (C-5'), 113.18 (C≡N), 112.00 (C-3), 84.68 (C-2). Anal. calcd. for $C_{10}H_6BrN_5$: C, 43.50%; H, 2.19%; Br, 28.94%; N, 25.37. Found: C, 43.51%; H, 2.21%; Br, 28.95%; N, 25.33; ESI-MS(*m/z*); M+1 = 276.98.

(Z)-3-(4-bromophenyl)-2-(1H-tetrazol-5-yl)acrylonitrile (4g)**:** Yield 93%; yellow crystal; MP: 165-167 ºC, Lit. MP: 168-169 ºC; FT-IR (ν, cm^{-1}): 3214 (NH), 2223.40 (C≡N), 673.80 (C-Br). ^{1}H NMR spectrum, (δ, ppm, $CDCl_3$, 500 MHz): 8.49 (s, 1H, CH), 7.85-7.82 (d, 2H, Ar-H), 7.81-7.79 (d, 2H, Ar-H), 3.16 (*br.s*, 1H, NH, exchangeable). ^{13}C NMR spectrum, (δ, ppm, $CDCl_3$, 125 MHz): 160.08 (C-

1), 132.50 (C-1’), 131.97 (C-2’& C-6’), 130.18 (C-3’& C-5’), 128.23 (C-4’), 113.89 (C≡N), 112.82 (C-3), 82.09 (C-2). Anal. calcd. for $C_{10}H_6BrN_5$: C, 43.50%; H, 2.19%; Br, 28.94%; N, 25.37. Found: C, 43.51%; H, 2.21%; Br, 28.95%; N, 25.33; ESI-MS(*m/z*); M+1 = 276.98.

(Z)-3-(4-methoxyphenyl)-2-(1H-tetrazol-5-yl)acrylonitrile (4h)**:** Yield 97%; pale yellow crystal; MP: 152-153 ºC, Lit. MP: 153-155 ºC; FT-IR (ν, cm^{-1}): 3265 (NH), 3096.10 (C-H), 2781.68 (C-OCH_3), 2223.39 (C≡N). ^{1}H NMR spectrum, (δ, ppm, $CDCl_3$, 500 MHz): 7.92-7.89 (d, 2H, Ar-H), 7.65 (s, 1H, CH), 7.03-7.00 (d,2H, Ar-H), 3.91 (*br.s*, 1H, NH, exchangeable). ^{13}C NMR spectrum, (δ, ppm, $CDCl_3$, 125 MHz): 164.85 (C-1), 158.91 (C-1’), 133.47 (C-2’& C-6’), 124.03 (C-3’& C-5’), 115.16 (C-4’), 114.46 (C≡N), 113.38 (C-3), 78.50 (C-2), 55.83 (OCH_3). Anal. calcd. for $C_{11}H_9N_5O$: C, 58.14%; H, 3.99%; N, 30.82%; O, 7.04%. Found: C, 58.16%; H, 3.98%; N, 30.84%; O, 7.01%; ESI-MS(*m/z*); M+1 = 228.08.

(Z)-3-(4-hydroxy-3-methoxyphenyl)-2-(1H-tetrazol-5-yl)acrylonitrile (4i)**:** Yield 96%; yellow crystal; MP: 161-164 ºC, Lit. MP: 159-161 ºC; FT-IR (ν, cm^{-1}): 3272 (NH), 3457.90 (OH), 2796.68 (OCH_3), 2223.41 (C≡N). ^{1}H NMR spectrum, (δ, ppm, $CDCl_3$, 500 MHz): 7.71 (s, 1H, Ar-H), 7.63 (s, 1H, CH), 7.32-7.30 (d, 1H, Ar-H), 7.02-7.00 (d, 1H, Ar-H), 6.43 (s, 1H, OH), 3.97 (s, 3H, OCH_3), 3.97 (*br.s*, 1H, NH, exchangeable). ^{13}C NMR spectrum, (δ, ppm, $CDCl_3$, 125 MHz): 159.29 (C-1), 152.18 (C-4’), 147.09 (C-5’), 128.97 (C-1’), 124.00 (C-2’), 115.26 (C-6’), 114.41 (C≡N), 113.64 (C-3), 110.53 (C-3’), 78.13 (C-2), 56.24 (OCH_3). Anal. calcd. for $C_{11}H_9N_5O_2$: C, 54.32%; H, 3.73%; N, 28.79%; O, 13.16%. Found: C, 54.33%; H, 3.74%; N, 28.78%; O, 13.15%; ESI-MS(*m/z*); M+1 = 244.07.

(Z)-2-(1H-tetrazol-5-yl)-3-p-tolylacrylonitrile (4j)**:** Yield 95%; cream crystal; MP: 188-190 ºC, Lit. MP: 189-191 ºC; FT-IR (ν, cm^{-1}): 3261 (NH), 3036.50 (C-CH_3), 2223.45 (C≡N). ^{1}H NMR spectrum, (δ, ppm, $CDCl_3$, 500 MHz): 7.81-7.80 (d, 2H, Ar-H), 7.72 (s, 1H, CH), 7.34-7.33 (d, 2H, Ar-H), 2.45 (s, 3H, Ar-H), 2.87 (*br.s*, 1H, NH, exchangeable). ^{13}C NMR spectrum, (δ, ppm, $CDCl_3$, 125 MHz): 159.83 (C-1), 146.41 (C-4’), 130.94 (C-3’ & C-5’), 130.40 (C-2’ & C-6’), 128.49 (C-1’), 114.04 (C≡N), 112.89 (C-3), 81.16 (C-2), 22.02 (CH_3). Anal. calcd. for $C_{11}H_9N_5$: C, 62.55%; H, 4.29%; N, 33.16%. Found: C, 62.54%; H, 4.28%; N, 33.18%; ESI-MS(*m/z*); M+1 = 212.09.

***(2Z,4E)-5-phenyl-2-(1H-tetrazol-5-yl)penta-2,4-dienenitrile (4k)*:** Yield 98%; pale yellow crystal; MP: 167-169 ºC, Lit. MP: 168-170 ºC; FT-IR (ν, cm^{-1}): 3228 (NH), 3031.32 (C-H), 2223.38 (C≡N). ^{1}H NMR spectrum, (δ, ppm, $CDCl_3$, 500 MHz): 7.58-7.60 (m, 2H, Ar-H), 7.42-7.48 (m, 2H, Ar-H), 7.20 (s, 1H, CH-1), 7.25-7.26 (d, 1H, Ar-H), 7.21-7.23 (t, 1H, CH-3), 3.26 (*br.s*, 1H, NH, exchangeable). ^{13}C NMR spectrum, (δ, ppm, $CDCl_3$, 125 MHz): 160.22 (C-1), 150.58 (C-2'), 133.99 (C-3'), 132.15 (C-6'), 129.38 (C-4'& C-8'), 129.02 (C-5'& C-7'), 122.29 (C-1'), 113.63 (C≡N), 111.78 (C-3), 82.85 (C-2). Anal. calcd. for $C_{12}H_9N_5$: C, 64.56%; H, 4.06%; N, 31.37%. Found: C, 64.55%; H, 4.05%; N, 31.39%; ESI-MS(*m/z*); M+1 = 224.09.

***(Z)-3-(4-(dimethylamino)phenyl)-2-(1H-tetrazol-5-yl)acrylonitrile (4l)*:** Yield 94%; pale yellow crystal; MP: 170-171 ºC, Lit. MP: 171-172 ºC; FT-IR (ν, cm^{-1}): 3278 (NH), 3036.50 ($C-CH_3$), 2223.36 (C≡N), 1310.45 (C-N). ^{1}H NMR spectrum, (δ, ppm, $CDCl_3$, 500 MHz): 7.63-7.60 (d, 2H, Ar-H), 7.72 (s, 1H, CH), 7.34-7.33 (d, 2H, Ar-H), 2.45 (s, 3H, Ar-H), 3.14 (*br.s*, 1H, NH, exchangeable). ^{13}C NMR spectrum, (δ, ppm, $CDCl_3$, 125 MHz): 158.53 (C-1), 146.41 (C-4'), 135.54 (C-3' & C-5'), 132.86 (C-2' & C-6'), 125.79 (C-1'), 113.54 (C≡N), 111.49 (C-3), 81.16(C-2), 36.96 ($N-CH_3$), 35.86 ($N-CH_3$). Anal. calcd. for $C_{12}H_{12}N_6$: C, 59.99%; H, 5.03%; N, 34.98%. Found: C, 59.97%; H, 5.04%; N, 34.99%; ESI-MS(*m/z*); M+1 = 241.12.

***(Z)-3-(furan-2-yl)-2-(1H-tetrazol-5-yl)acrylonitrile (4m)*:** Yield 98%; yellow crystal; MP: 87-89 ºC, Lit. MP: 85-86 ºC; FT-IR (ν, cm^{-1}): 3203 (NH), 3124.17 (C-O), 3040.48 (C-H), 2223.92 (C≡N). ^{1}H NMR spectrum, (δ, ppm, $CDCl_3$, 500 MHz): 7.82-7.81 (d, 1H, Ar-H), 7.54 (s, 1H, CH), 7.37-7.36 (d, 1H, Ar-H), 6.73-6.72 (t, 1H, Ar-H), 3.47 (*br.s*, 1H, NH, exchangeable). ^{13}C NMR spectrum, (δ, ppm, $CDCl_3$, 125 MHz): 149.67 (C-1'), 148.10 (C-1), 143.16 (C-4'), 123.69 (C-2'), 114.51 (C≡N), 113.89 (C-3), 112.67 (C-3'), 73.7 (C-2). Anal. calcd. for $C_8H_5N_5O$: C, 51.34%; H, 2.69%; N, 37.42%; O, 8.55%. Found: C, 51.32%; H, 2.68%; N, 37.43%; O, 8.57%; ESI-MS(*m/z*); M+1 = 188.05.

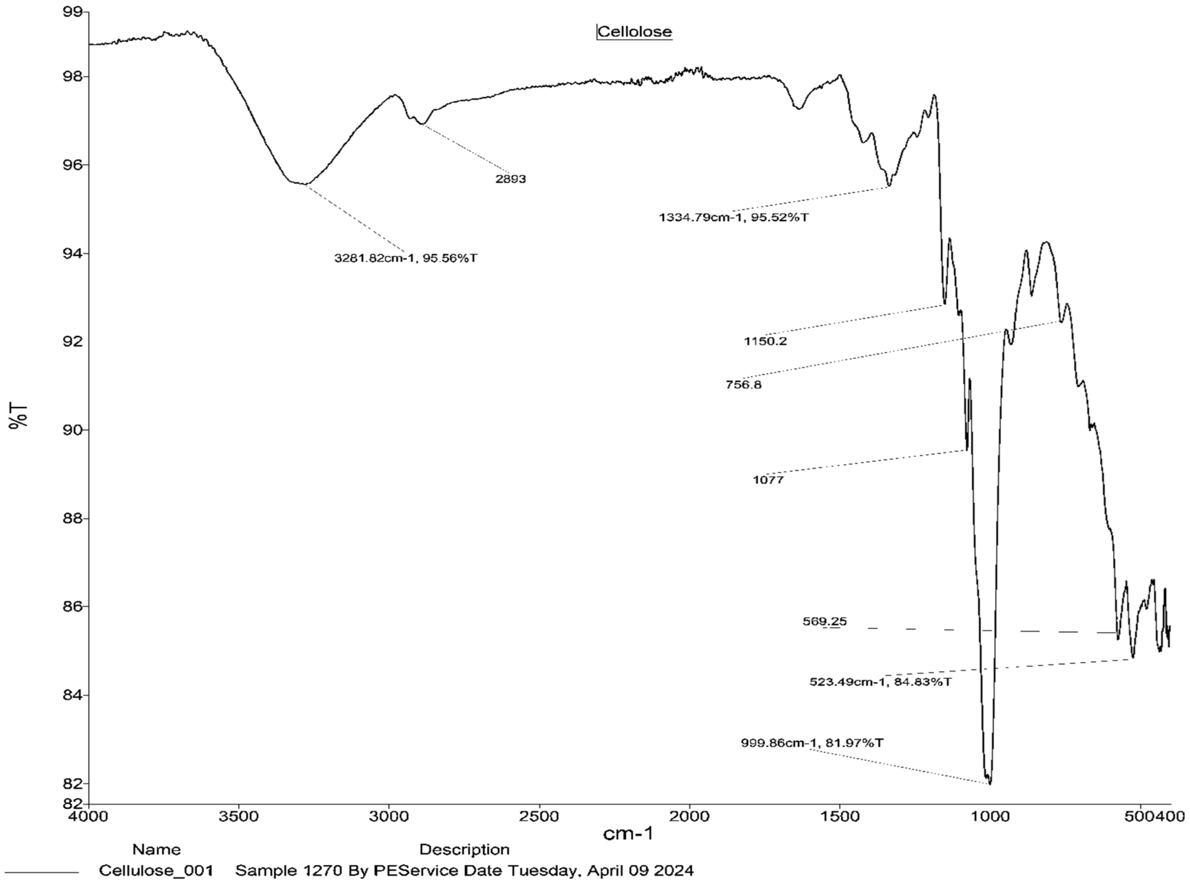

Figure S1: FTIR spectra of cellulose.

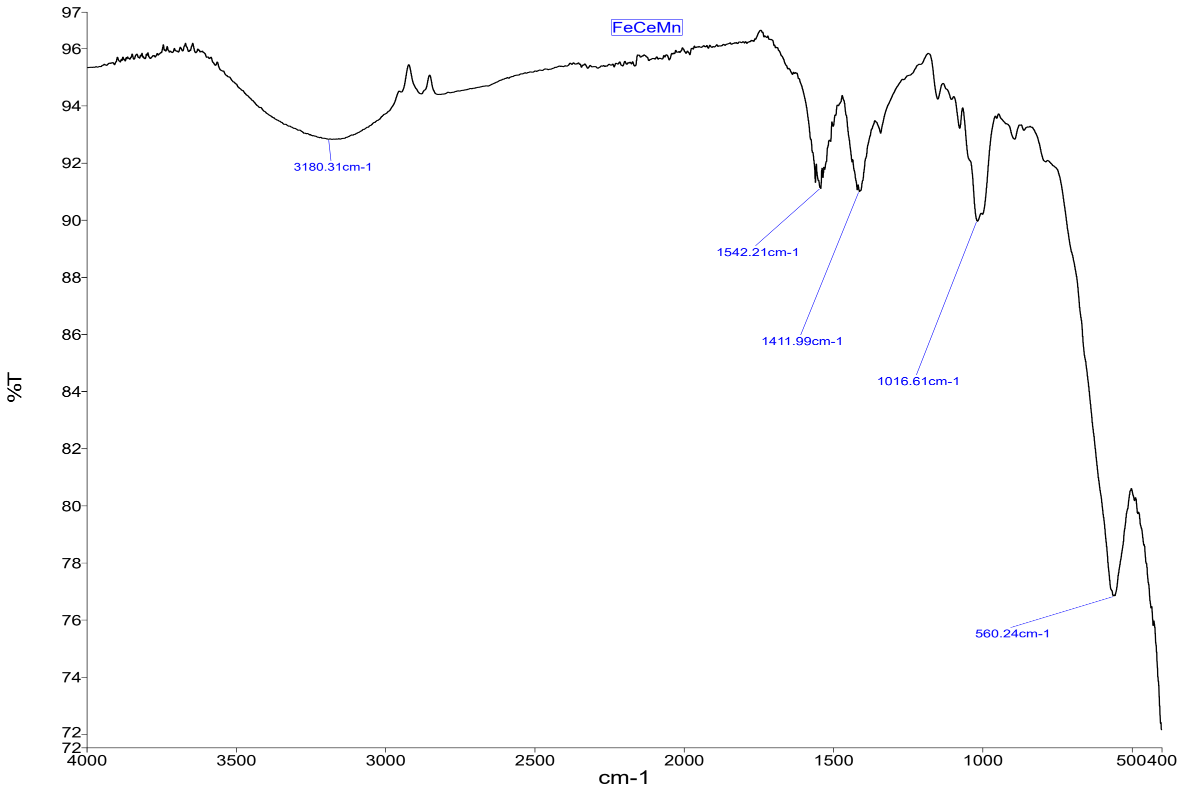

Figure S2: FTIR spectra of Fe_2O_3@cellulose@Mn nanocomposite.

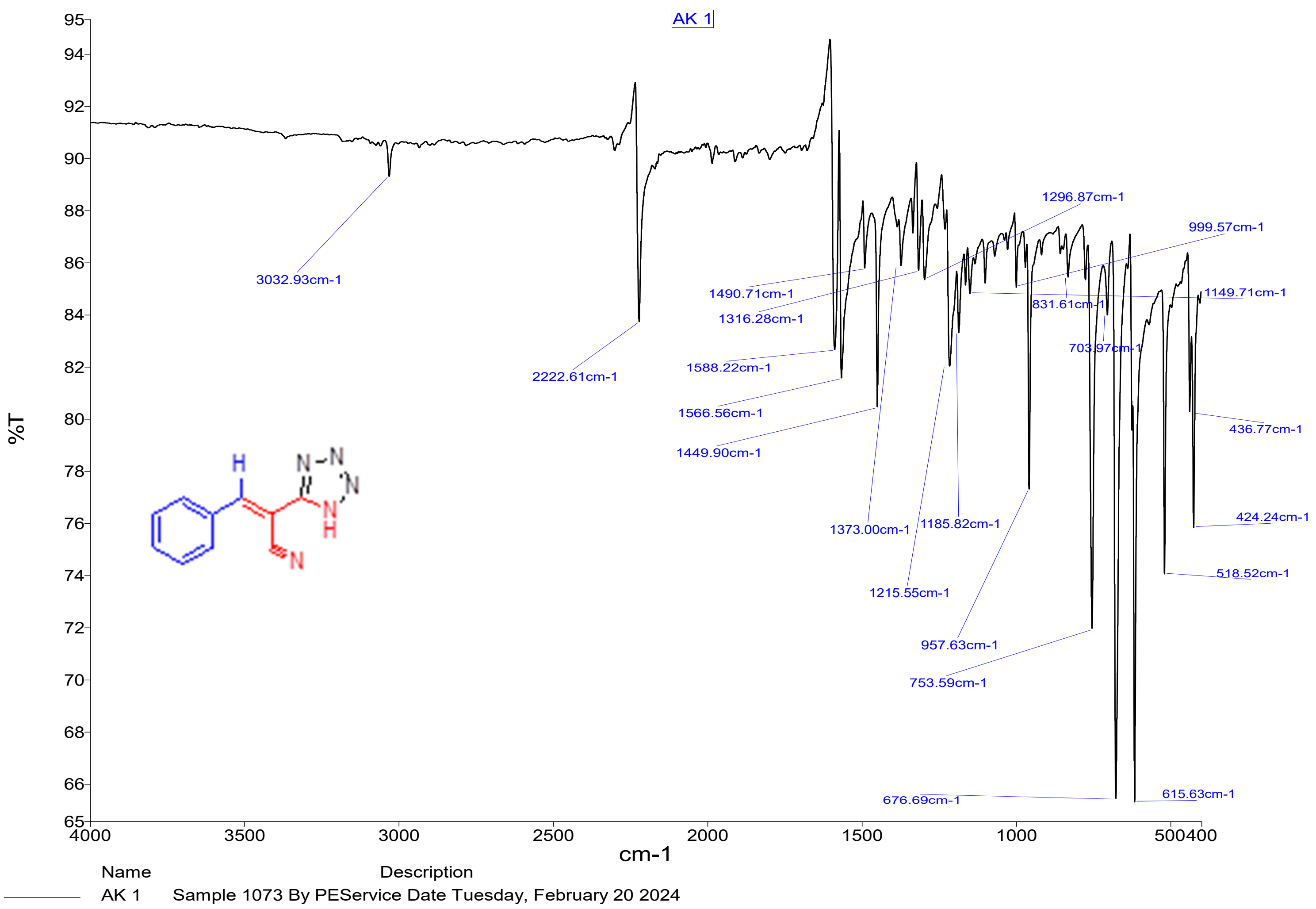

Figure S3: FTIR spectra of (Z)-3-phenyl-2-(1H-tetrazol-5-yl)acrylonitrile **(4a)**

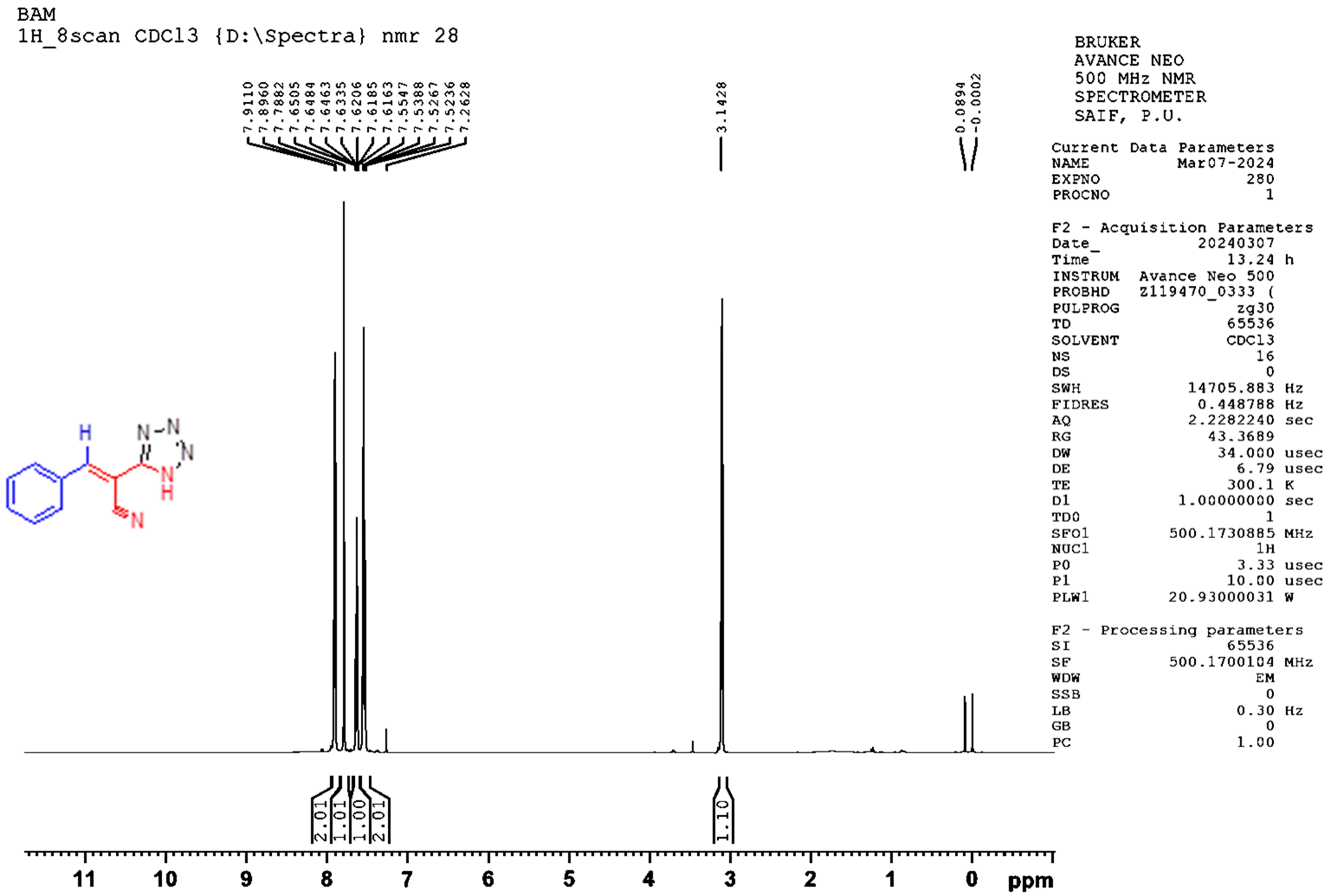

Figure S4: ^{1}H NMR spectra of (Z)-3-phenyl-2-(1H-tetrazol-5-yl)acrylonitrile **(4a)**

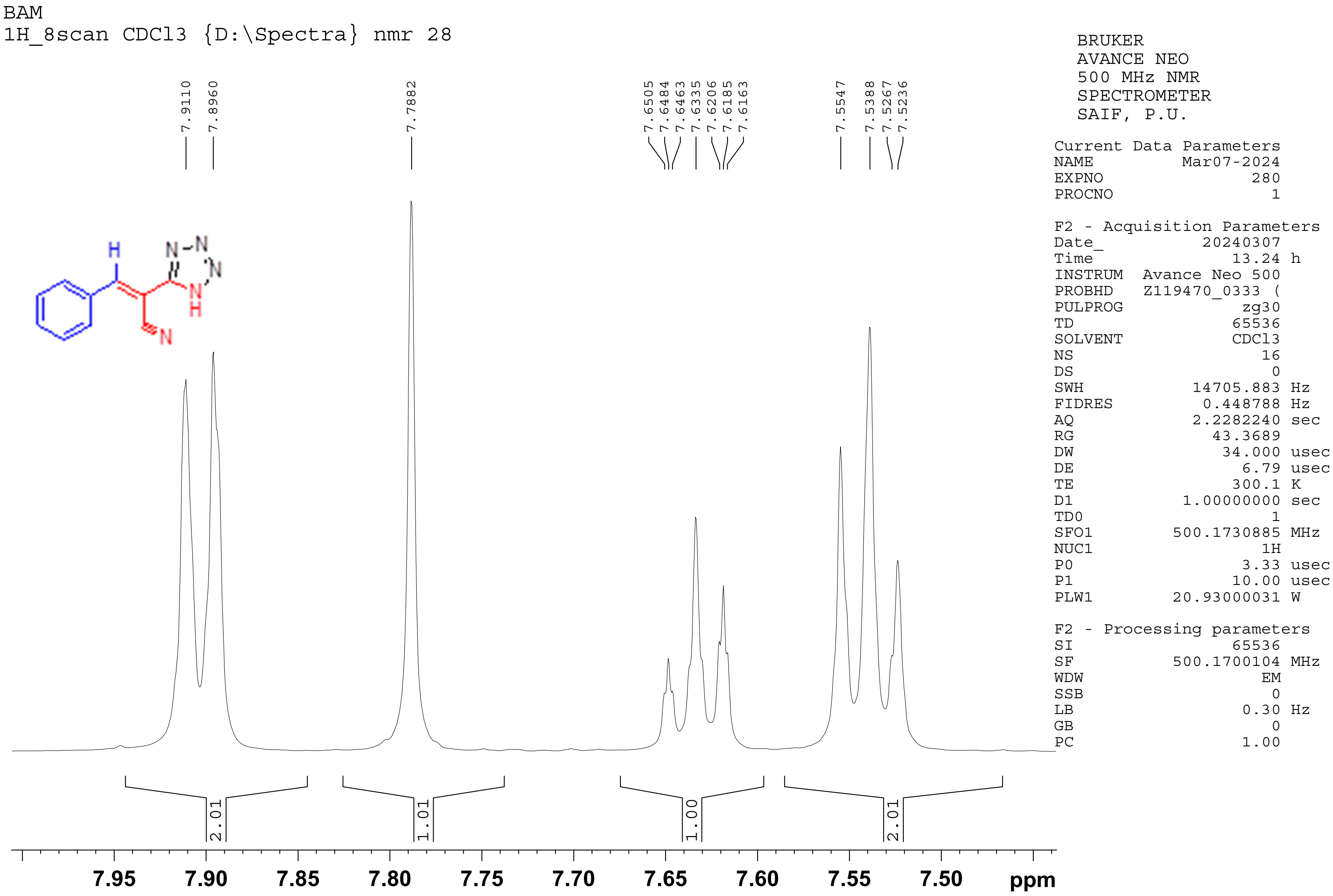

Figure S5: ^{1}H NMR expanded spectra of (Z)-3-phenyl-2-(1H-tetrazol-5-yl)acrylonitrile **(4a)**

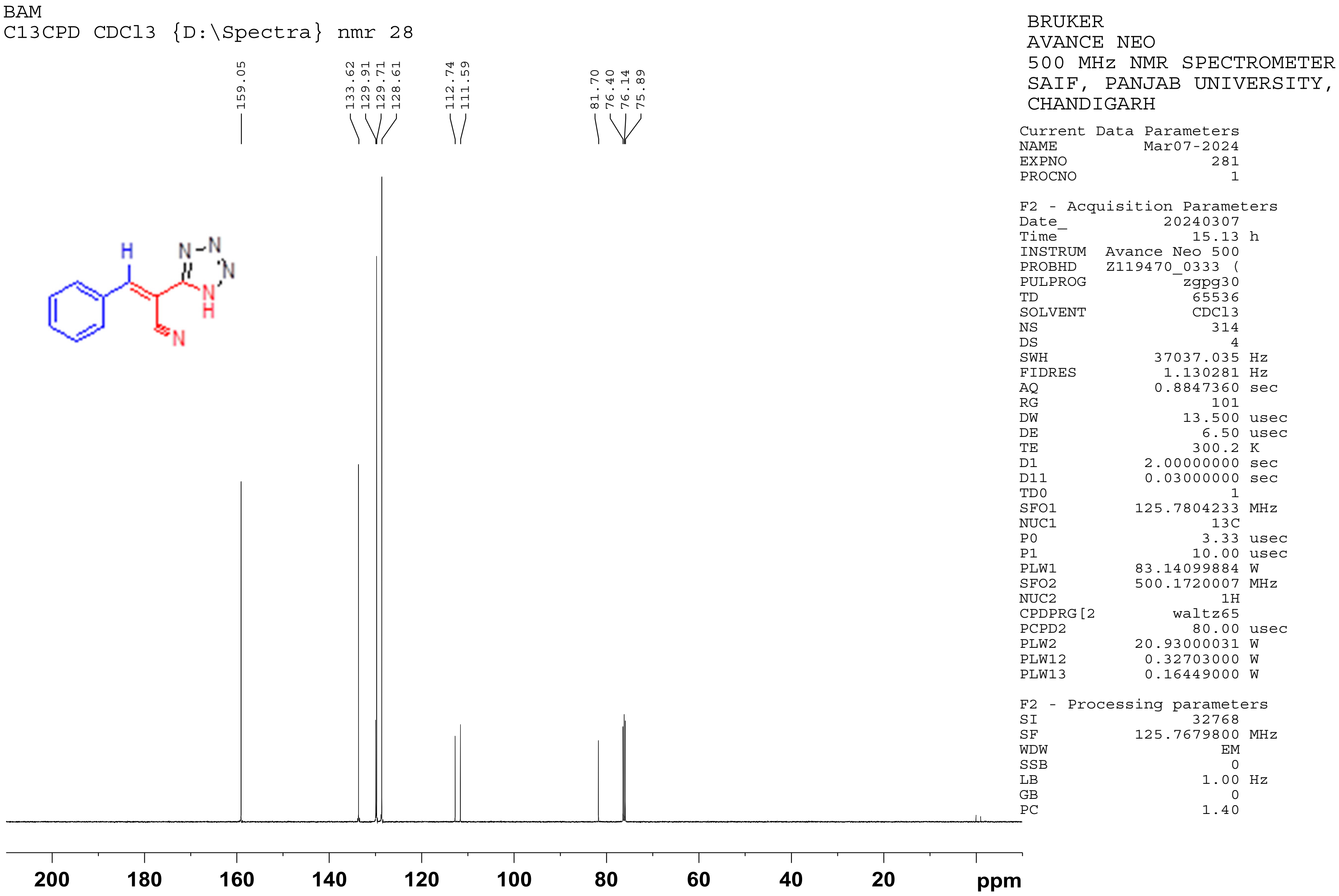

Figure S6: ^{13}C NMR spectra of (Z)-3-phenyl-2-(1H-tetrazol-5-yl)acrylonitrile **(4a)**

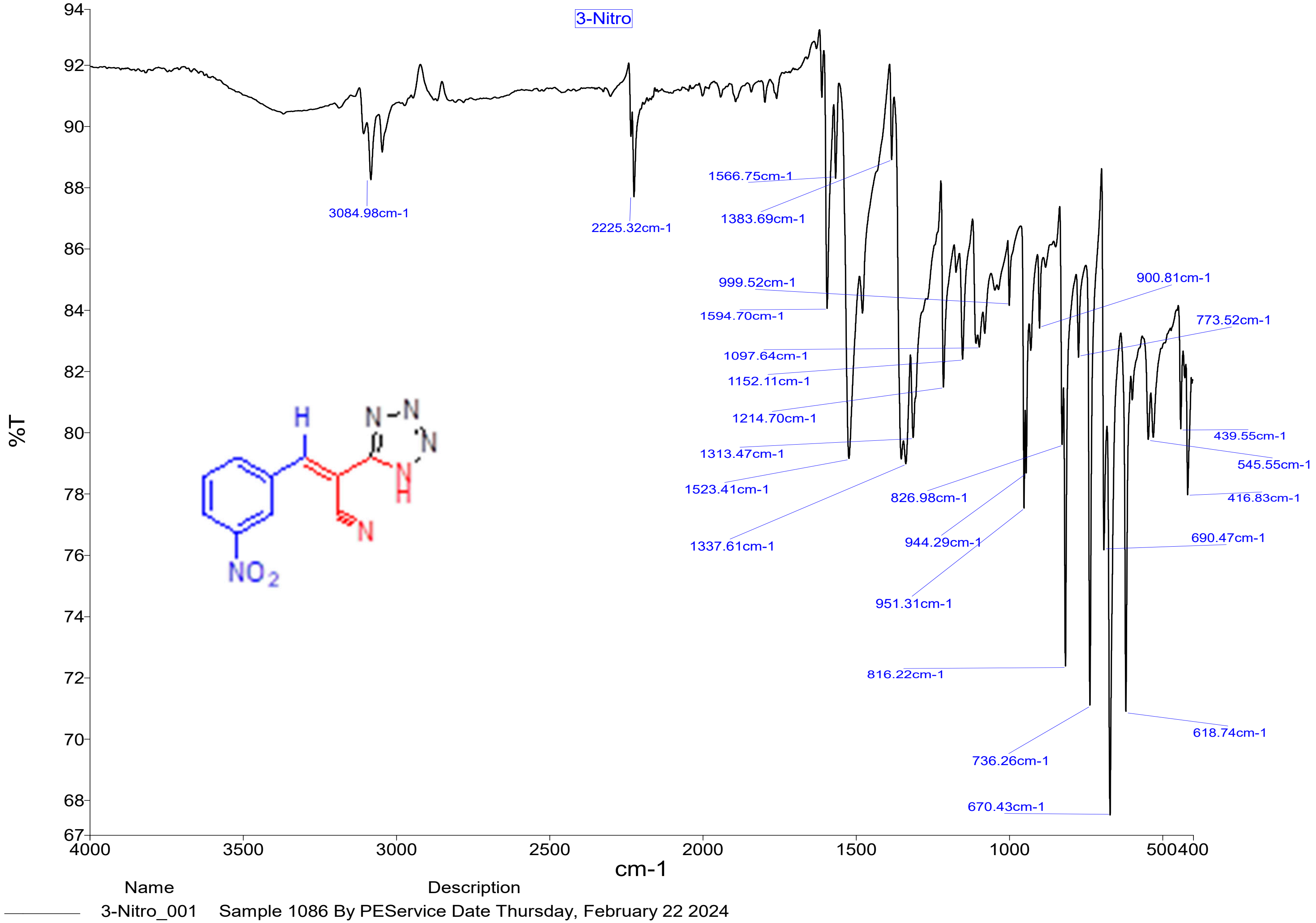

Figure S7: FT-IR spectra of (Z)-3-(3-nitrophenyl)-2-(1H-tetrazol-5-yl)acrylonitrile **(4b)**.

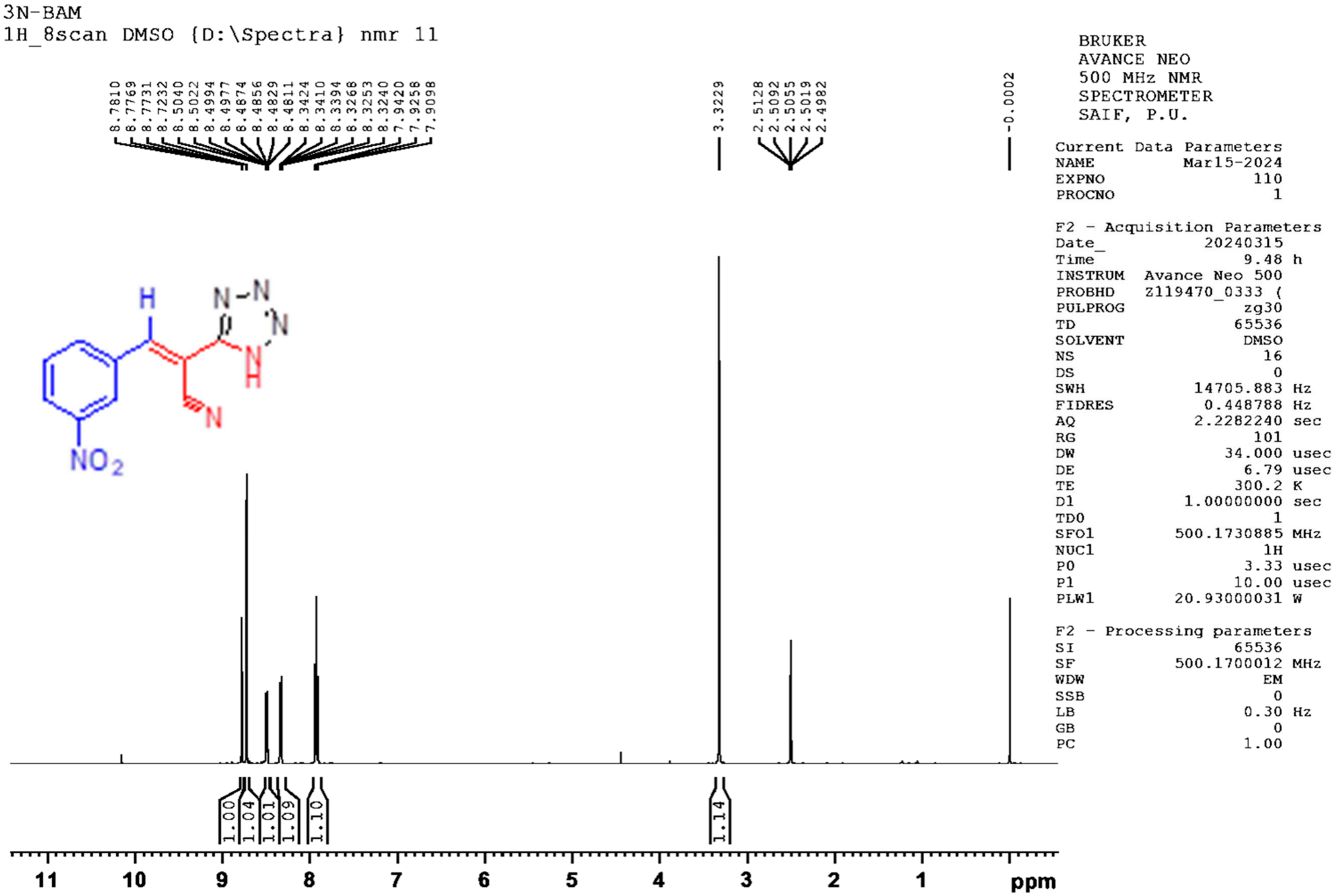

Figure S8: ^{1}H NMR spectra of (Z)-3-(3-nitrophenyl)-2-(1H-tetrazol-5-yl)acrylonitrile **(4b)**

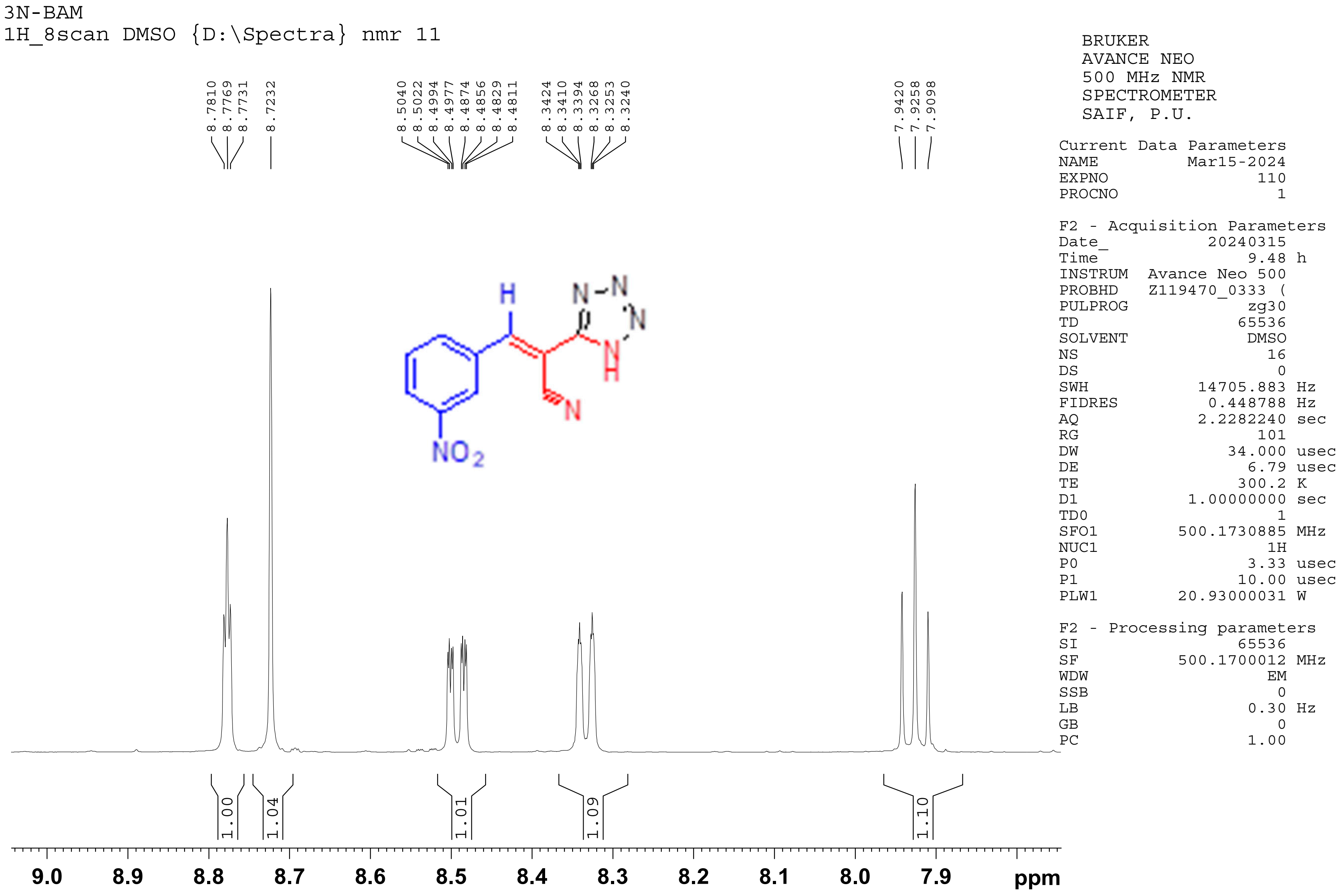

Figure S9: ^{1}H NMR expanded spectra of (Z)-3-(3-nitrophenyl)-2-(1H-tetrazol-5-yl)acrylonitrile **(4b)**

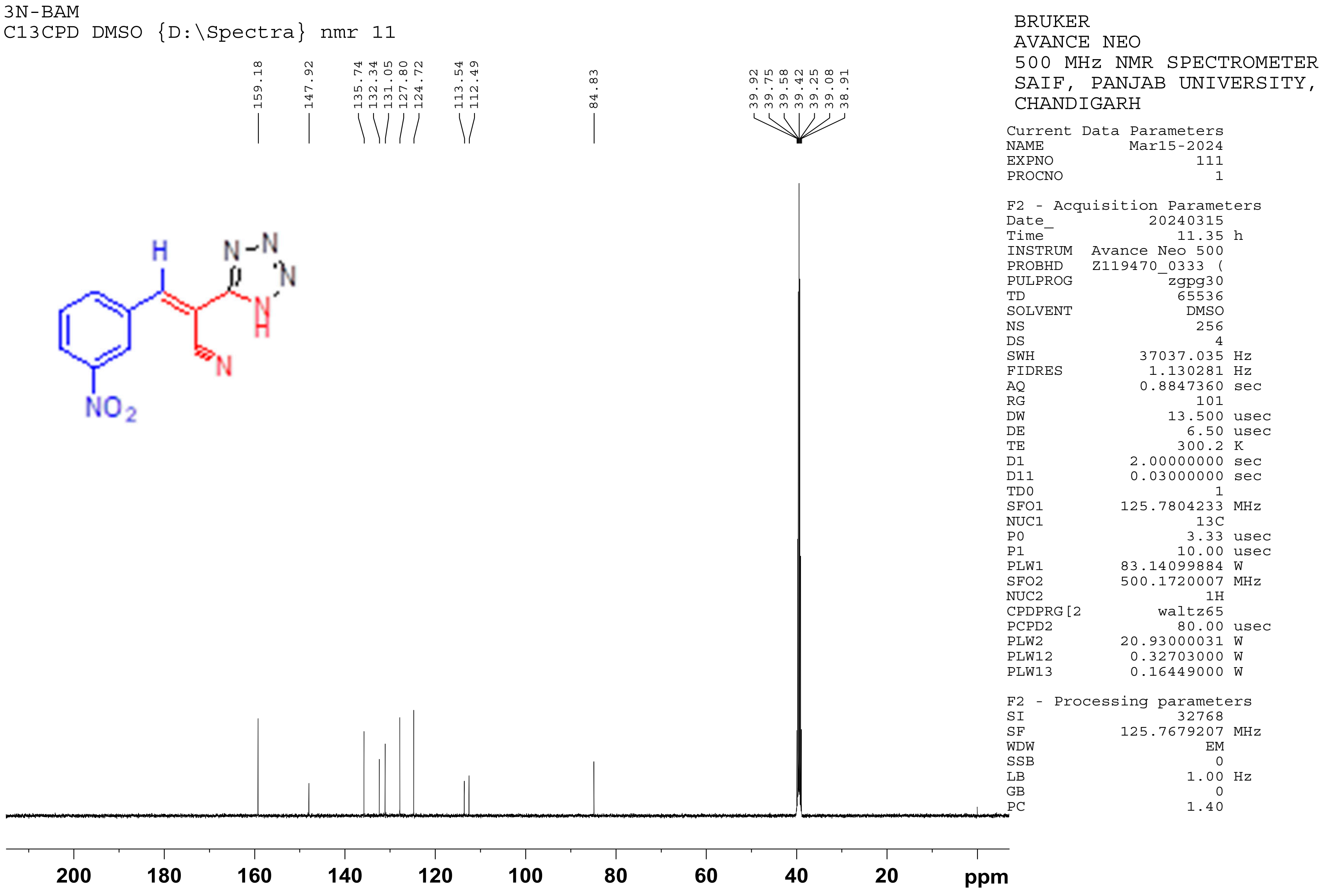

Figure S10: ^{13}C NMR spectra of (Z)-3-(3-nitrophenyl)-2-(1H-tetrazol-5-yl)acrylonitrile **(4b)**

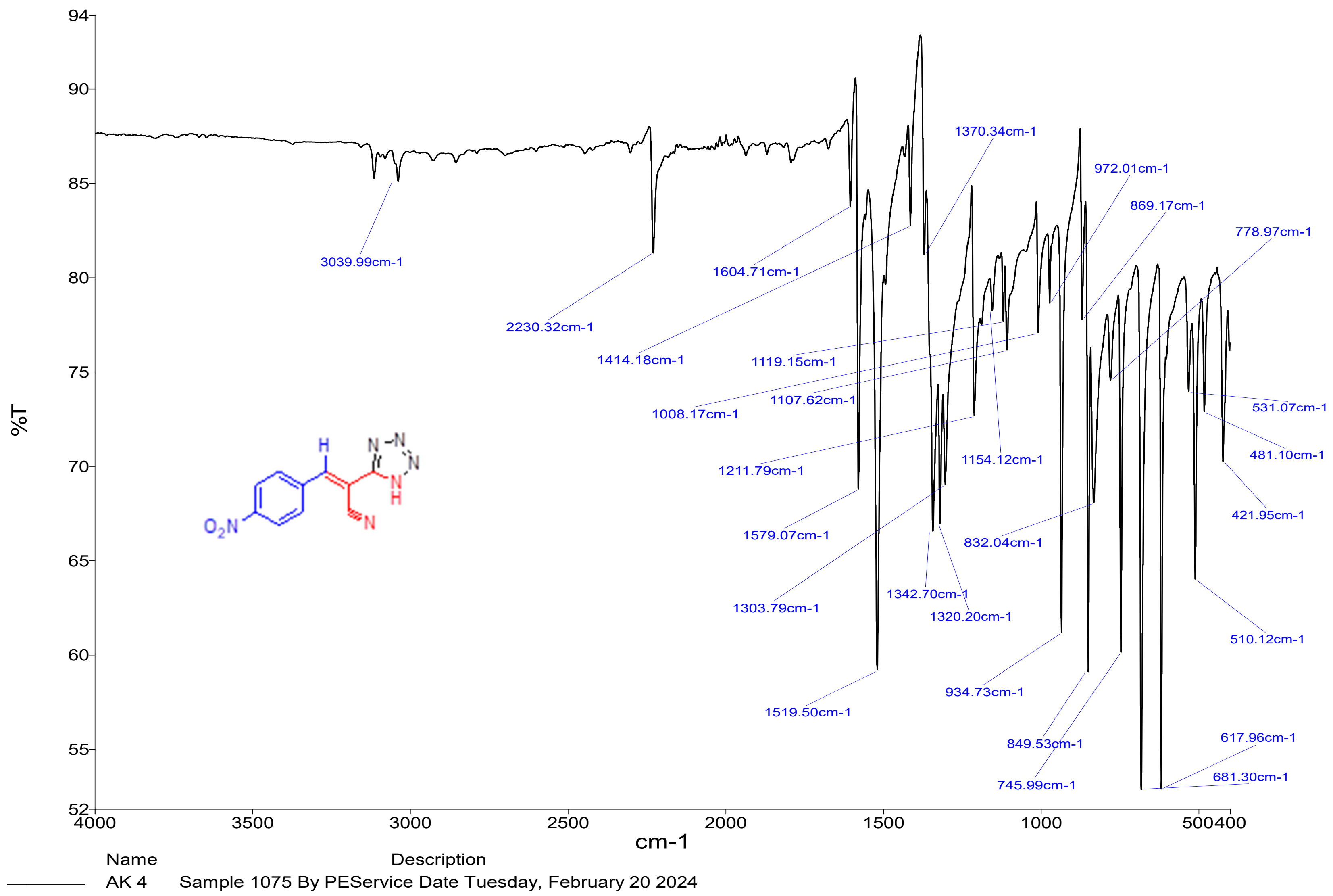

Figure S11: FT-IR spectra of (Z)-3-(4-nitrophenyl)-2-(1H-tetrazol-5-yl)acrylonitrile **(4c)**

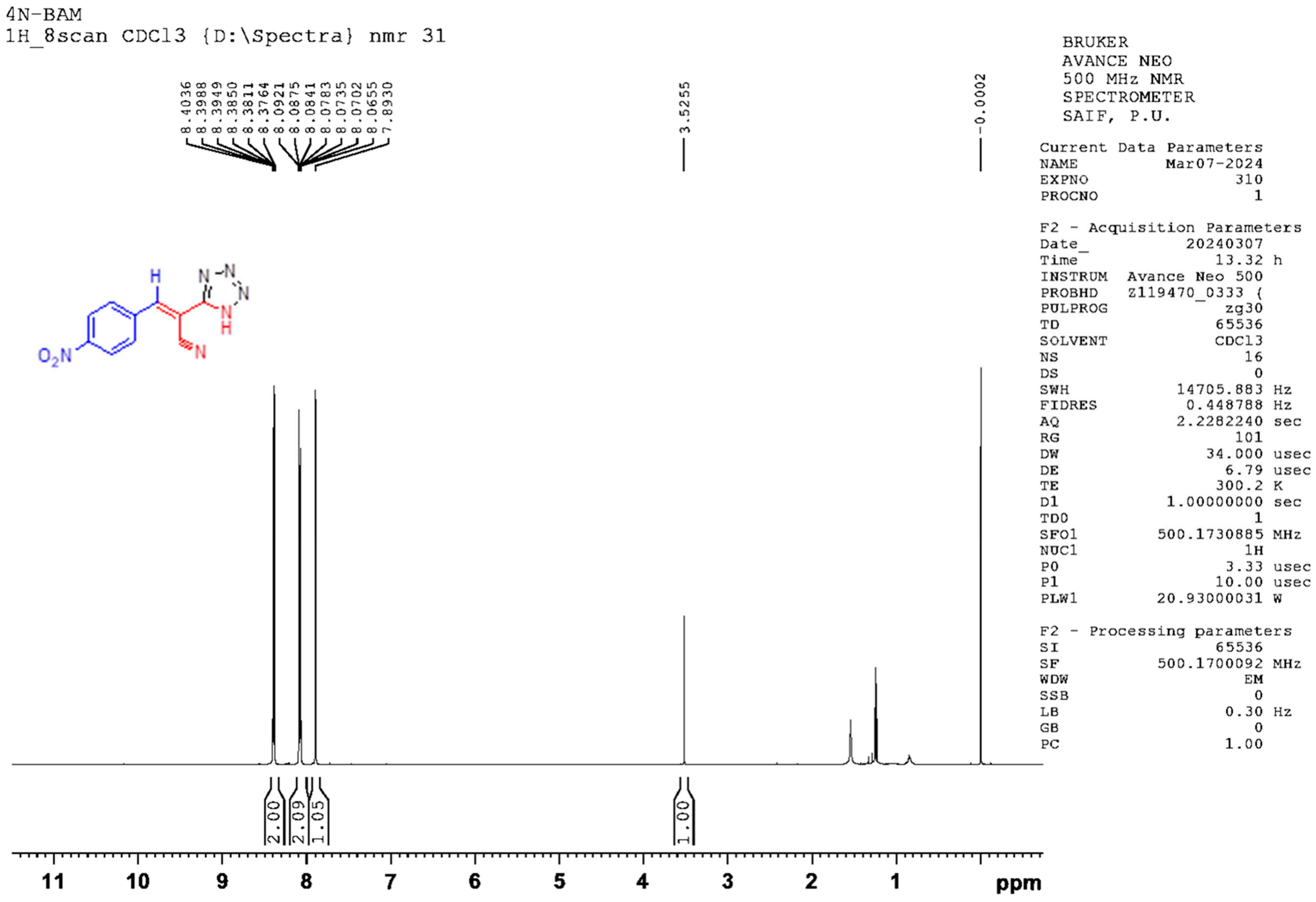

Figure S12: ^{1}H-NMR spectra of (Z)-3-(4-nitrophenyl)-2-(1H-tetrazol-5-yl)acrylonitrile **(4c)**

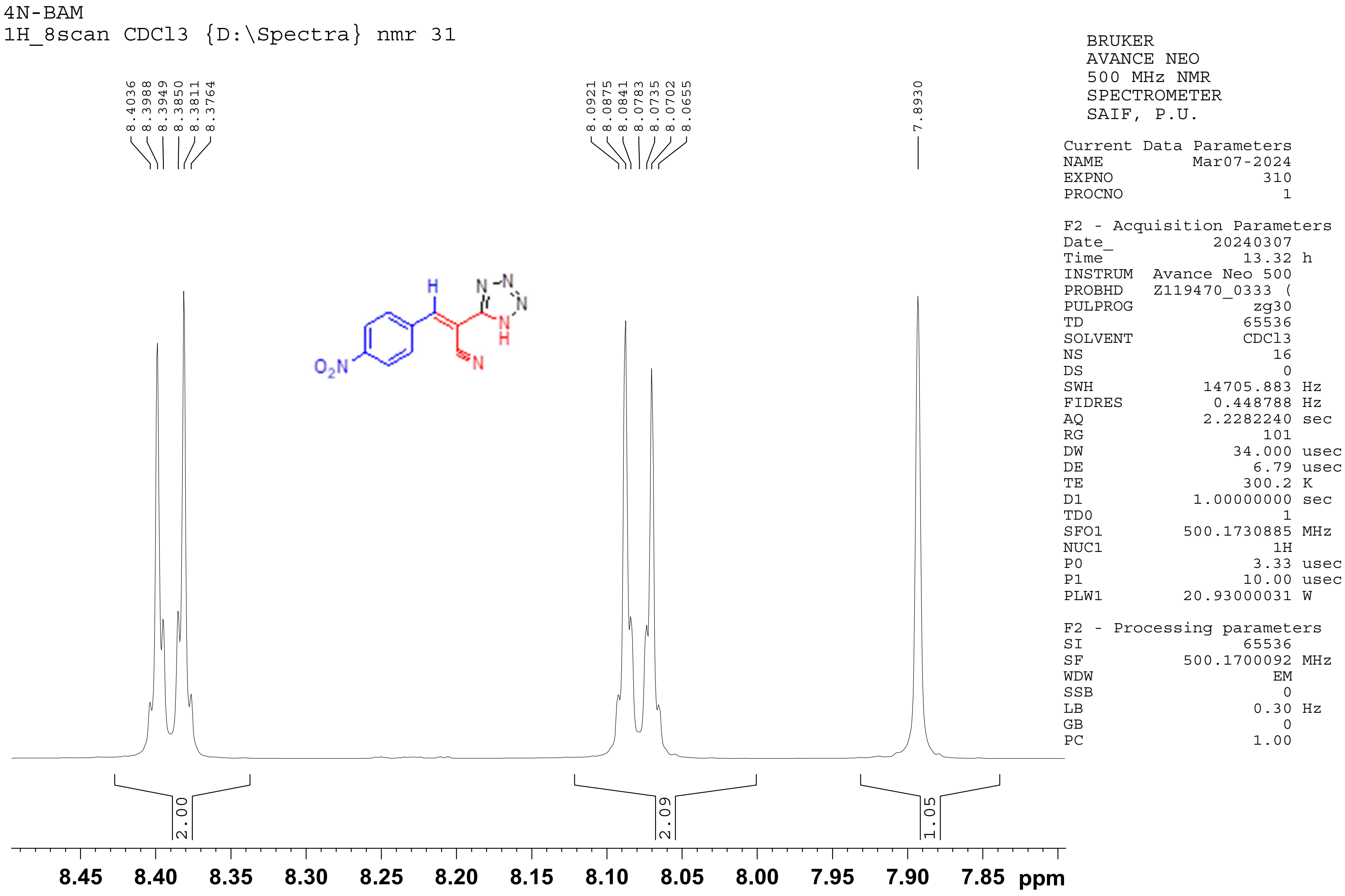

Figure S13: ^{1}H-NMR expanded spectra of (Z)-3-(4-nitrophenyl)-2-(1H-tetrazol-5-yl)acrylonitrile **(4c)**

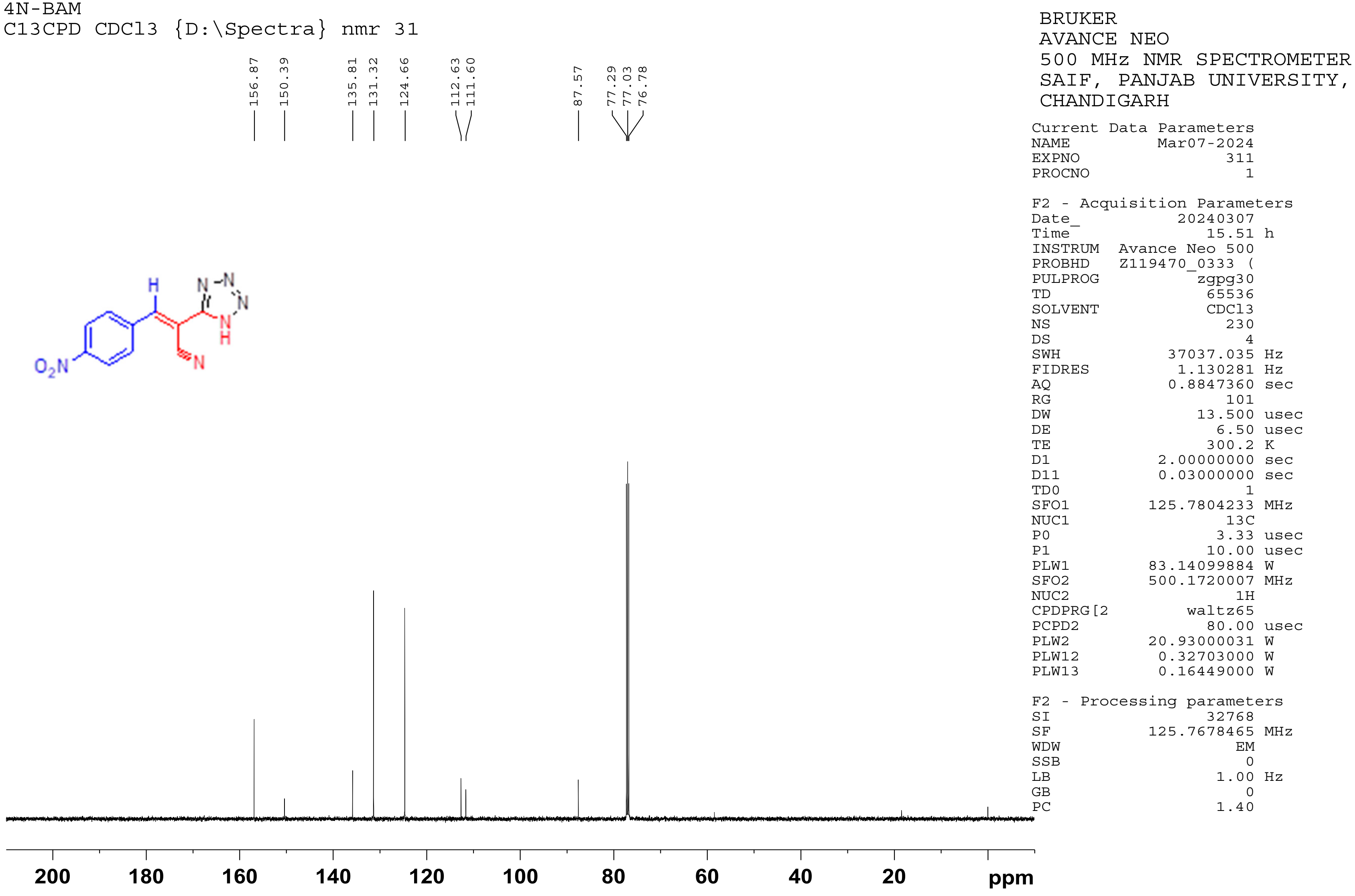

Figure S14: ^{13}C-NMR spectra of (Z)-3-(4-nitrophenyl)-2-(1H-tetrazol-5-yl)acrylonitrile **(4c)**

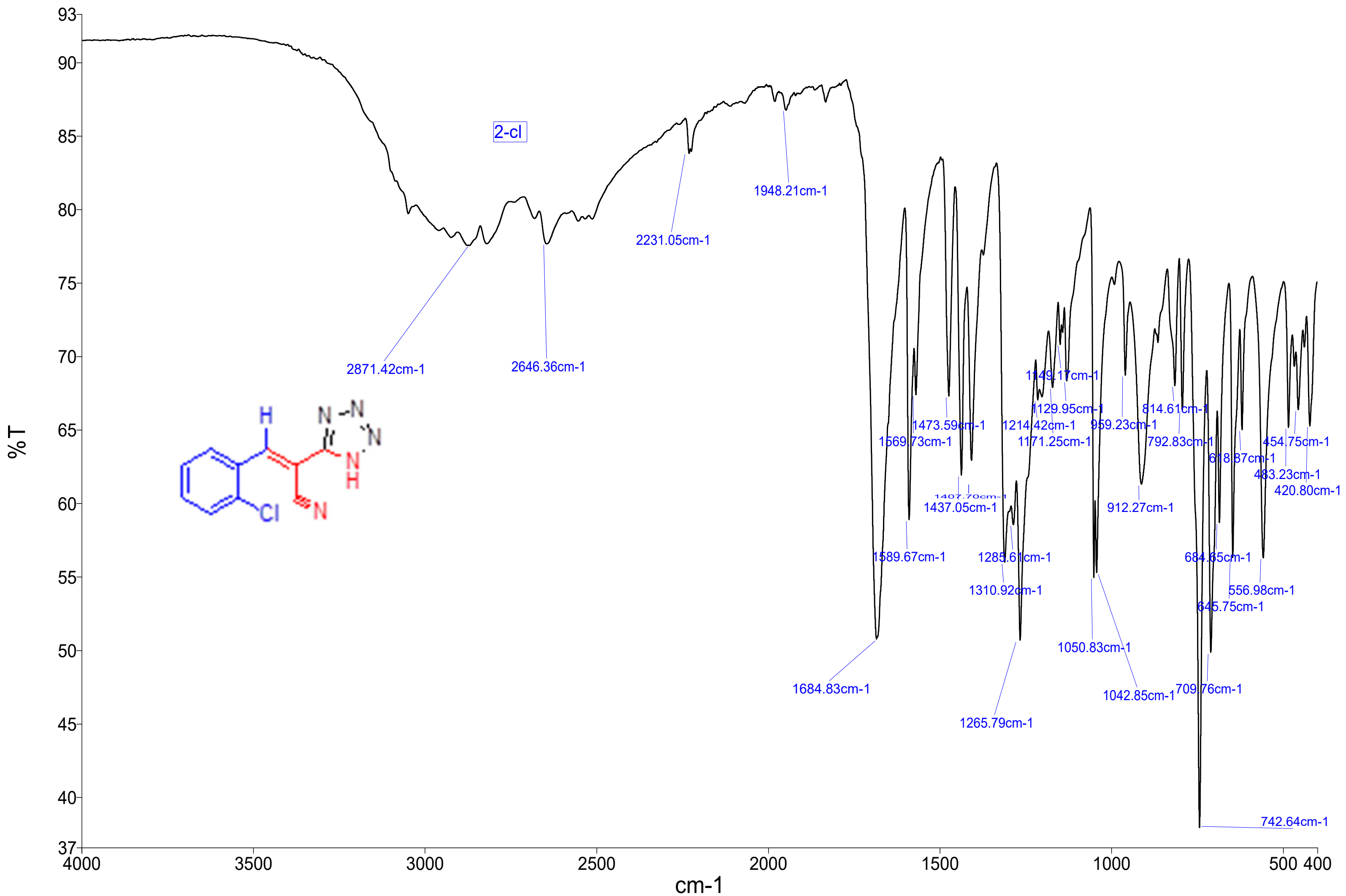

Figure S15: FT-IR spectra of (Z)-3-(2-chlorophenyl)-2-(1H-tetrazol-5-yl)acrylonitrile **(4d)**

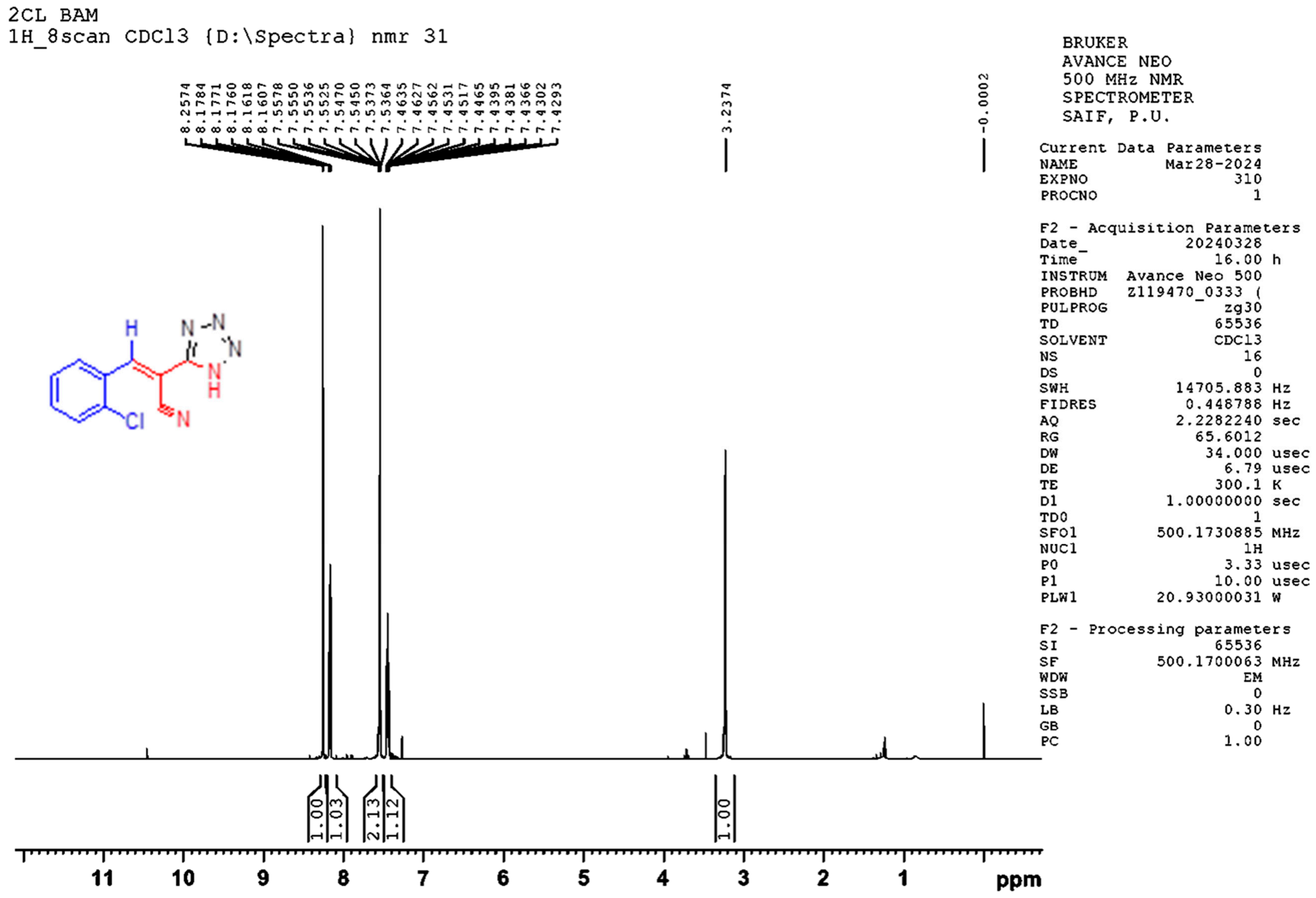

Figure S16: ^{1}H NMR spectra of (Z)-3-(2-chlorophenyl)-2-(1H-tetrazol-5-yl)acrylonitrile **(4d)**

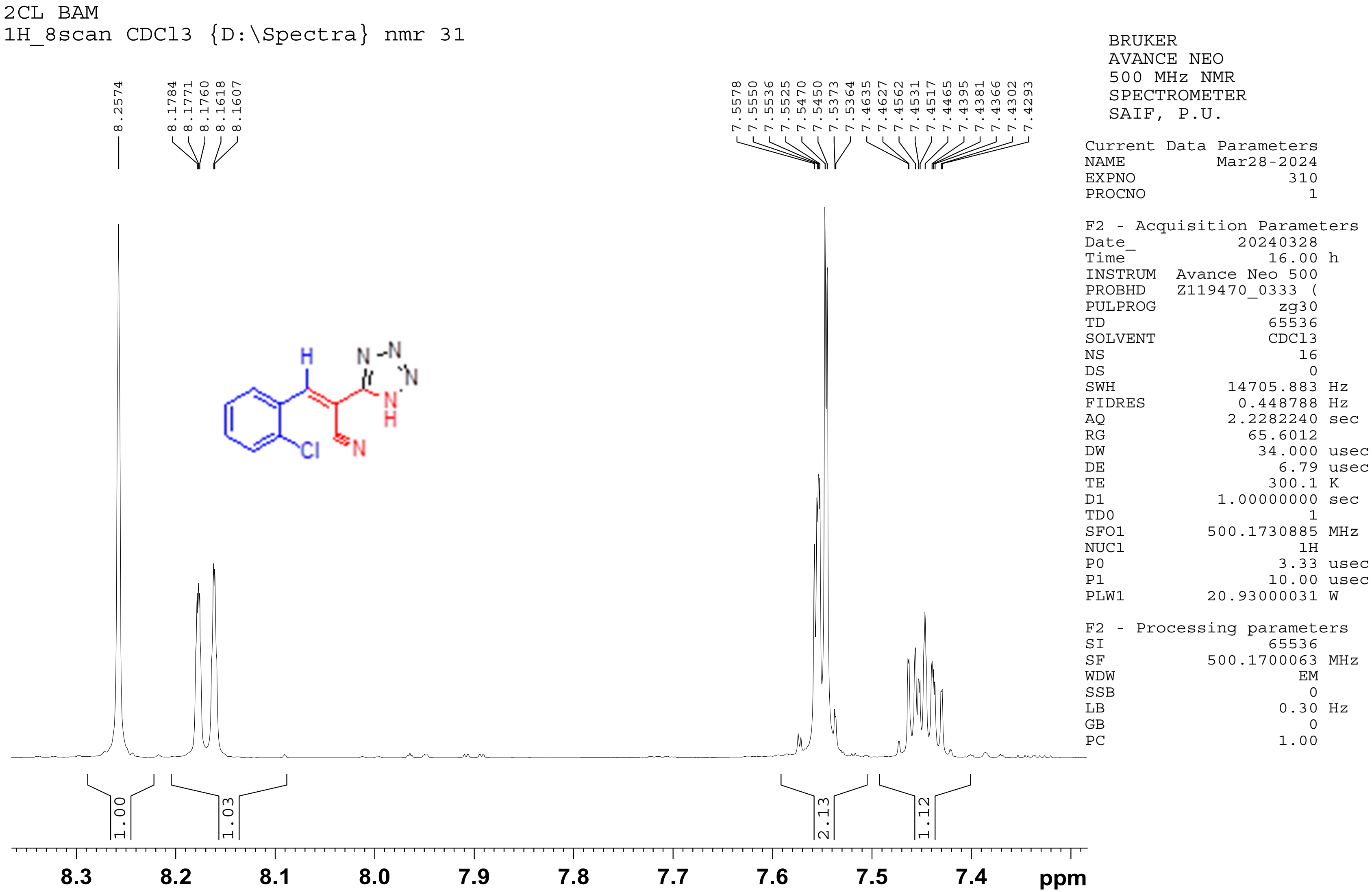

Figure S17: ^{1}H NMR expanded spectra of (Z)-3-(2-chlorophenyl)-2-(1H-tetrazol-5-yl)acrylonitrile **(4d)**

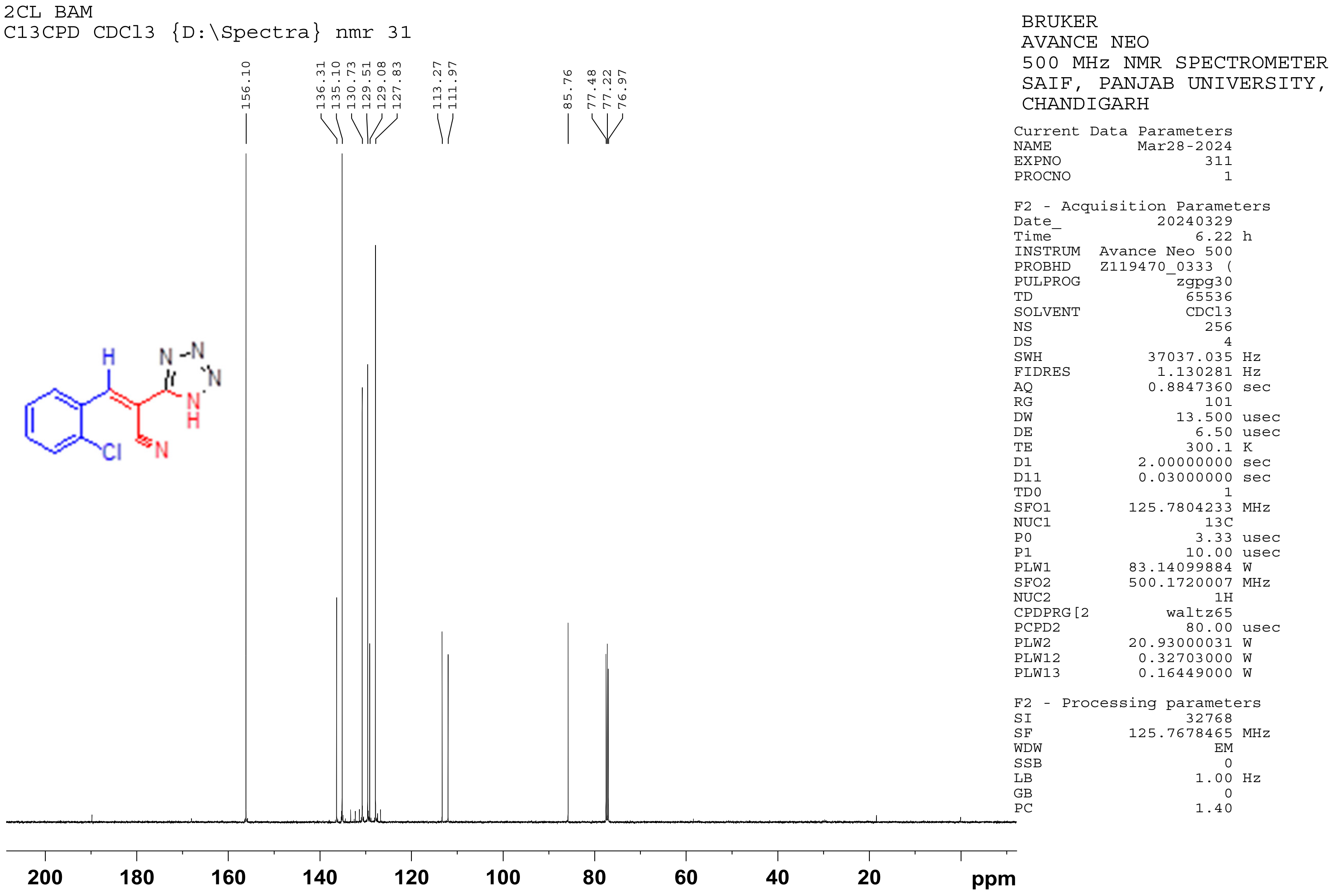

Figure S18: ^{13}C NMR spectra of (Z)-3-(2-chlorophenyl)-2-(1H-tetrazol-5-yl)acrylonitrile **(4d)**

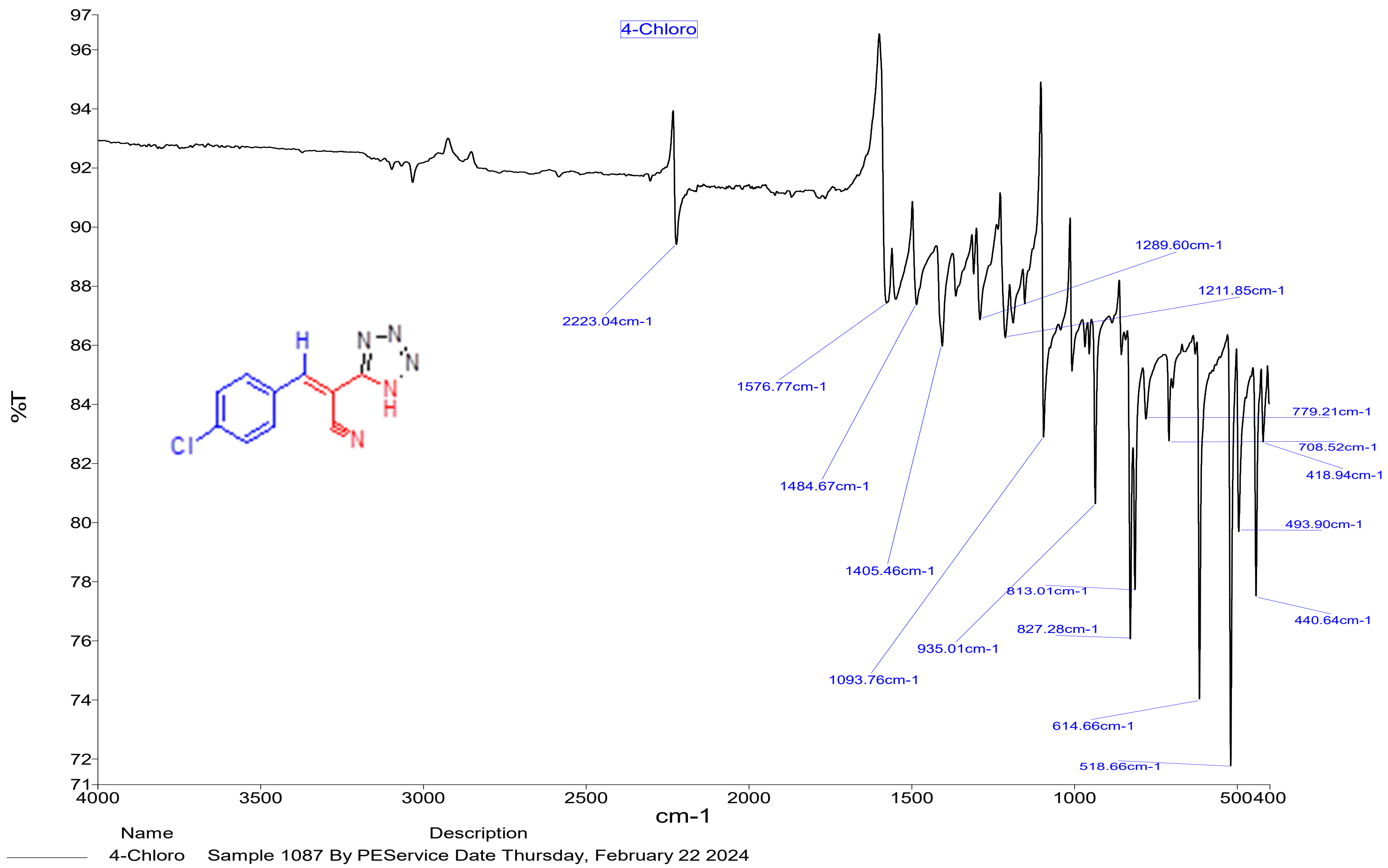

Figure S19: FT-IR spectra of (Z)-3-(4-chlorophenyl)-2-(1H-tetrazol-5-yl)acrylonitrile **(4e)**

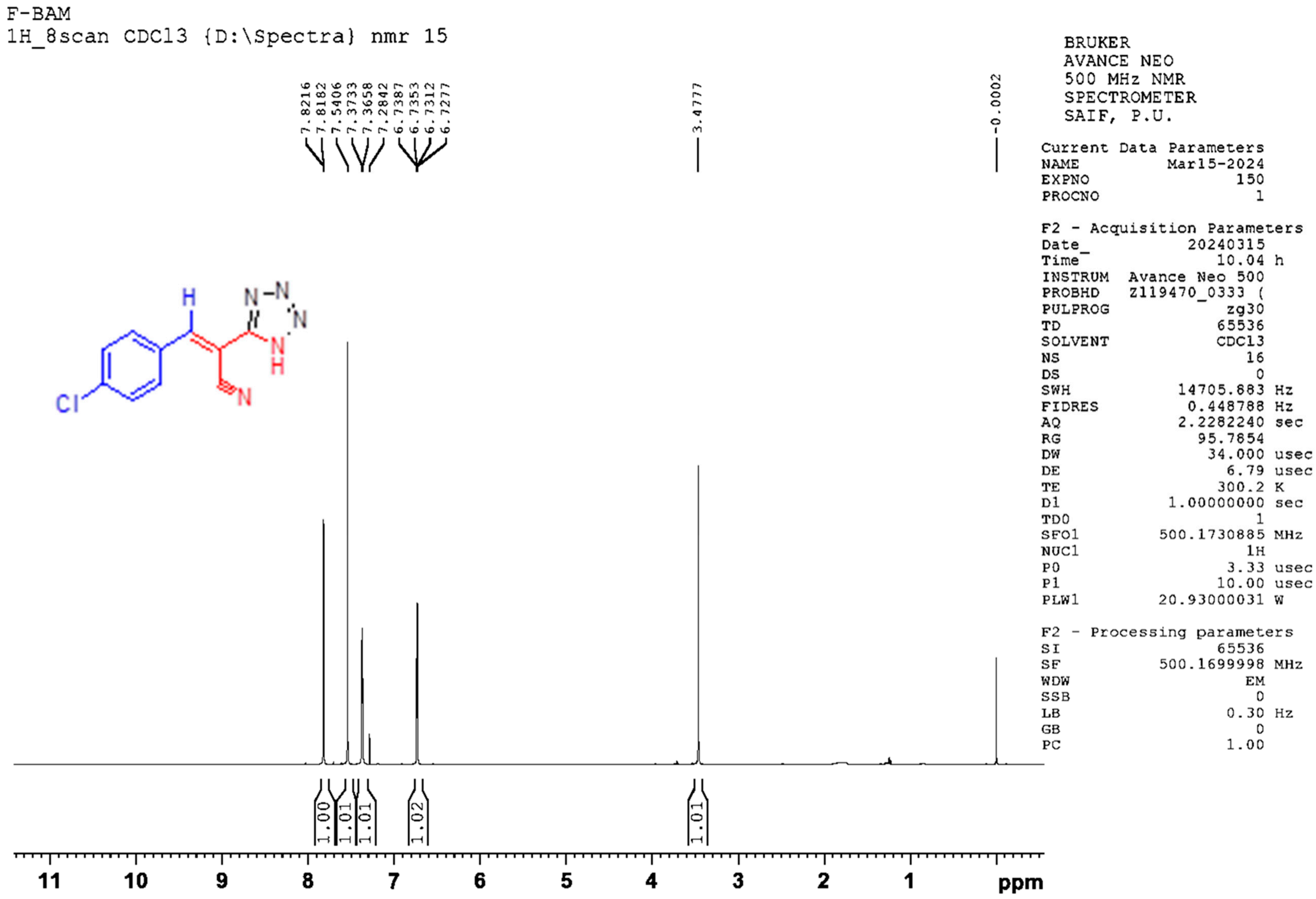

Figure S20: ^{1}H NMR spectra of (Z)-3-(4-chlorophenyl)-2-(1H-tetrazol-5-yl)acrylonitrile **(4e)**

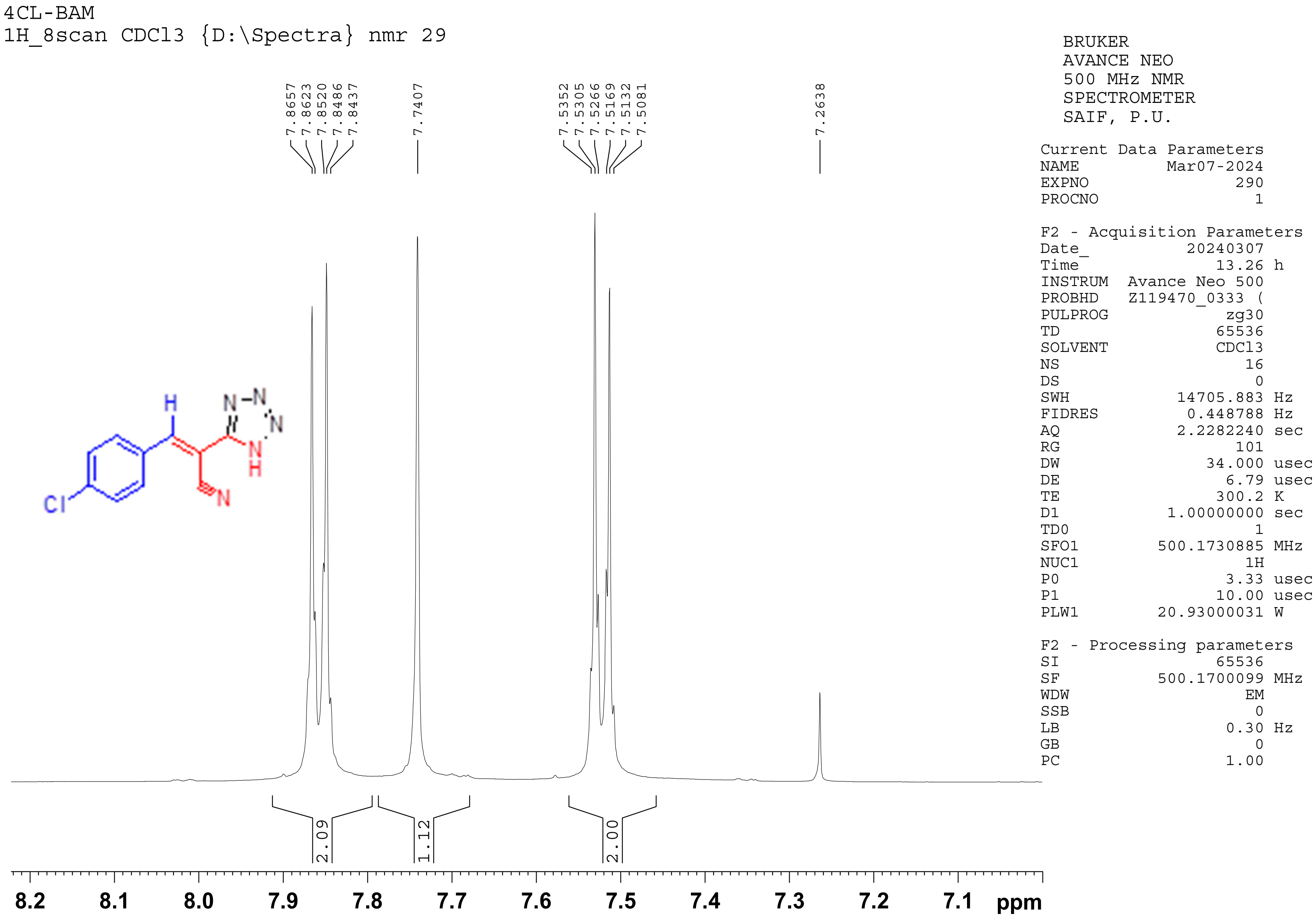

Figure S21: ^{1}H NMR expanded spectra of (Z)-3-(4-chlorophenyl)-2-(1H-tetrazol-5-yl)acrylonitrile **(4e)**

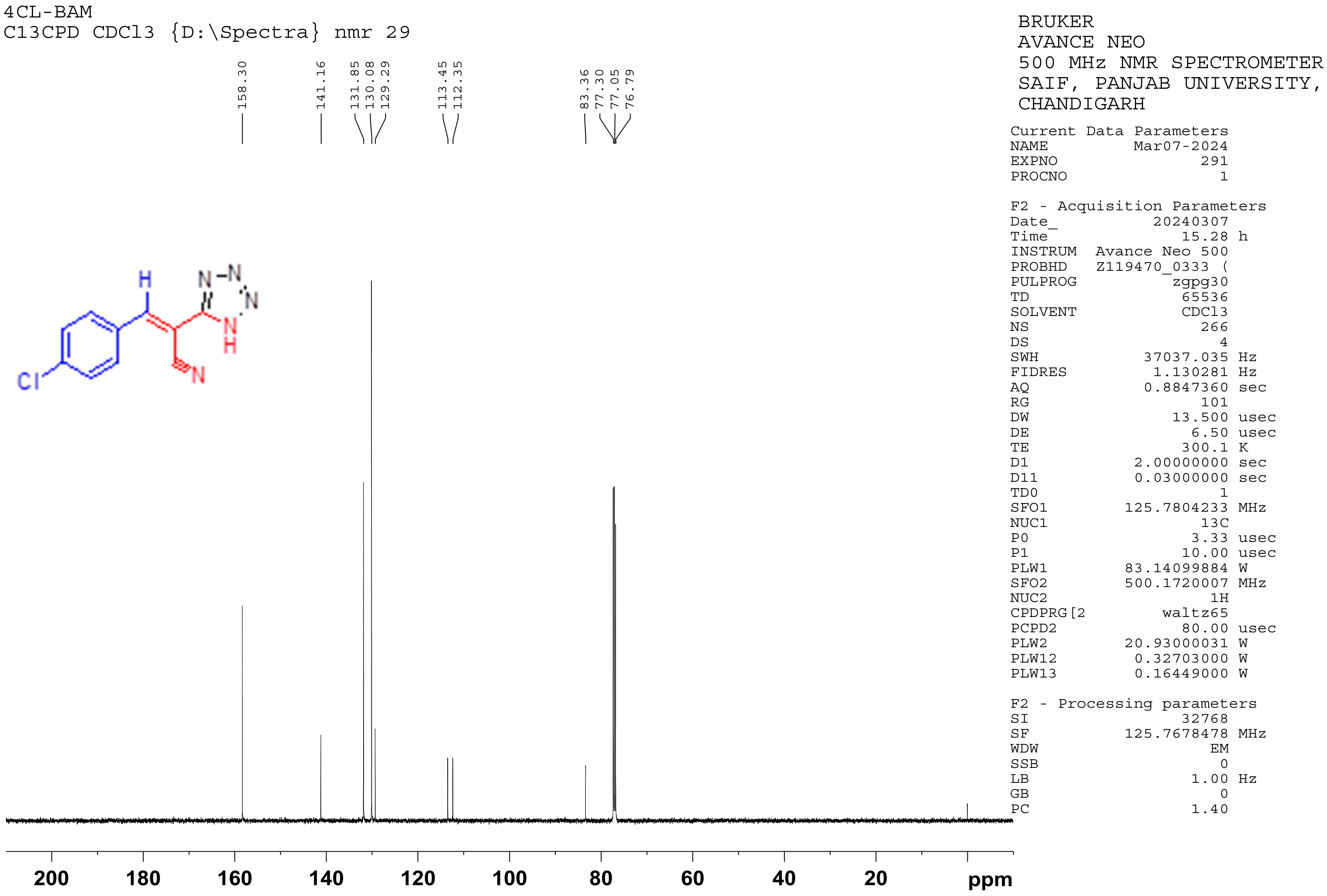

Figure S22: ^{13}C NMR spectra of (Z)-3-(4-chlorophenyl)-2-(1H-tetrazol-5-yl)acrylonitrile **(4e)**

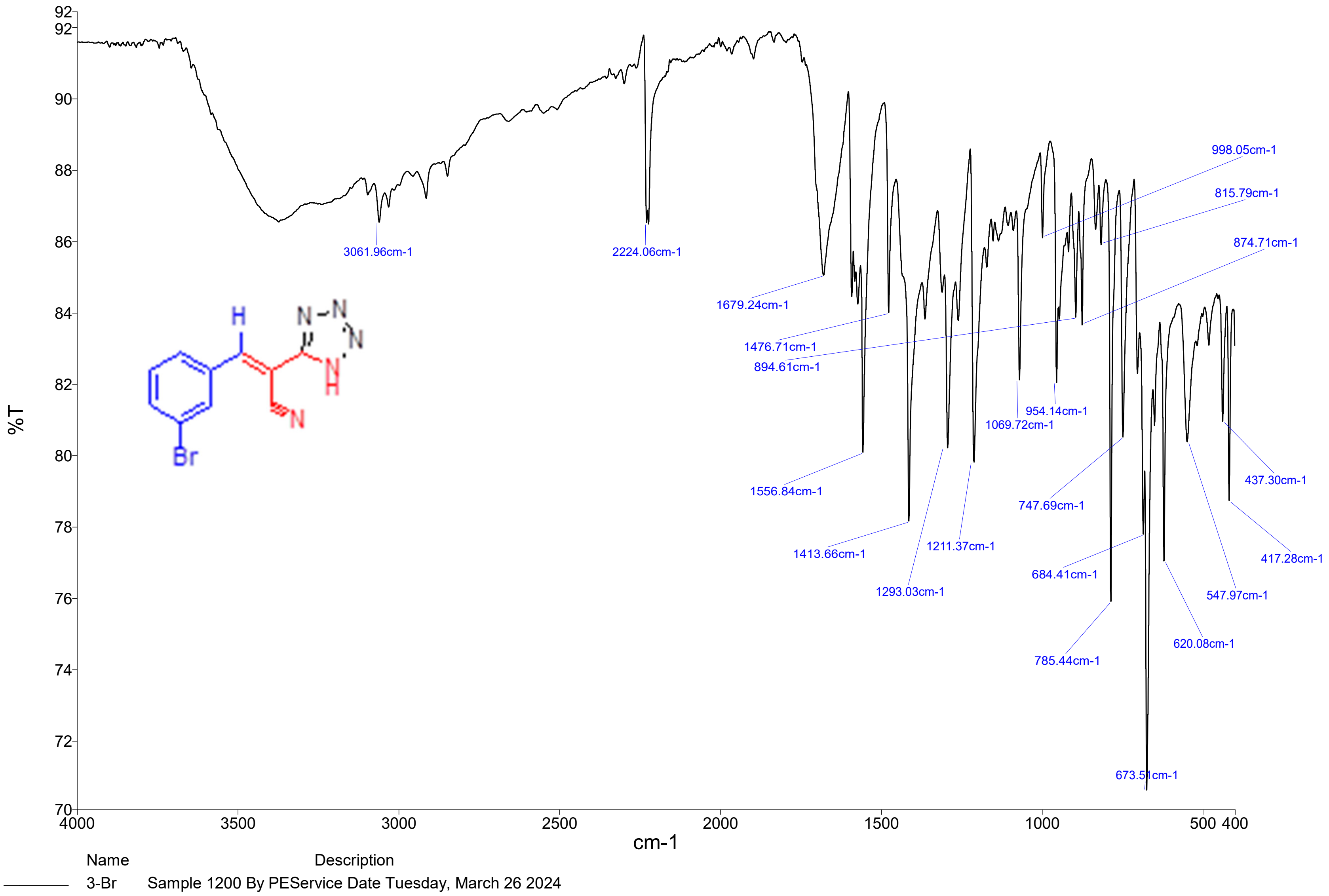

Figure S23: FT-IR spectra of (Z)-3-(3-bromophenyl)-2-(1H-tetrazol-5-yl)acrylonitrile **(4f)**

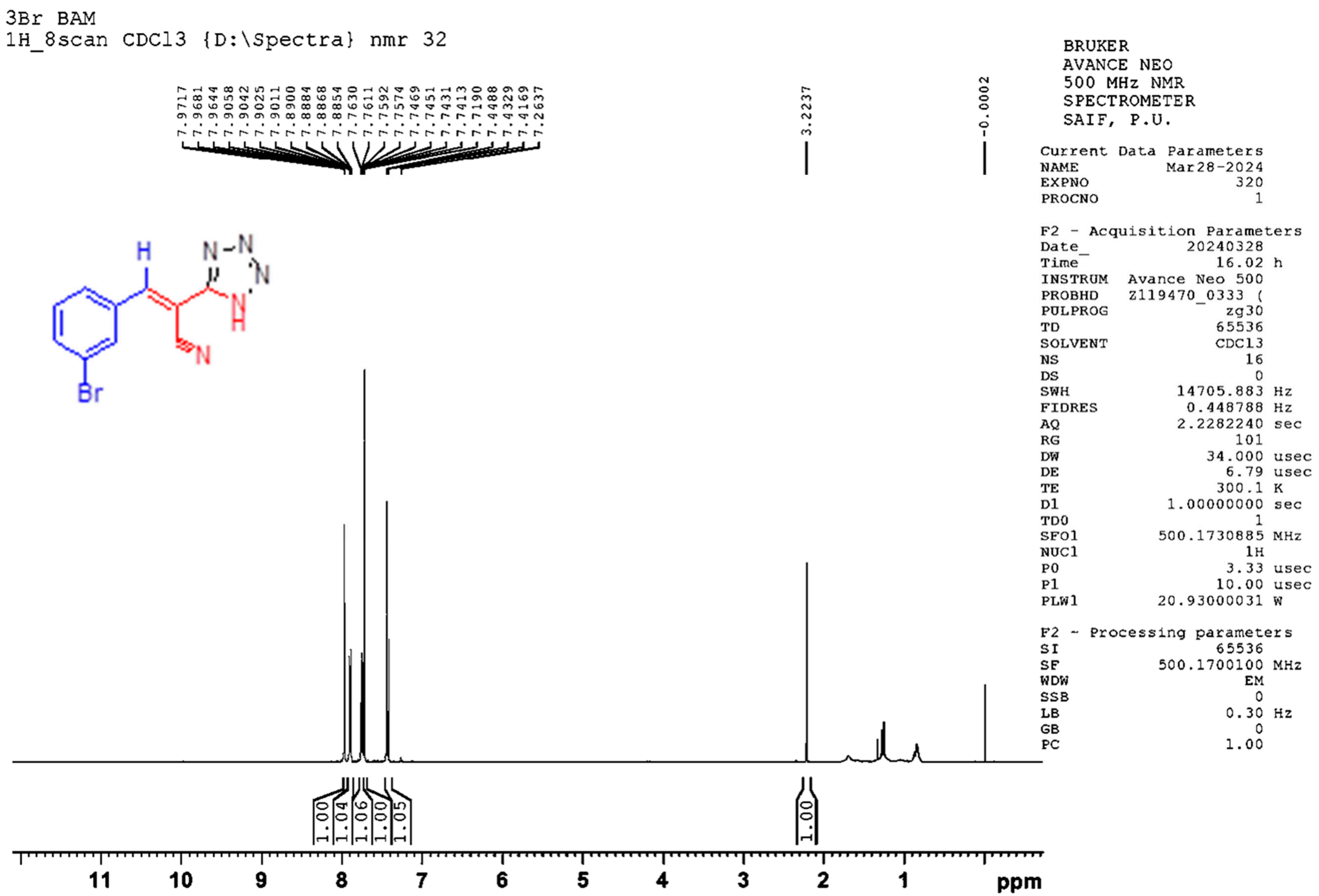

Figure S24: ^{1}H NMR spectra of (Z)-3-(3-bromophenyl)-2-(1H-tetrazol-5-yl)acrylonitrile **(4f)**

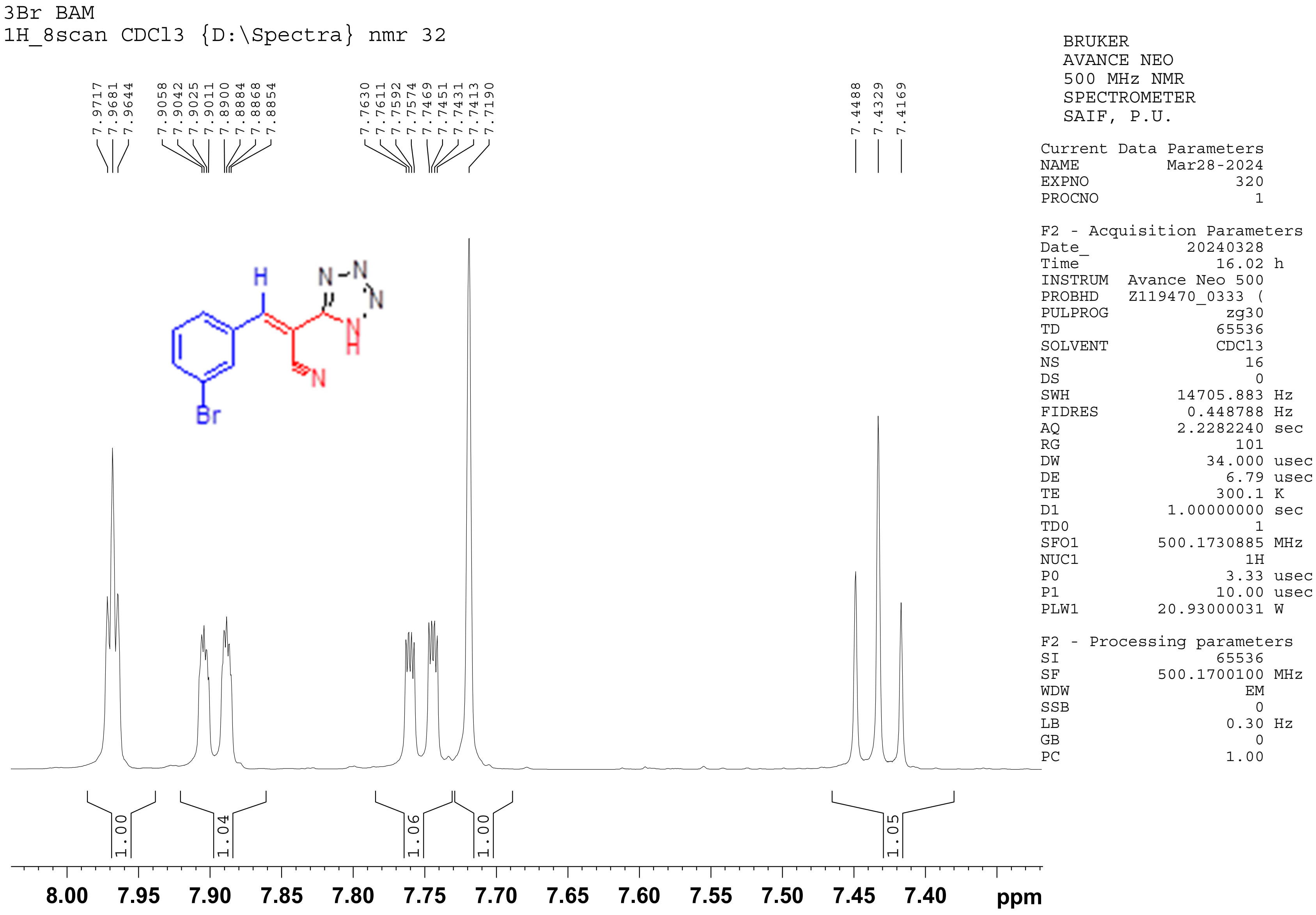

Figure S25: ^{1}H NMR expanded spectra of (Z)-3-(3-bromophenyl)-2-(1H-tetrazol-5-yl)acrylonitrile **(4f)**

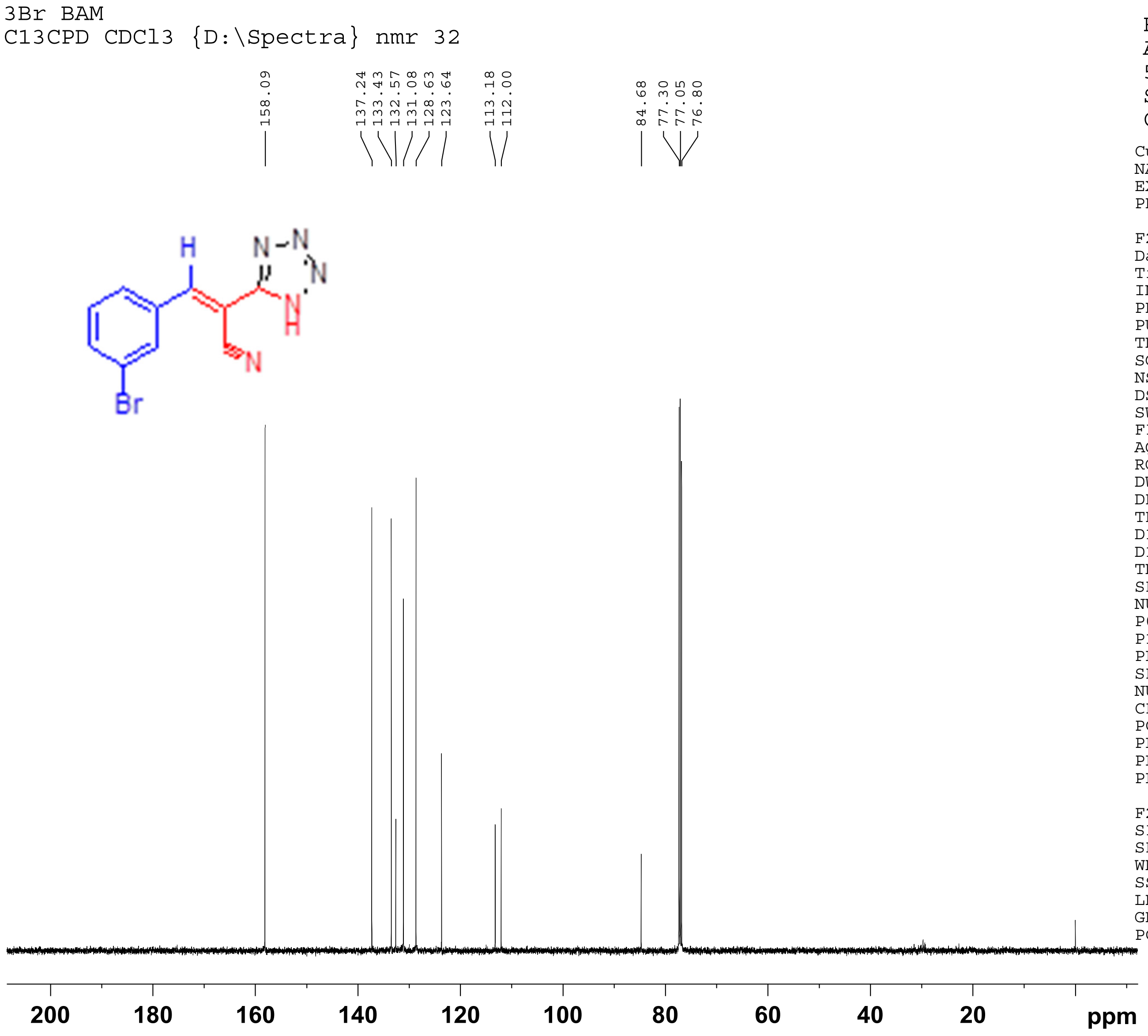

BRUKER
AVANCE NEO
500 MHz NMR SPECTROMETER
SAIF, PANJAB UNIVERSITY,
CHANDIGARH

```
Current Data Parameters
NAME            Mar28-2024
EXPNO                  321
PROCNO                   1

F2 - Acquisition Parameters
Date_             20240329
Time                  6.37 h
INSTRUM     Avance Neo 500
PROBHD      Z119470_0333 (
PULPROG            zgpg30
TD                  65536
SOLVENT             CDCl3
NS                    256
DS                      4
SWH             37037.035 Hz
FIDRES           1.130281 Hz
AQ              0.8847360 sec
RG                    101
DW                 13.500 usec
DE                   6.50 usec
TE                  300.2 K
D1             2.00000000 sec
D11            0.03000000 sec
TD0                     1
SFO1          125.7804233 MHz
NUC1                  13C
P0                   3.33 usec
P1                  10.00 usec
PLW1          83.14099884 W
SFO2          500.1720007 MHz
NUC2                   1H
CPDPRG[2          waltz65
PCPD2               80.00 usec
PLW2          20.93000031 W
PLW12          0.32703000 W
PLW13          0.16449000 W

F2 - Processing parameters
SI                  32768
SF            125.7678489 MHz
WDW                    EM
SSB                     0
LB                   1.00 Hz
GB                      0
PC                   1.40
```

Figure S26: ^{13}C NMR spectra of (Z)-3-(3-bromophenyl)-2-(1H-tetrazol-5-yl)acrylonitrile **(4f)**

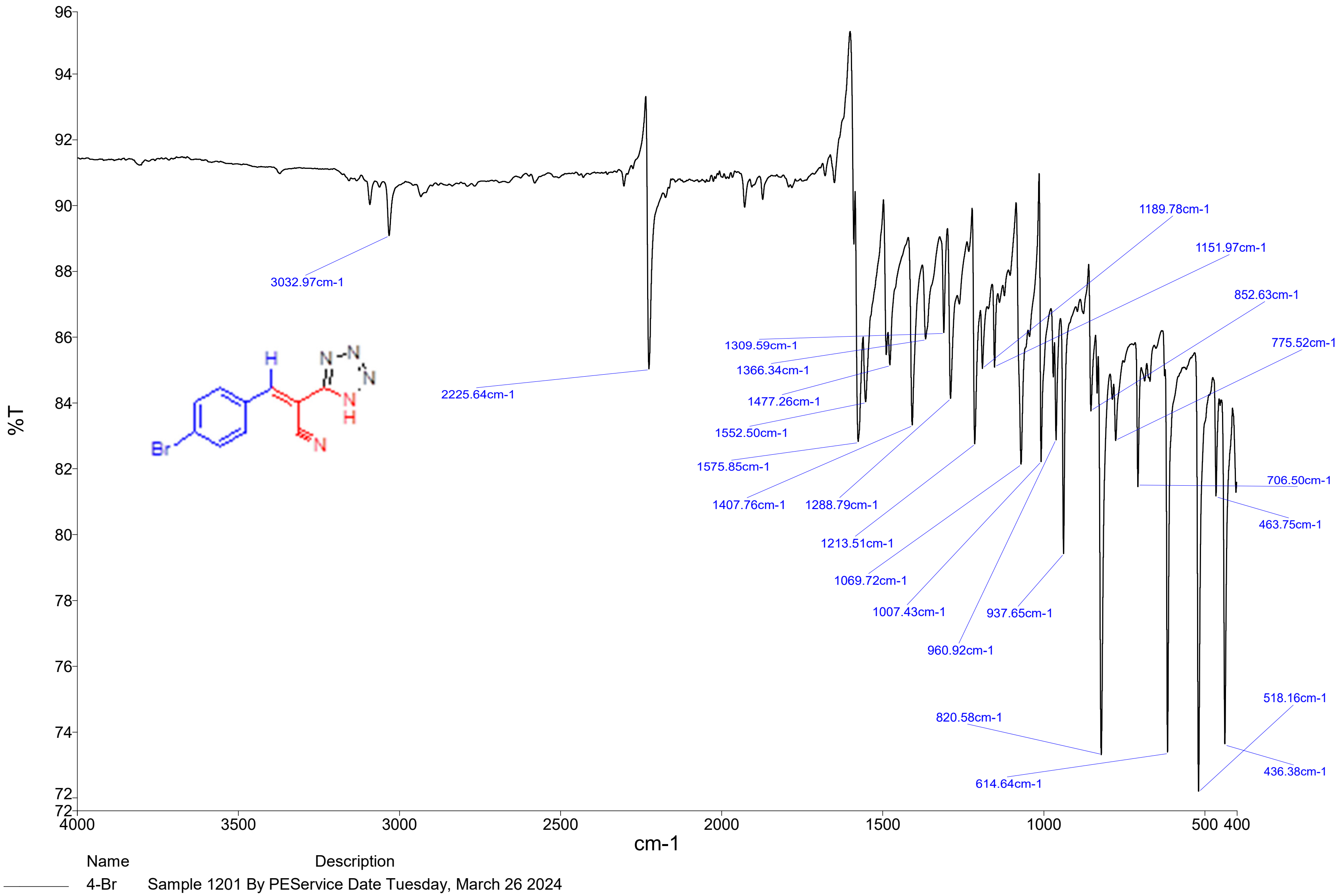

Figure S27: FT-IR spectra of (Z)-3-(4-bromophenyl)-2-(1H-tetrazol-5-yl)acrylonitrile **(4g)**

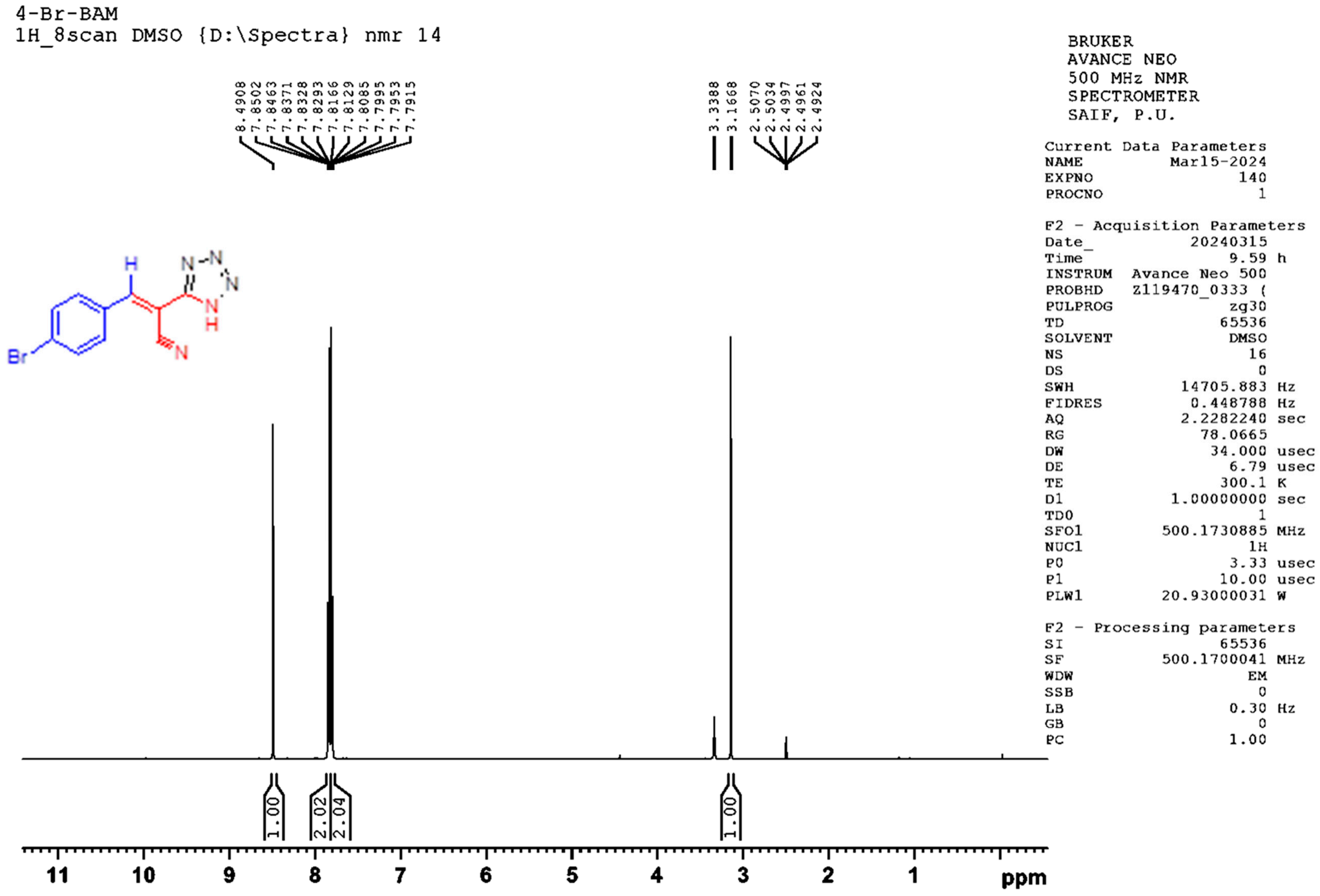

Figure S28: ^{1}H-NMR spectra of (Z)-3-(4-bromophenyl)-2-(1H-tetrazol-5-yl)acrylonitrile **(4g)**

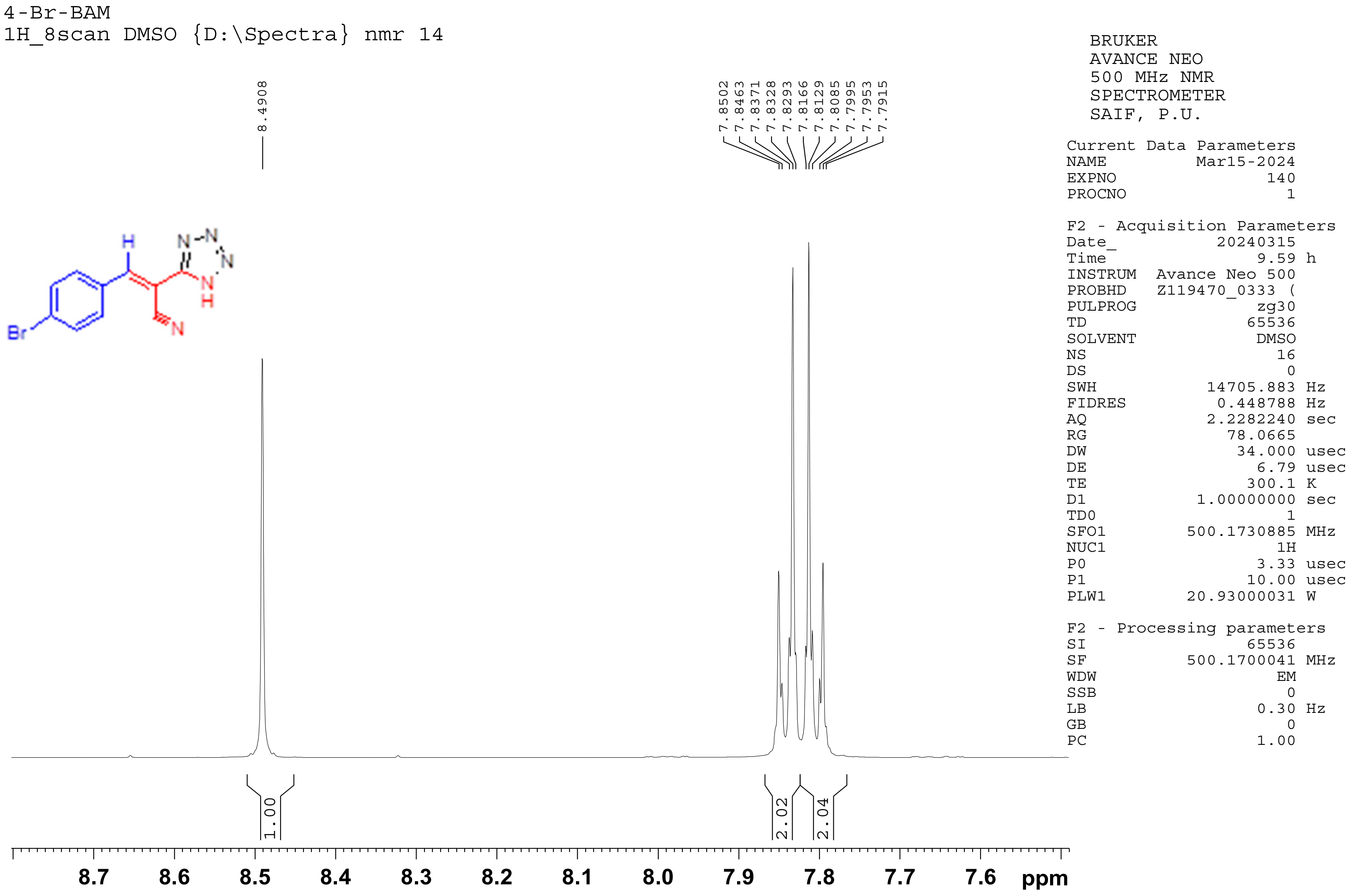

Figure S29: ^{1}H-NMR expanded spectra of (Z)-3-(4-bromophenyl)-2-(1H-tetrazol-5-yl)acrylonitrile **(4g)**

4-Br-BAM
C13CPD DMSO {D:\Spectra} nmr 14

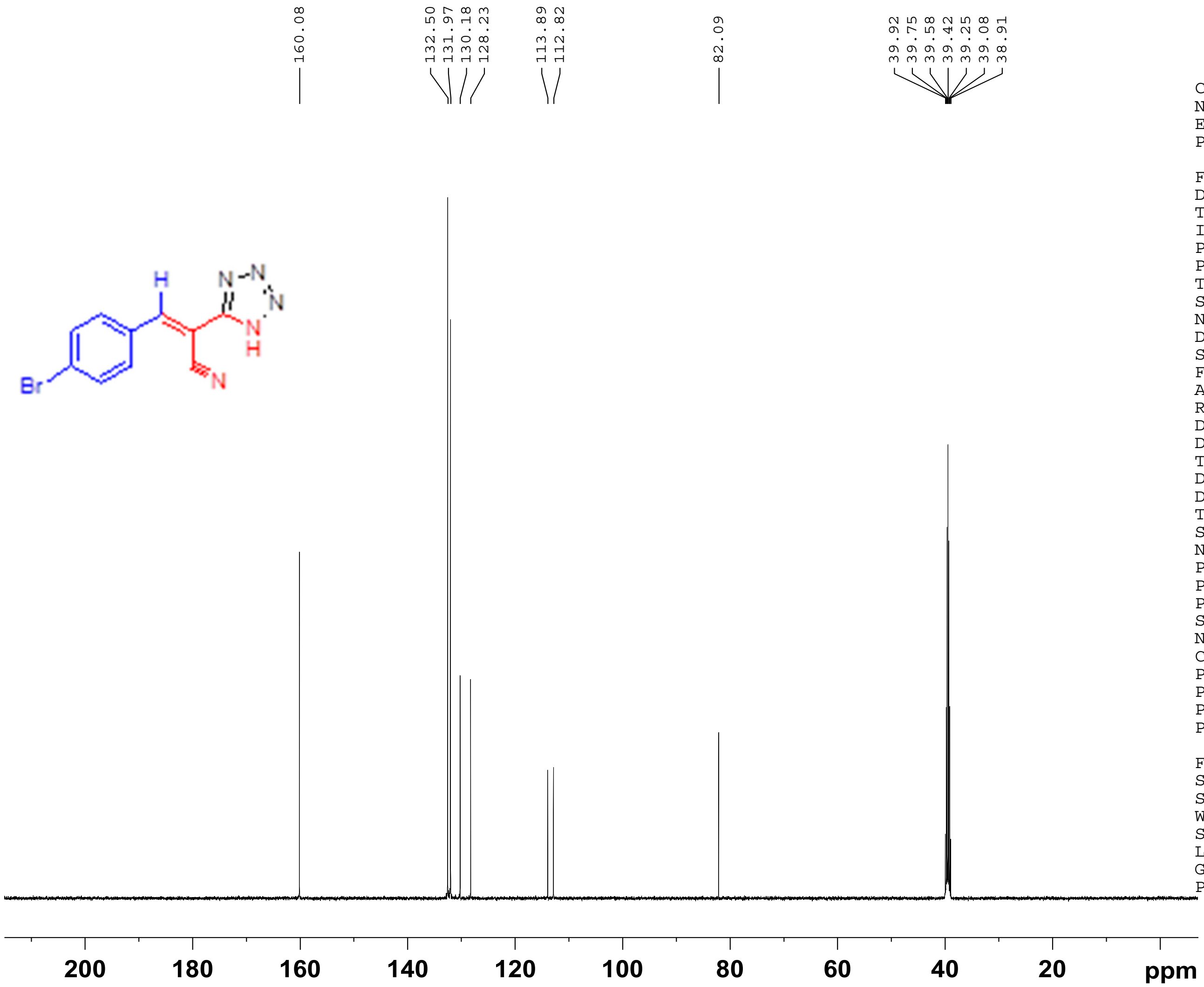

BRUKER
AVANCE NEO
500 MHz NMR SPECTROMETER
SAIF, PANJAB UNIVERSITY,
CHANDIGARH

Current Data Parameters

NAME	Mar15-2024
EXPNO	141
PROCNO	1

F2 - Acquisition Parameters

Date_	20240315
Time	11.59 h
INSTRUM	Avance Neo 500
PROBHD	Z119470_0333 (
PULPROG	zgpg30
TD	65536
SOLVENT	DMSO
NS	120
DS	4
SWH	37037.035 Hz
FIDRES	1.130281 Hz
AQ	0.8847360 sec
RG	101
DW	13.500 usec
DE	6.50 usec
TE	300.2 K
D1	2.00000000 sec
D11	0.03000000 sec
TD0	1
SFO1	125.7804233 MHz
NUC1	13C
P0	3.33 usec
P1	10.00 usec
PLW1	83.14099884 W
SFO2	500.1720007 MHz
NUC2	1H
CPDPRG[2	waltz65
PCPD2	80.00 usec
PLW2	20.93000031 W
PLW12	0.32703000 W
PLW13	0.16449000 W

F2 - Processing parameters

SI	32768
SF	125.7679233 MHz
WDW	EM
SSB	0
LB	1.00 Hz
GB	0
PC	1.40

Figure S30: ^{13}C-NMR spectra of (Z)-3-(4-bromophenyl)-2-(1H-tetrazol-5-yl)acrylonitrile **(4g)**

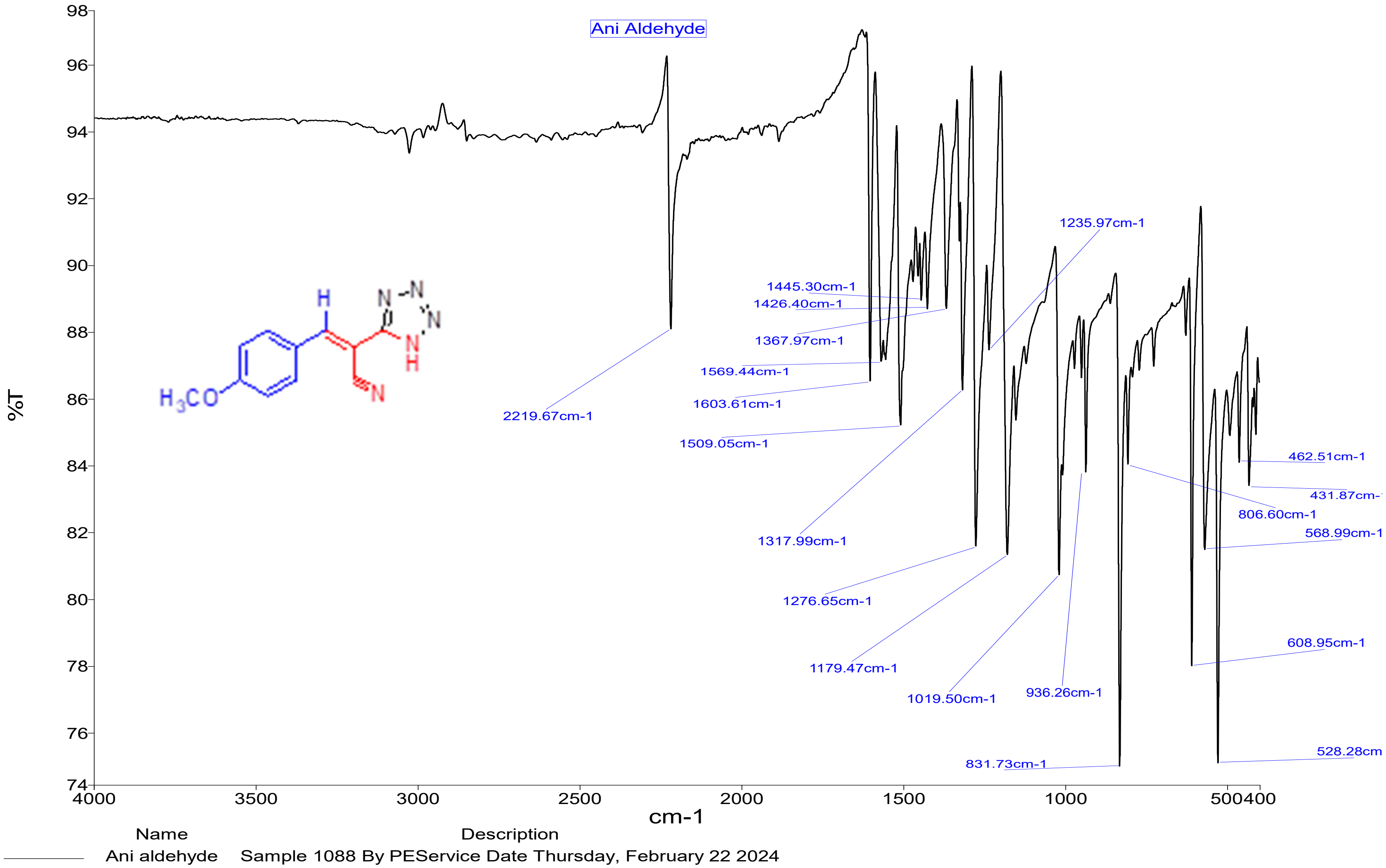

Figure S31: FT-IR spectra of (Z)-3-(4-methoxyphenyl)-2-(1H-tetrazol-5-yl)acrylonitrile **(4h)**

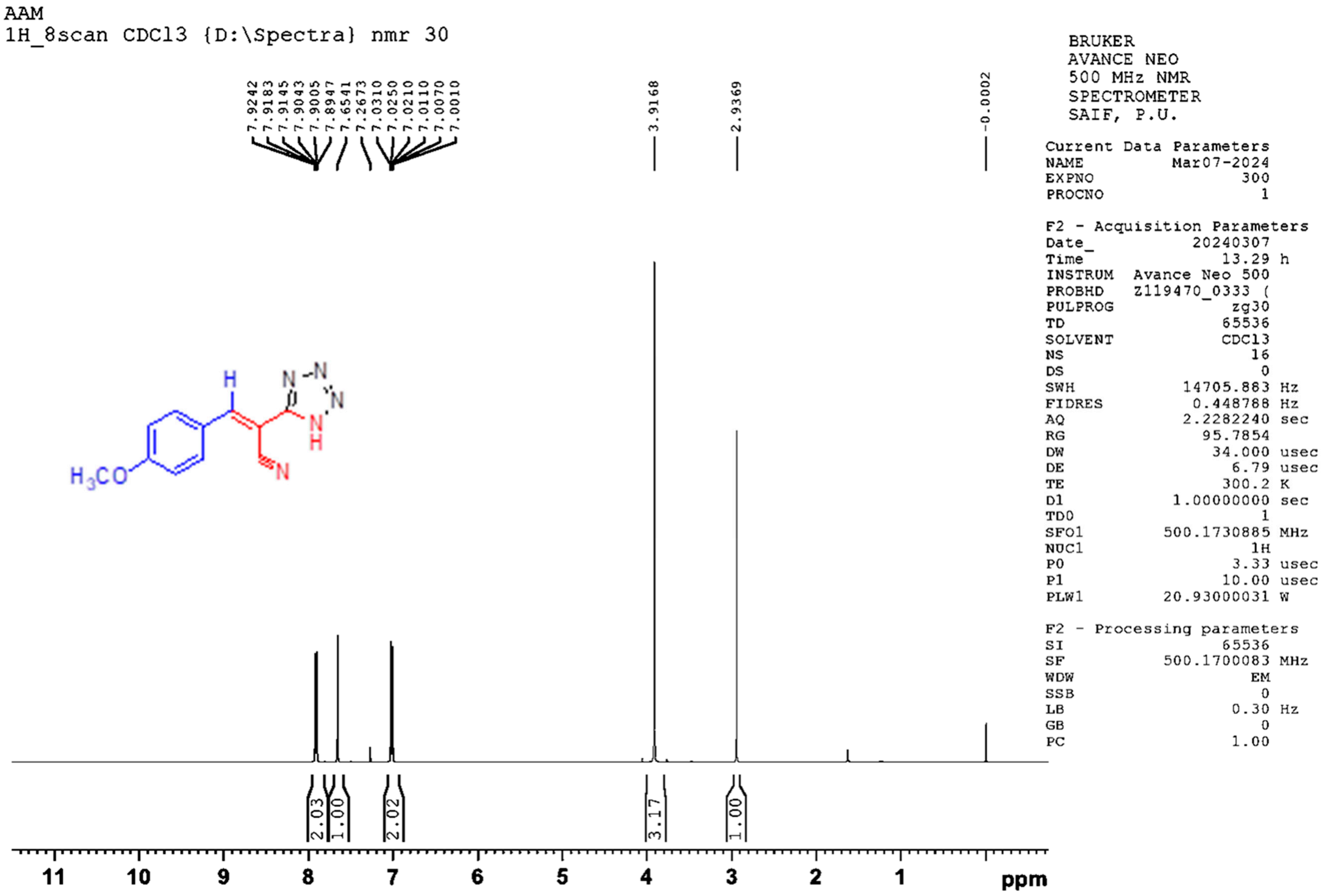

Figure S32: ^{1}H-NMR spectra of (Z)-3-(4-methoxyphenyl)-2-(1H-tetrazol-5-yl)acrylonitrile **(4h)**

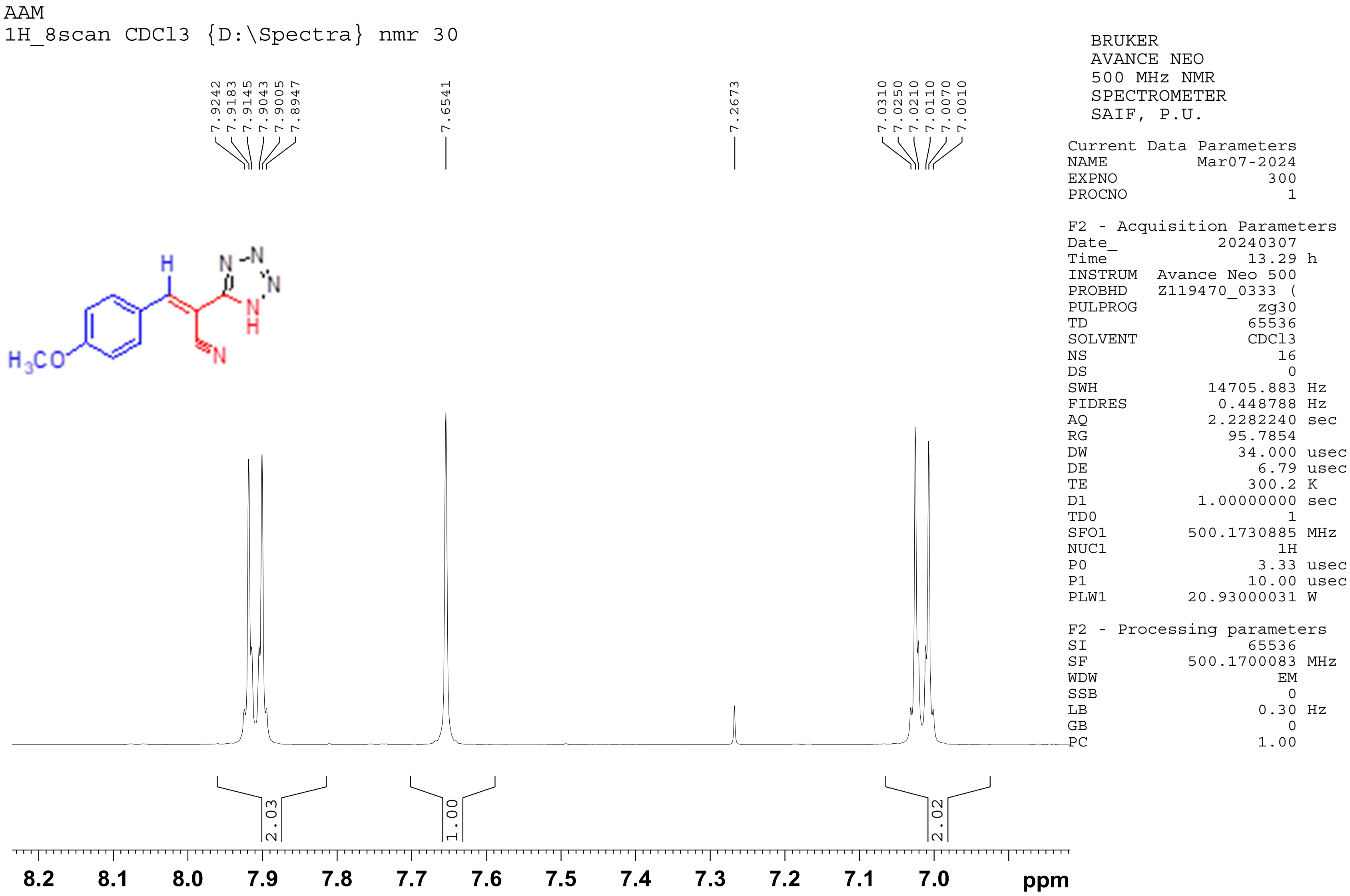

Figure S33: ^{1}H-NMR expanded spectra of (Z)-3-(4-methoxyphenyl)-2-(1H-tetrazol-5-yl)acrylonitrile **(4h)**

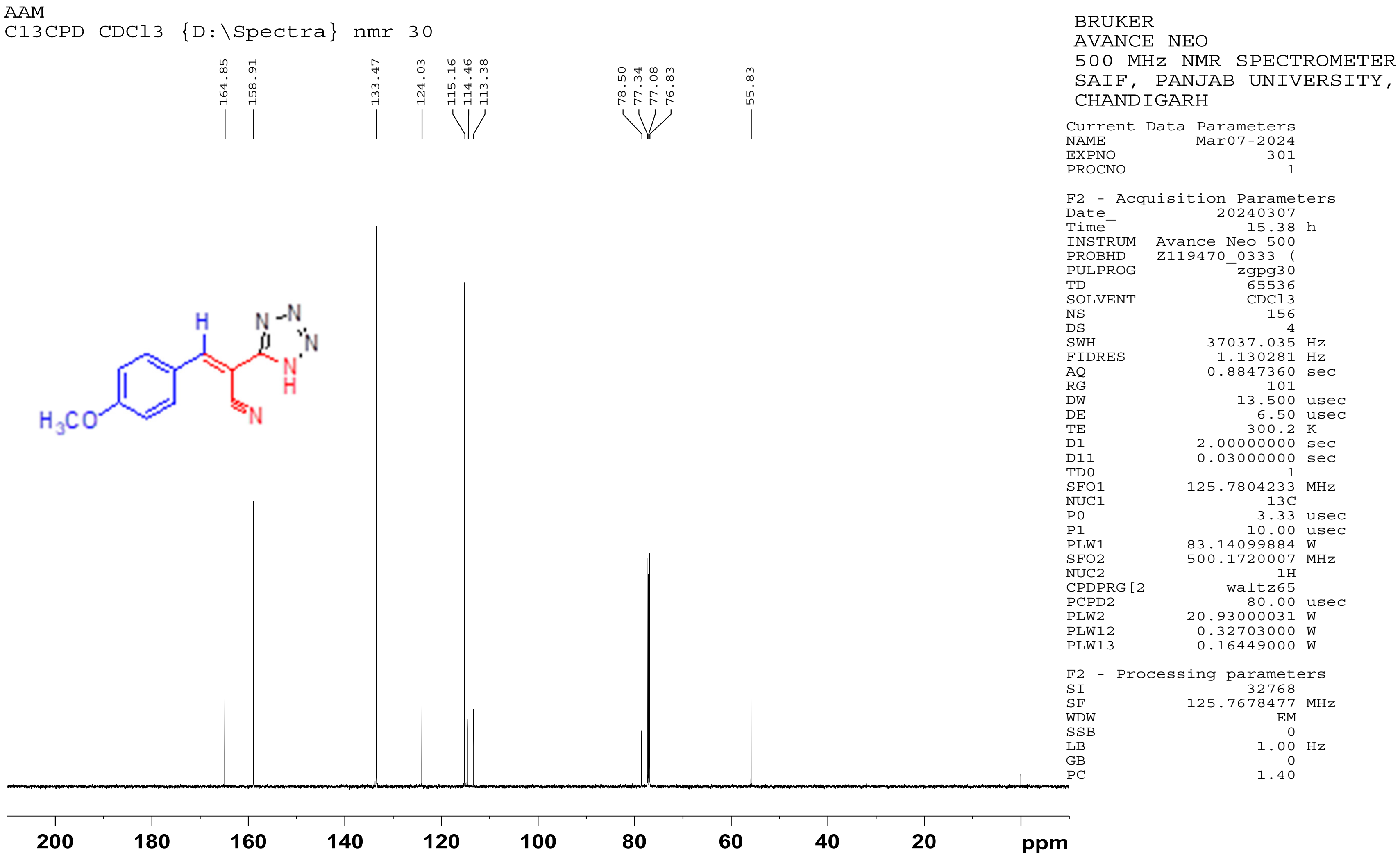

Figure S34: ^{13}C-NMR spectra of (Z)-3-(4-methoxyphenyl)-2-(1H-tetrazol-5-yl)acrylonitrile **(4h)**

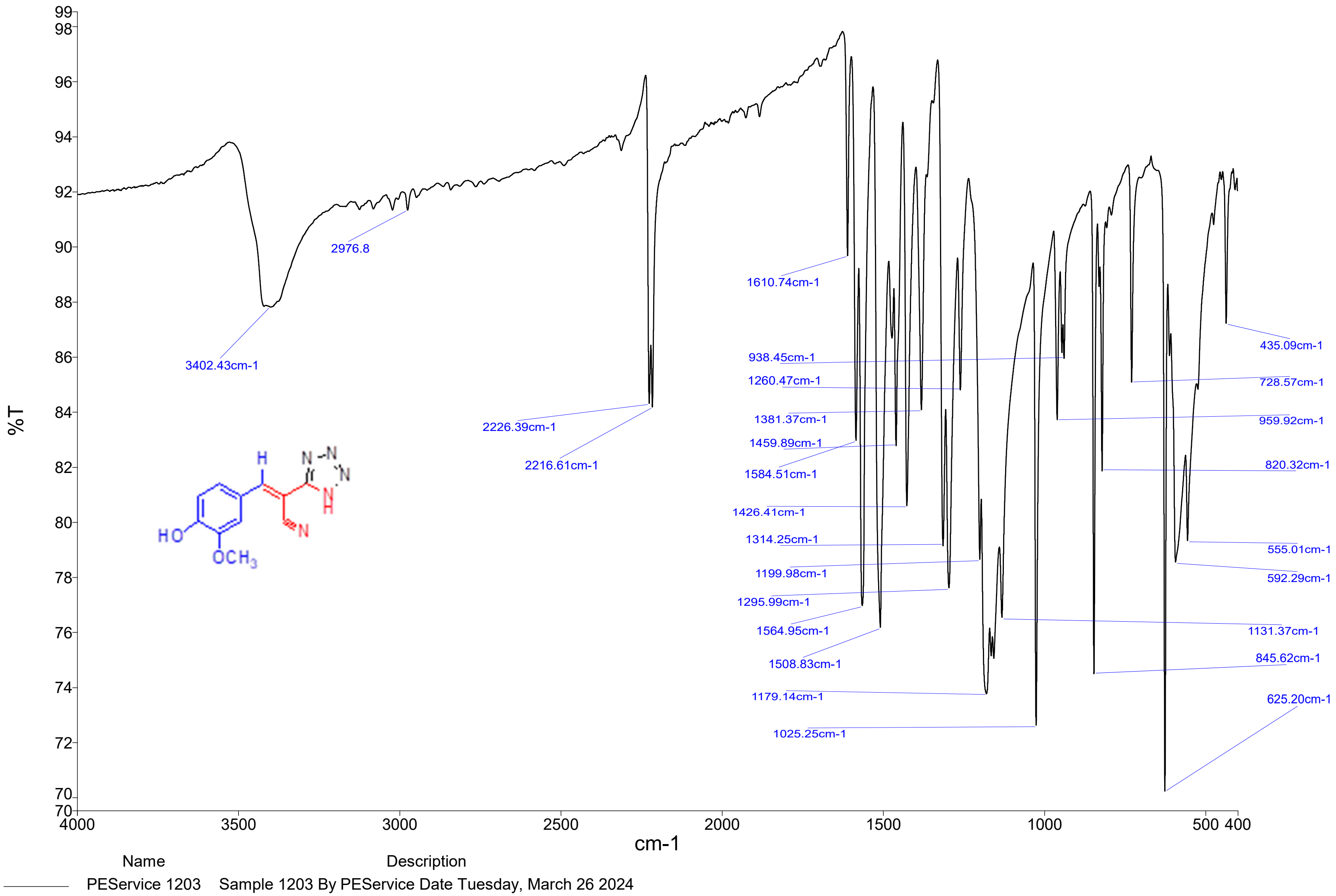

Figure S35: FT-IR spectra of (Z)-3-(4-hydroxy-3-methoxyphenyl)-2-(1H-tetrazol-5-yl)acrylonitrile **(4i)**

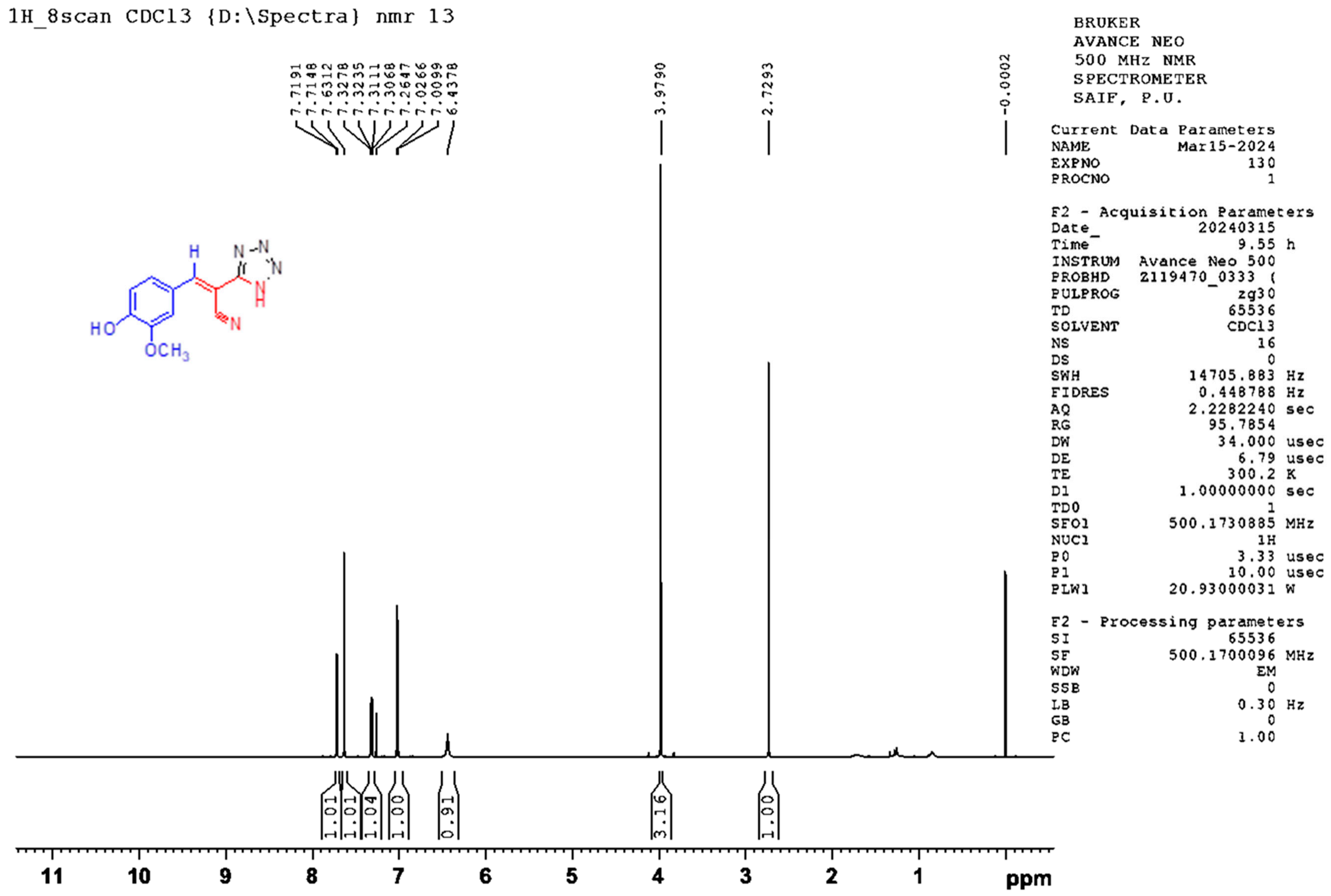

Figure S36: ^{1}H-NMR spectra of (Z)-3-(4-hydroxy-3-methoxyphenyl)-2-(1H-tetrazol-5-yl)acrylonitrile **(4i)**

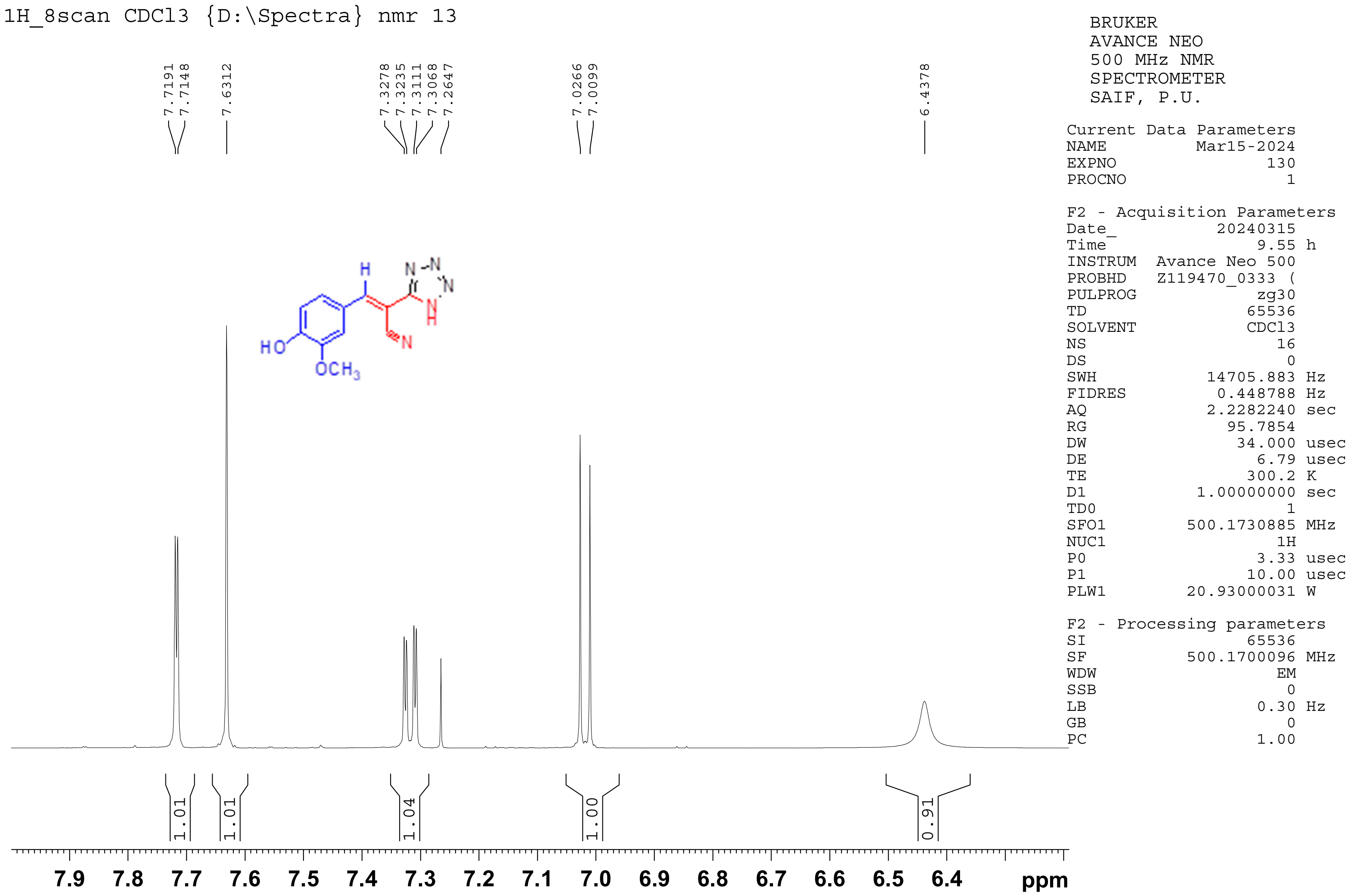

Figure S37: ^{1}H-NMR expanded spectra of (Z)-3-(4-hydroxy-3-methoxyphenyl)-2-(1H-tetrazol-5-yl)acrylonitrile **(4i)**

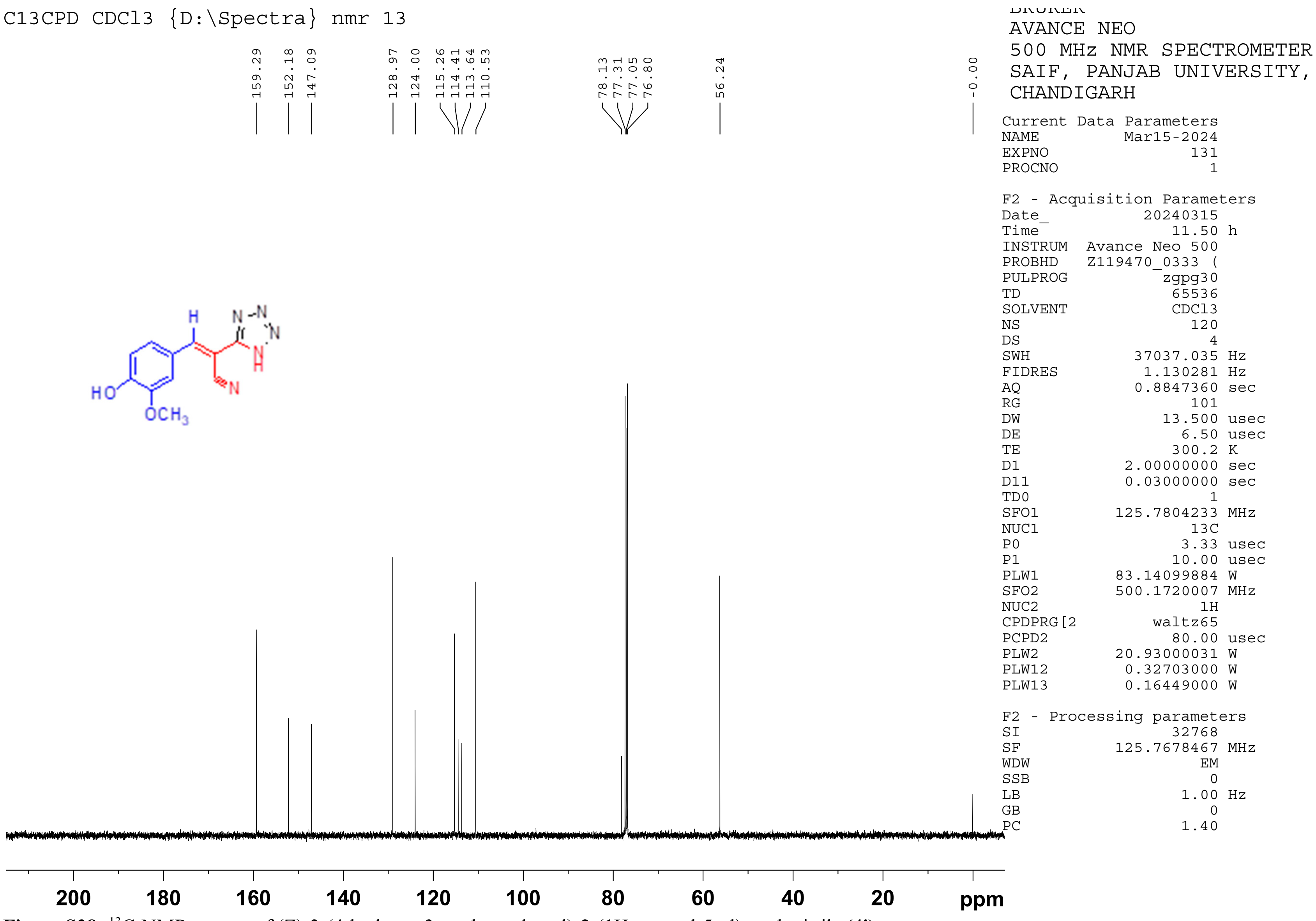

Figure S38: ^{13}C-NMR spectra of (Z)-3-(4-hydroxy-3-methoxyphenyl)-2-(1H-tetrazol-5-yl)acrylonitrile **(4i)**

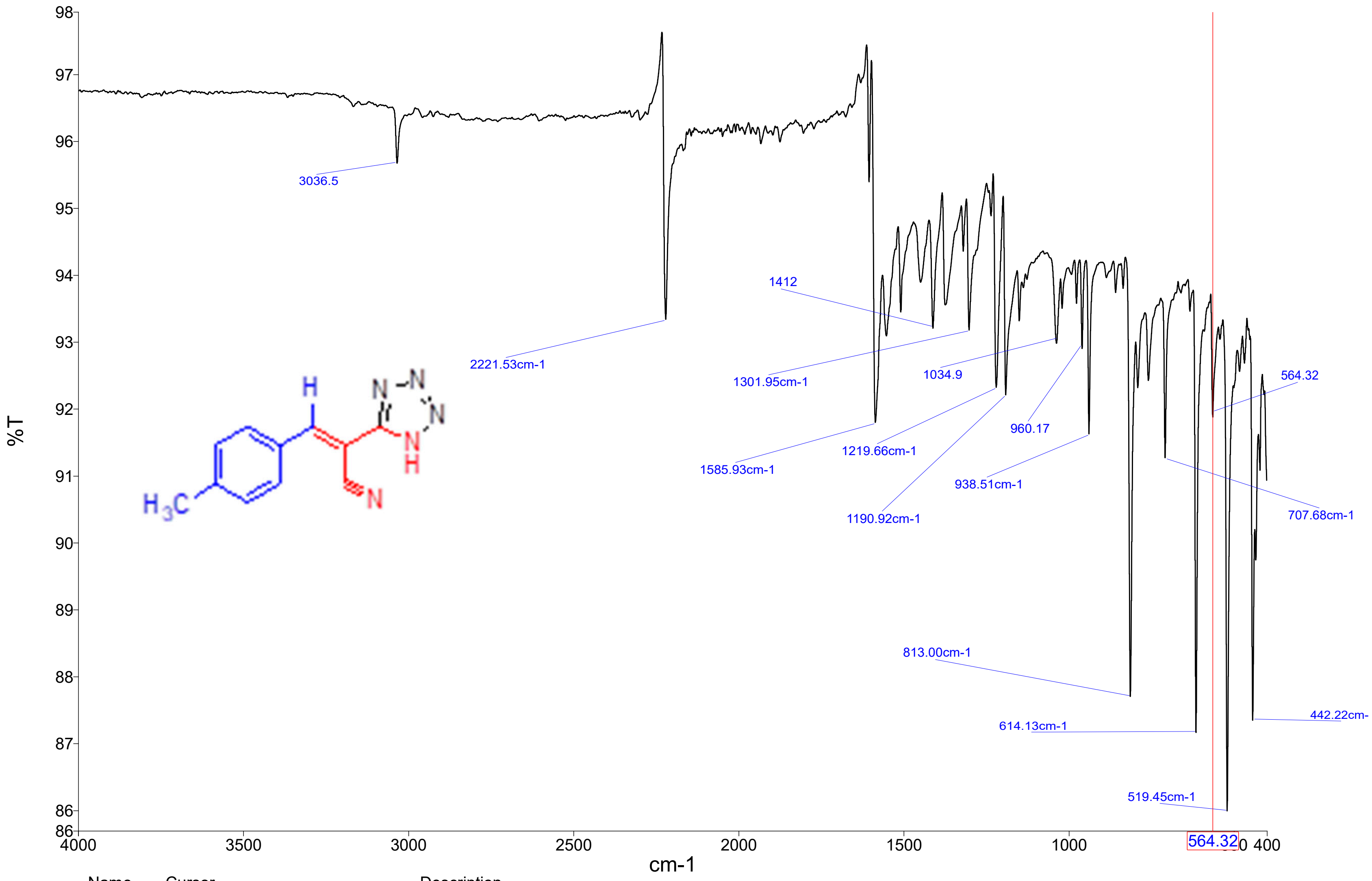

Name	Cursor	Description
4-Me	91.955 %T	Sample 1202 By PEService Date Tuesday, March 26 2024

Figure S39: FT-IR spectra of (Z)-3-(4-methoxyphenyl)-2-(1H-tetrazol-5-yl)acrylonitrile **(4j)**

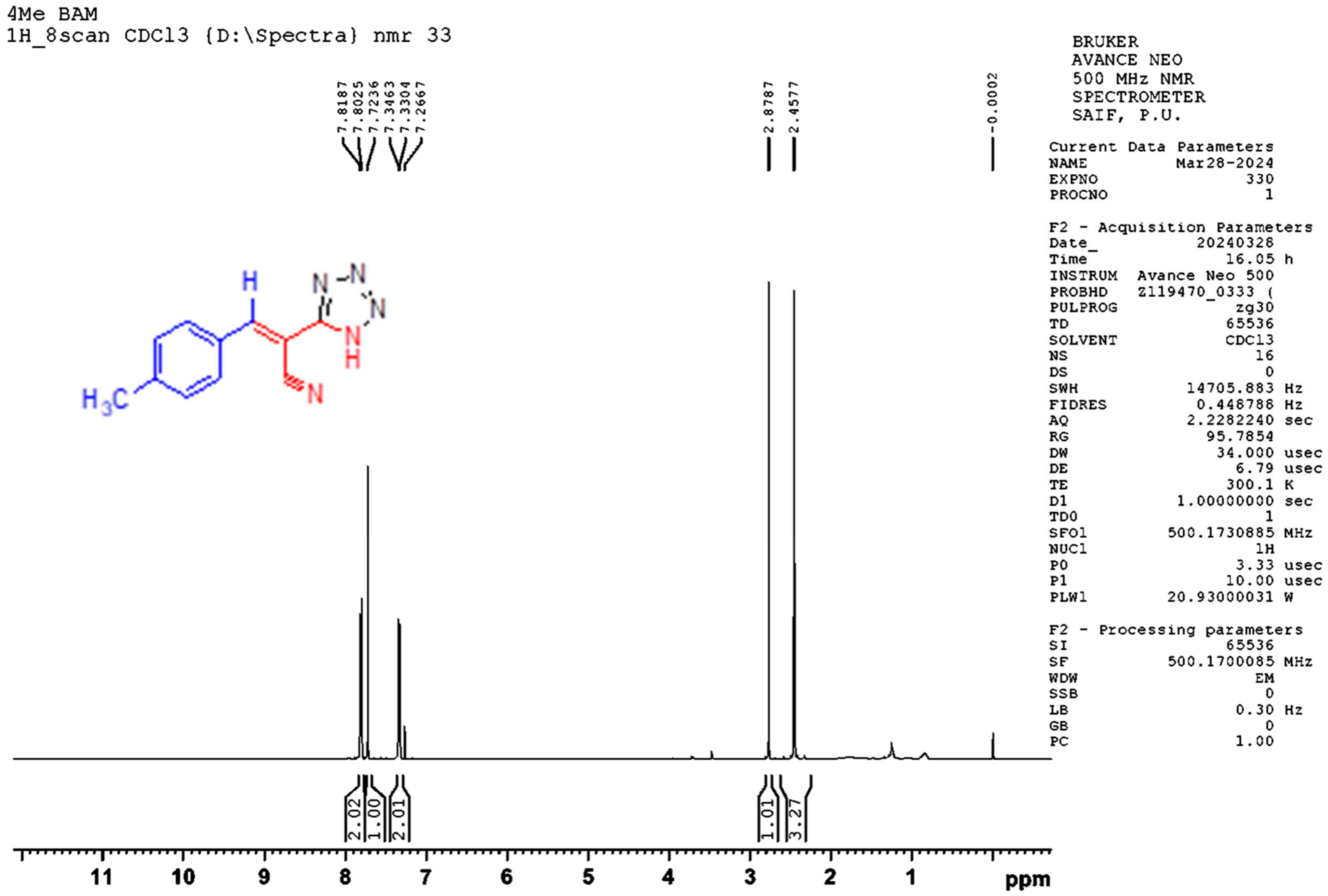

Figure S40: ^{1}H-NMR spectra of (Z)-3-(4-methoxyphenyl)-2-(1H-tetrazol-5-yl)acrylonitrile **(4j)**

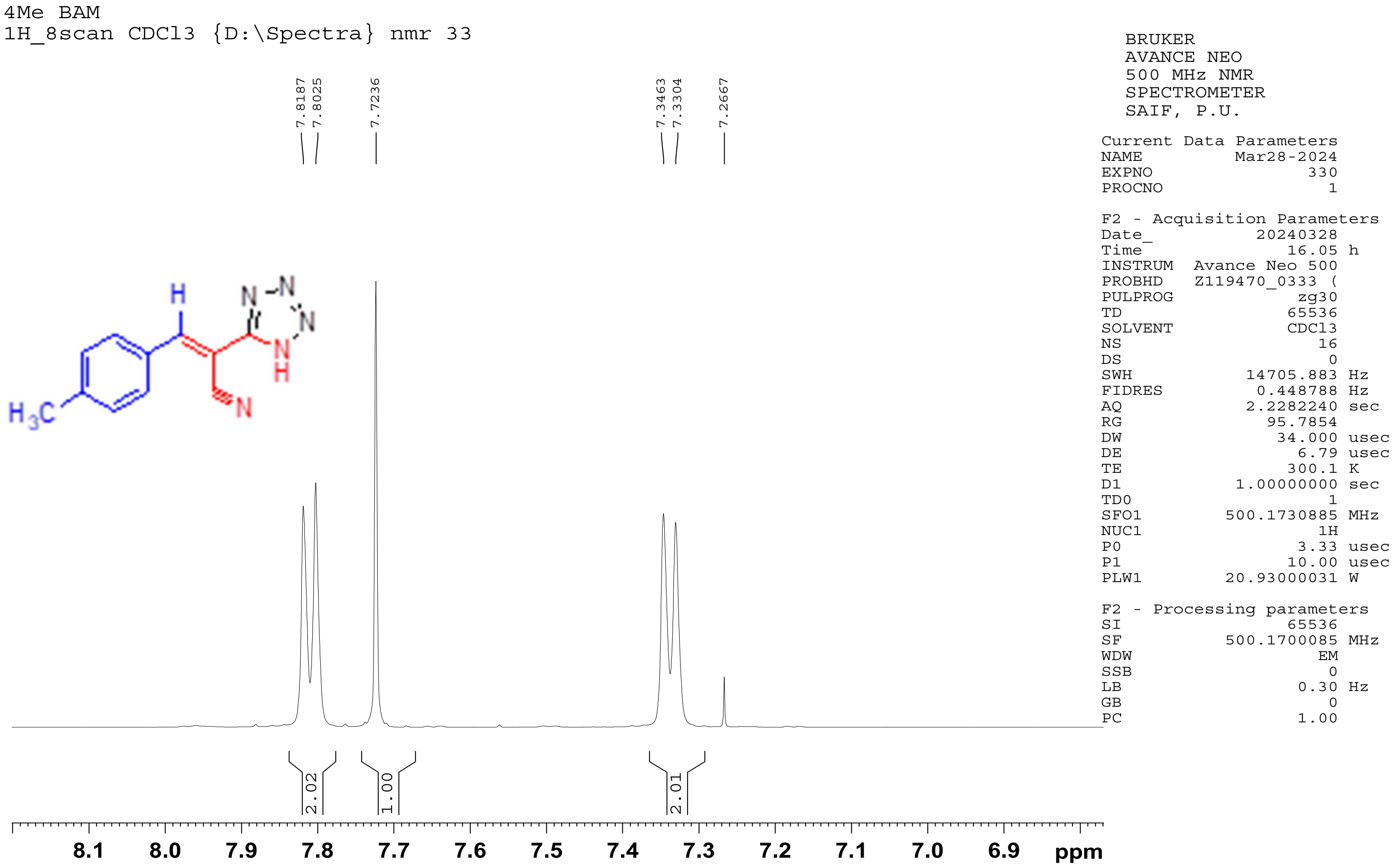

Figure S41: ^{1}H-NMR expanded spectra of (Z)-3-(4-methoxyphenyl)-2-(1H-tetrazol-5-yl)acrylonitrile **(4j)**

4Me BAM
C13CPD CDCl3 {D:\Spectra} nmr 33

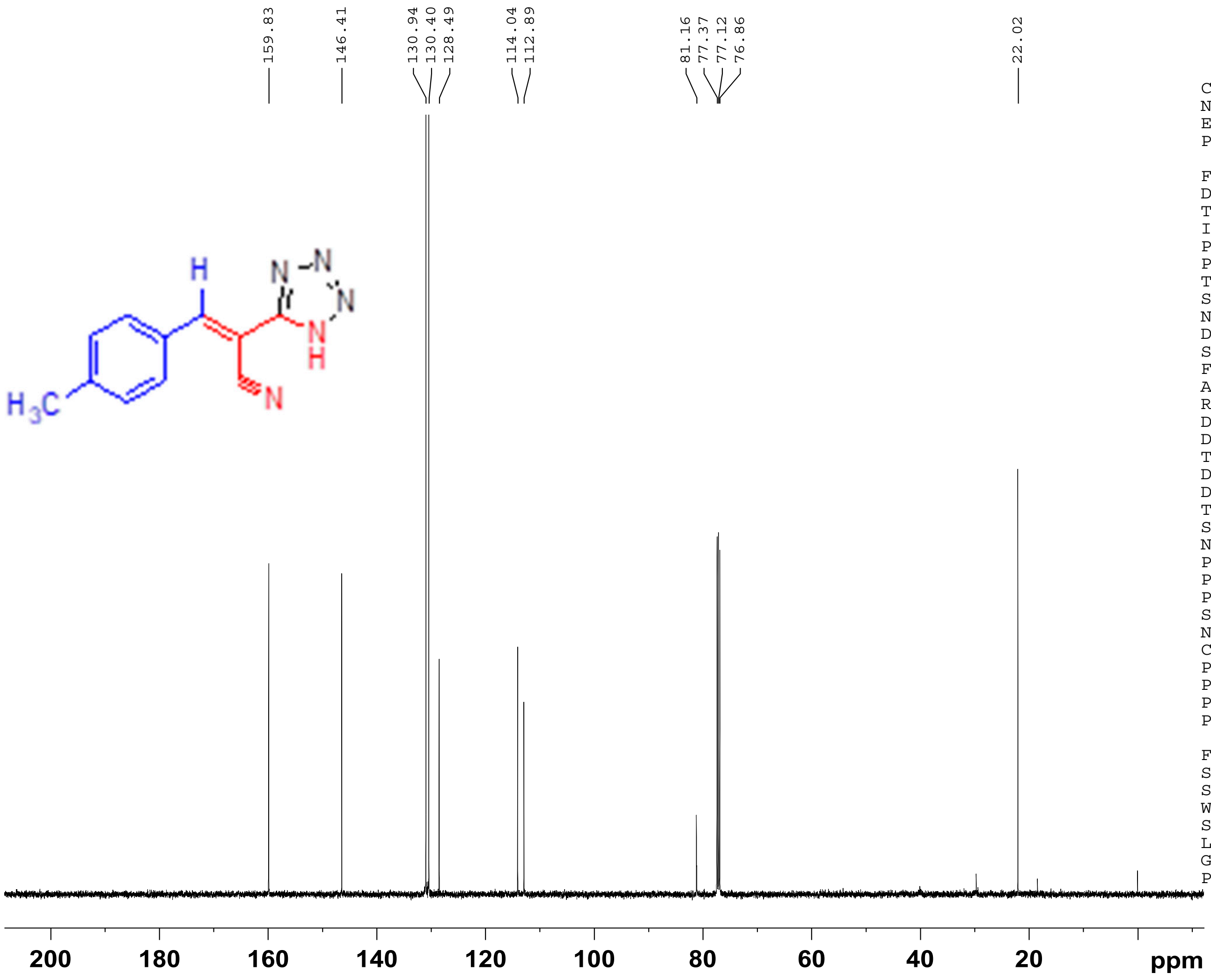

BRUKER
AVANCE NEO
500 MHz NMR SPECTROMETER
SAIF, PANJAB UNIVERSITY,
CHANDIGARH

Current Data Parameters
NAME Mar28-2024
EXPNO 331
PROCNO 1

F2 - Acquisition Parameters
Date_ 20240329
Time 6.51 h
INSTRUM Avance Neo 500
PROBHD Z119470_0333 (
PULPROG zgpg30
TD 65536
SOLVENT CDCl3
NS 256
DS 4
SWH 37037.035 Hz
FIDRES 1.130281 Hz
AQ 0.8847360 sec
RG 101
DW 13.500 usec
DE 6.50 usec
TE 300.2 K
D1 2.00000000 sec
D11 0.03000000 sec
TD0 1
SFO1 125.7804233 MHz
NUC1 13C
P0 3.33 usec
P1 10.00 usec
PLW1 83.14099884 W
SFO2 500.1720007 MHz
NUC2 1H
CPDPRG[2 waltz65
PCPD2 80.00 usec
PLW2 20.93000031 W
PLW12 0.32703000 W
PLW13 0.16449000 W

F2 - Processing parameters
SI 32768
SF 125.7678465 MHz
WDW EM
SSB 0
LB 1.00 Hz
GB 0
PC 1.40

Figure S42: ^{13}C-NMR spectra of (Z)-3-(4-methoxyphenyl)-2-(1H-tetrazol-5-yl)acrylonitrile **(4j)**

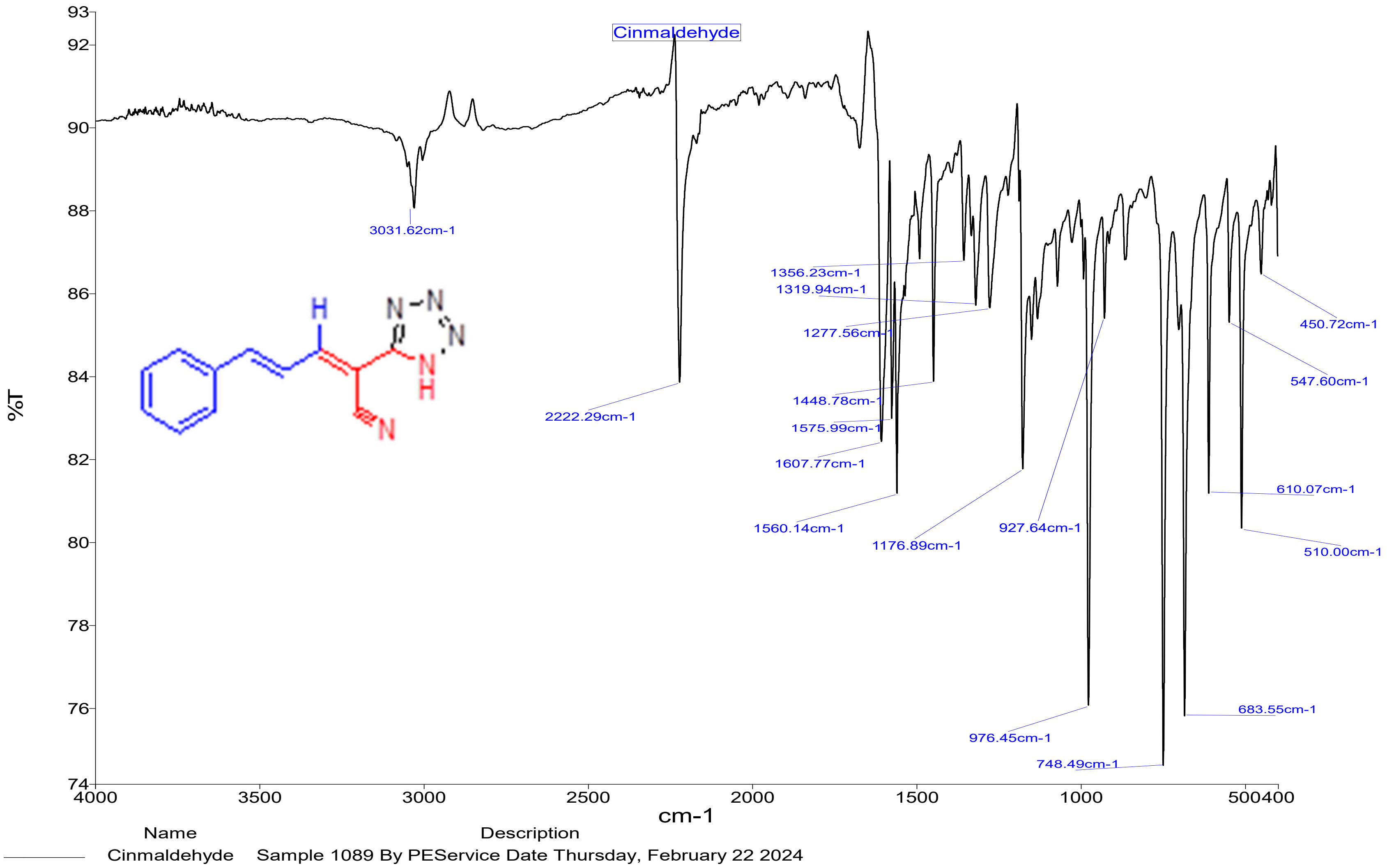

Figure S43: FT-IR spectra of (2Z,4E)-5-phenyl-2-(1H-tetrazol-5-yl)penta-2,4-dienenitrile **(4k)**

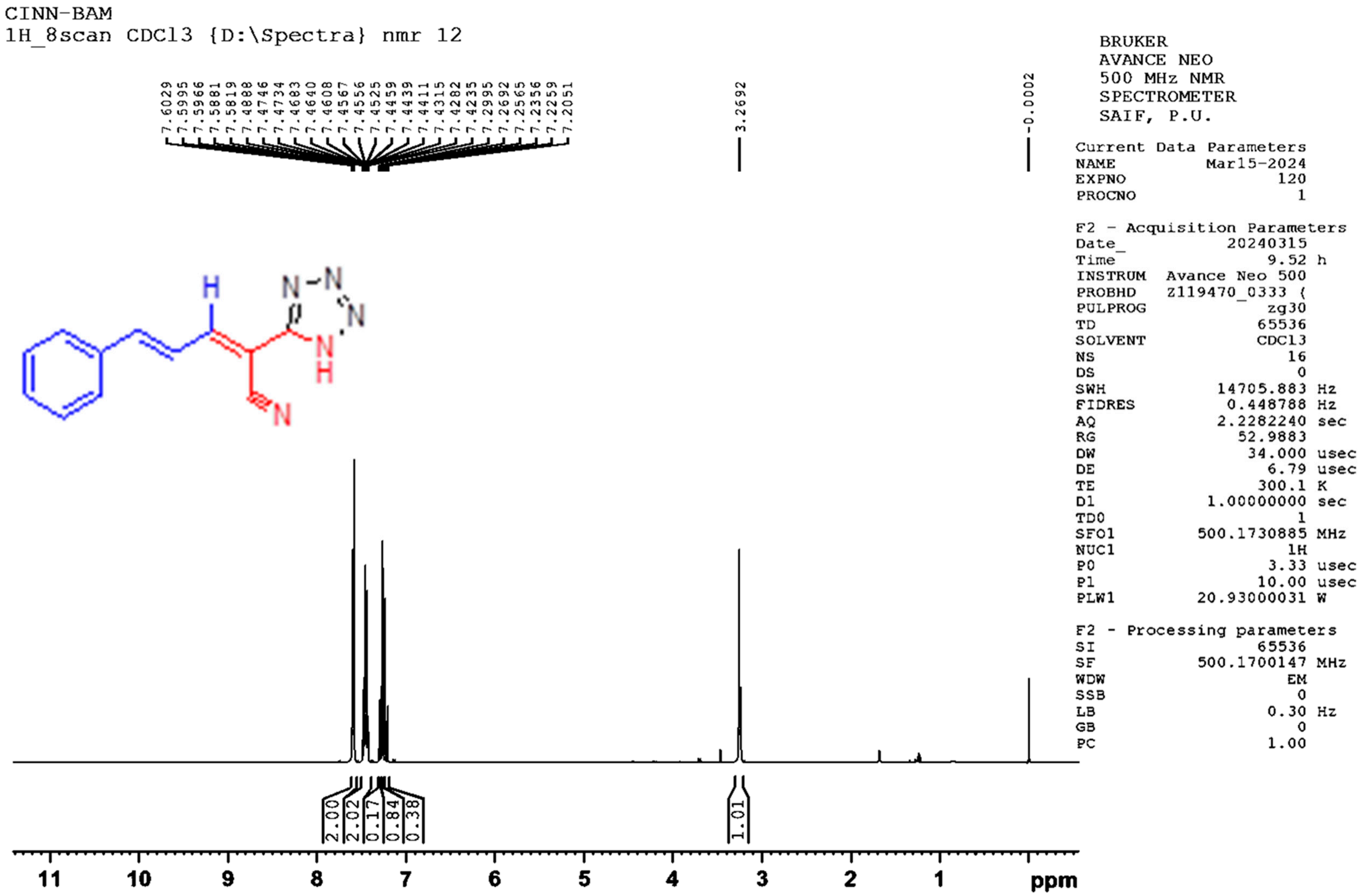

Figure S44: ^{1}H-NMR spectra of (2Z,4E)-5-phenyl-2-(1H-tetrazol-5-yl)penta-2,4-dienenitrile **(4k)**

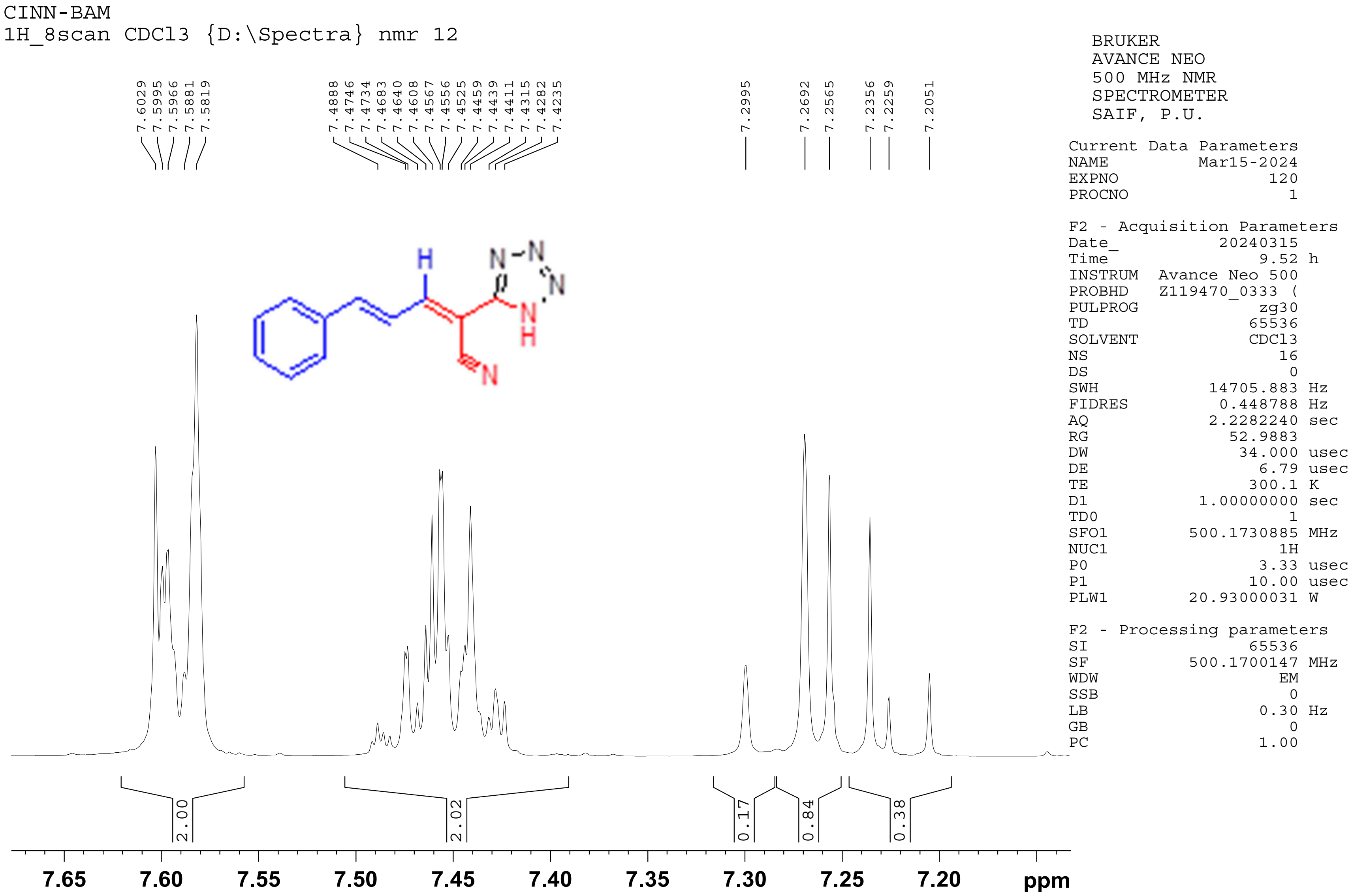

Figure S45: ^{1}H-NMR expanded spectra of (2Z,4E)-5-phenyl-2-(1H-tetrazol-5-yl)penta-2,4-dienenitrile **(4k)**

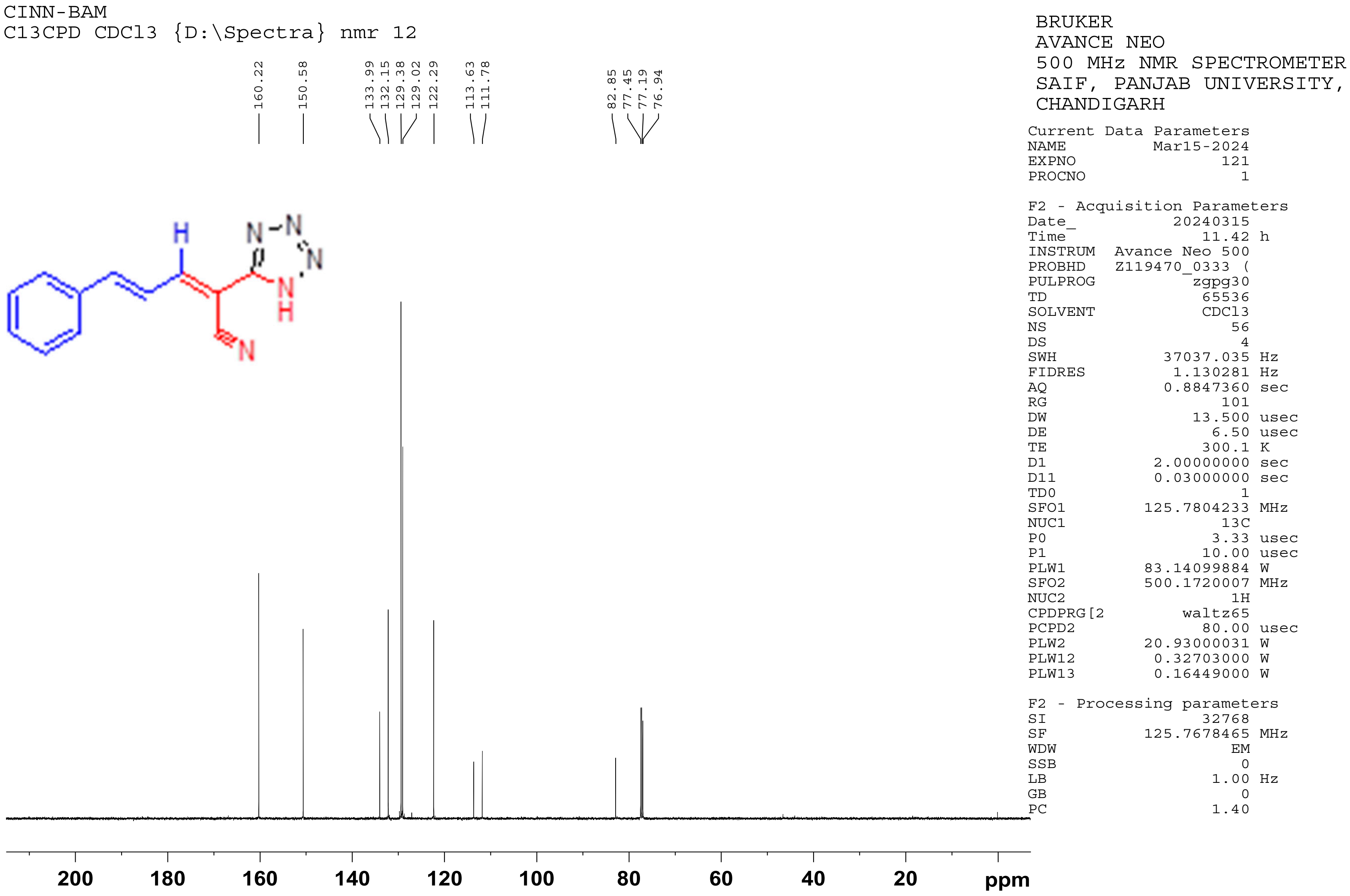

Figure S46: ^{13}C-NMR spectra of (2Z,4E)-5-phenyl-2-(1H-tetrazol-5-yl)penta-2,4-dienenitrile **(4k)**

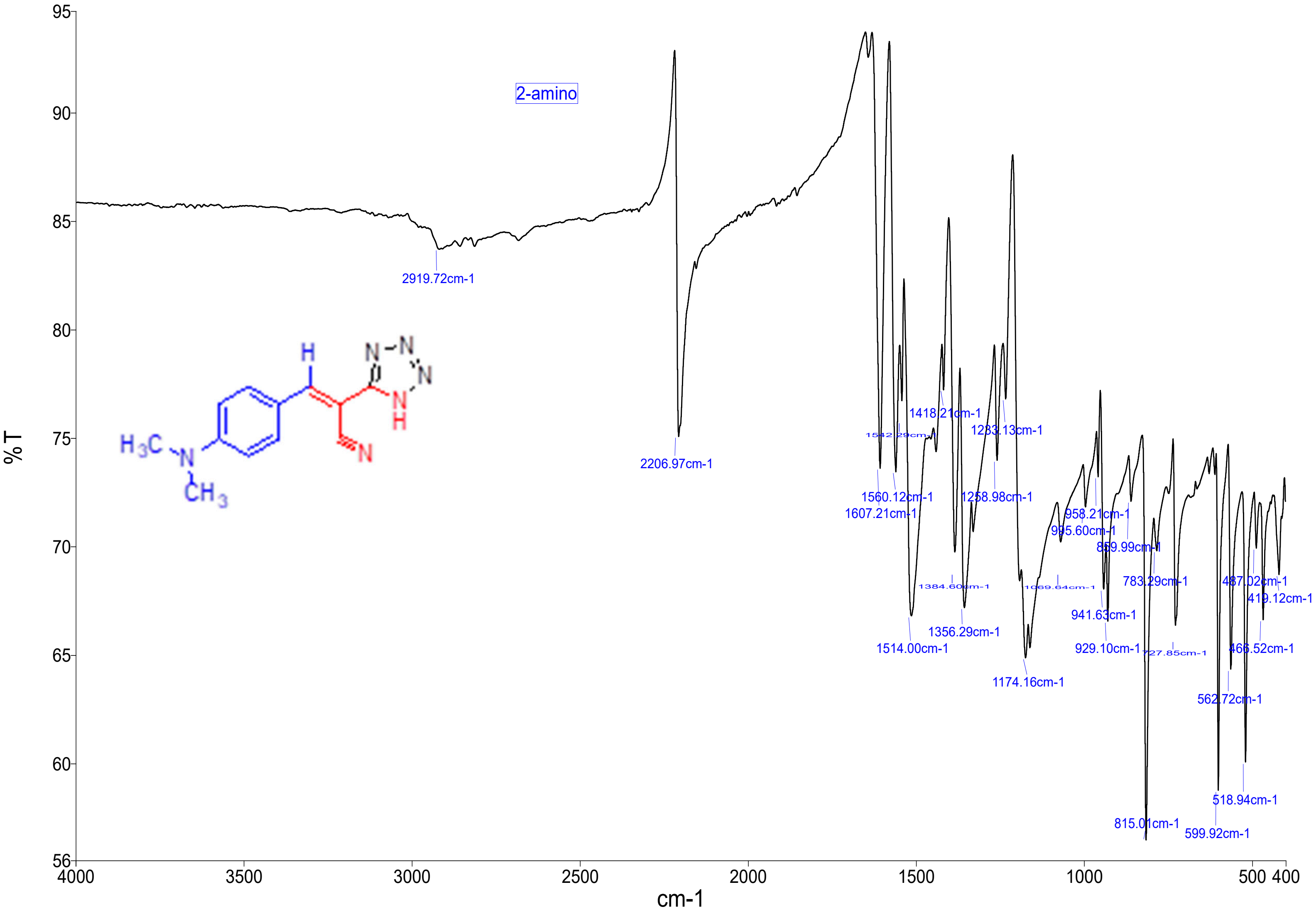

Figure S47: FT-IR spectra of (Z)-3-(4-(dimethylamino)phenyl)-2-(1H-tetrazol-5-yl)acrylonitrile **(4l)**

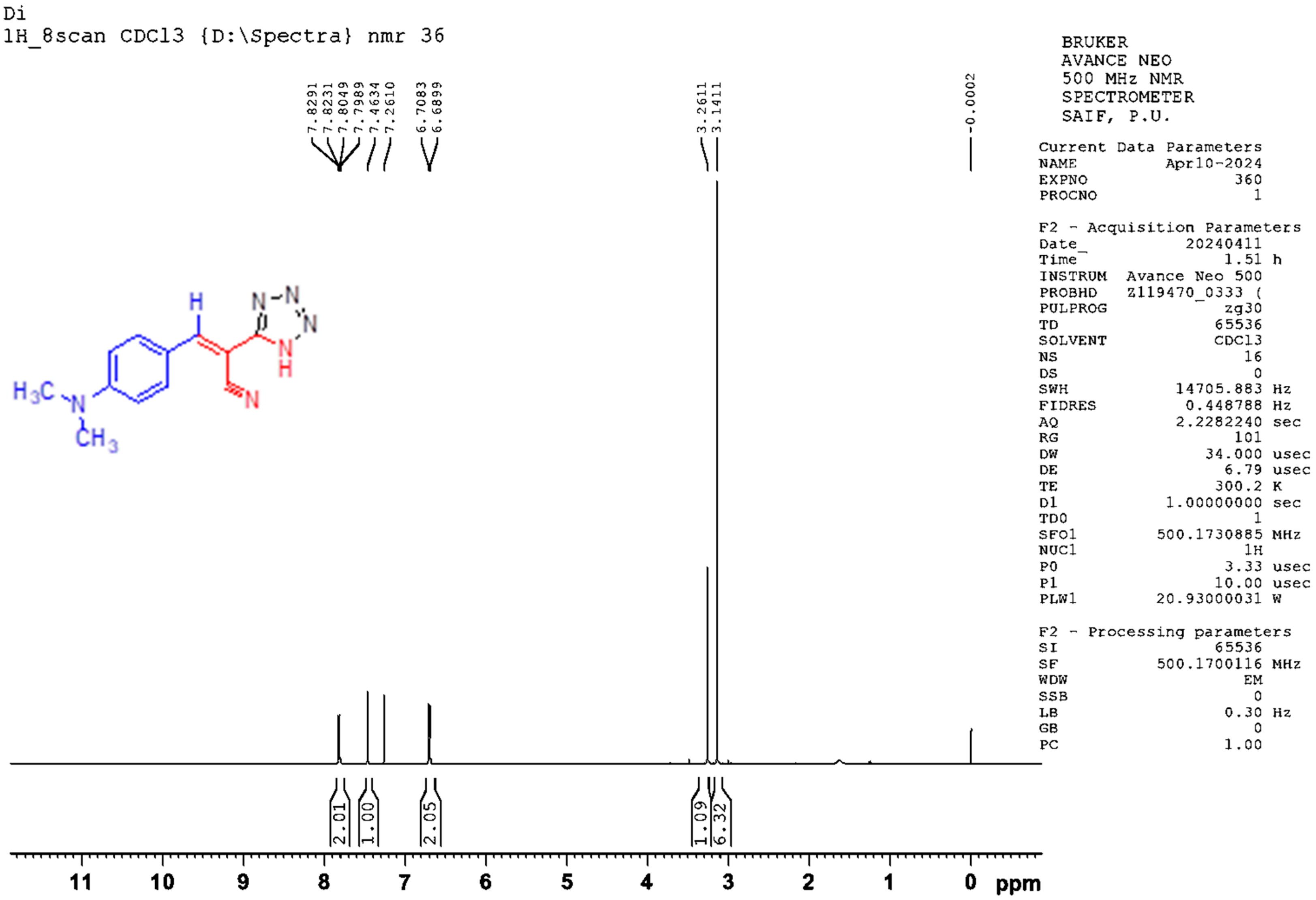

Figure S48: ^{1}H-NMR spectra of (Z)-3-(4-(dimethylamino)phenyl)-2-(1H-tetrazol-5-yl)acrylonitrile **(4l)**

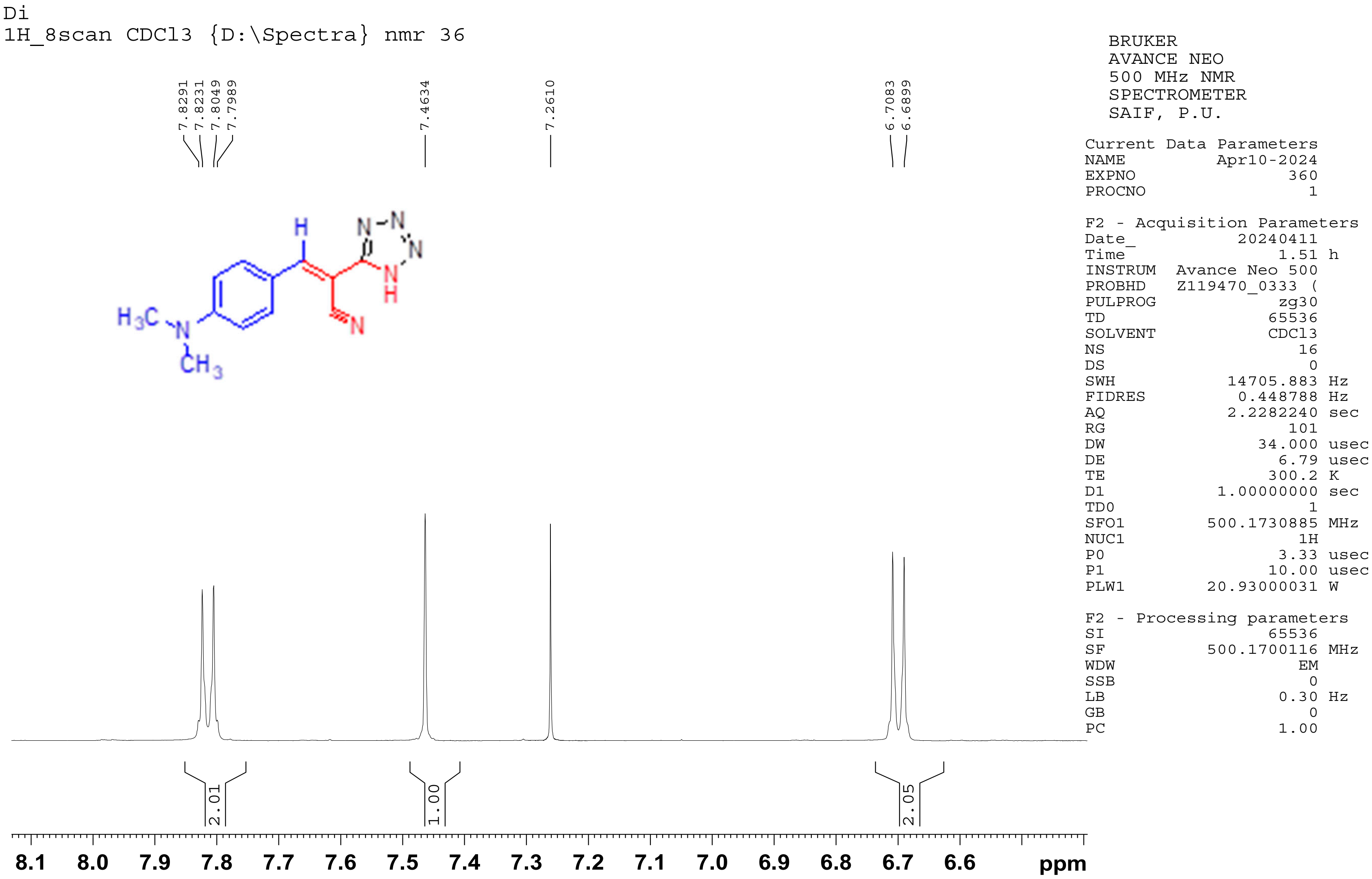

Figure S49: ^{1}H-NMR expanded spectra of (Z)-3-(4-(dimethylamino)phenyl)-2-(1H-tetrazol-5-yl)acrylonitrile **(4l)**

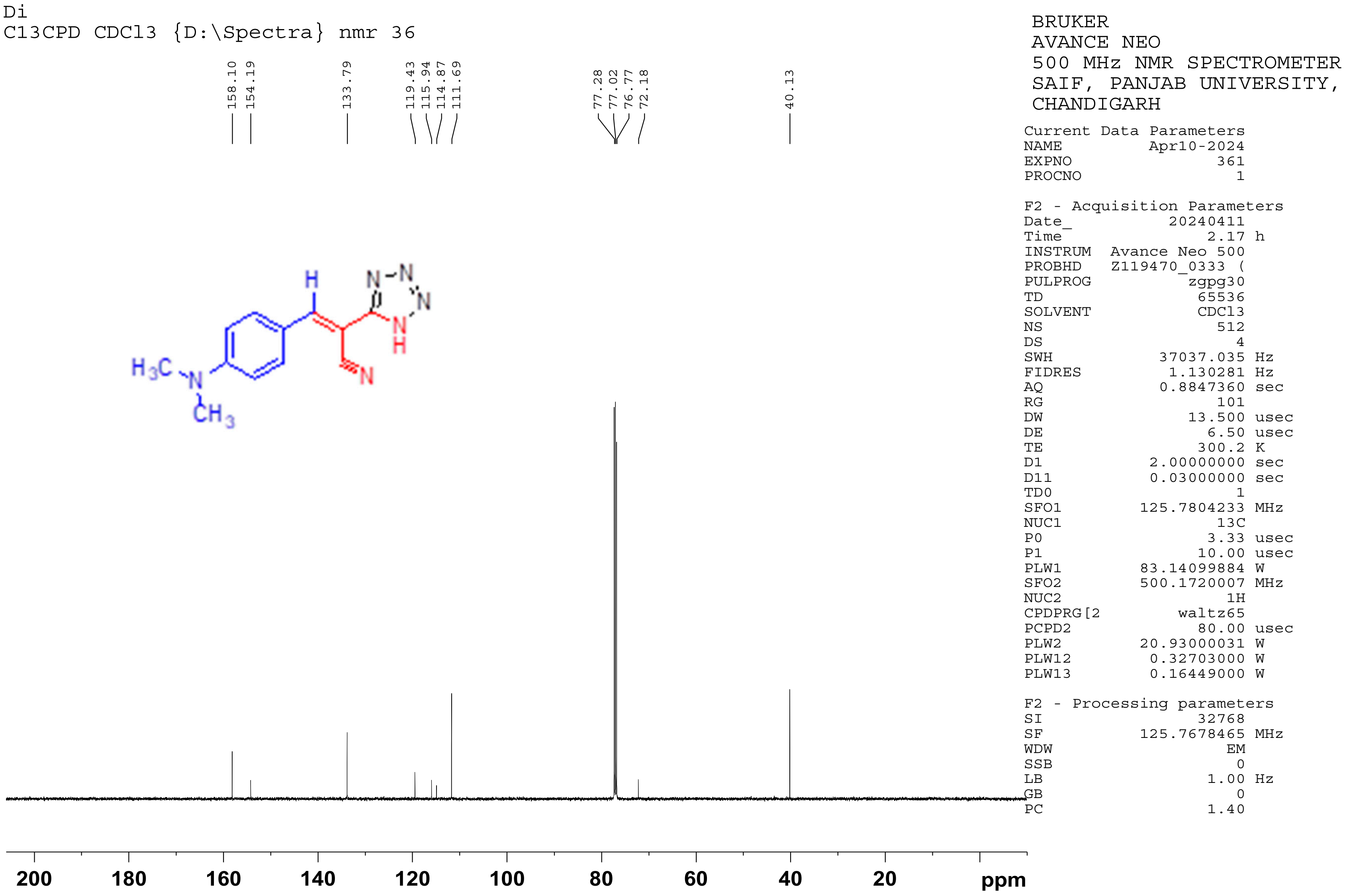

Figure S50: ^{13}C-NMR spectra of (Z)-3-(4-(dimethylamino)phenyl)-2-(1H-tetrazol-5-yl)acrylonitrile **(4l)**

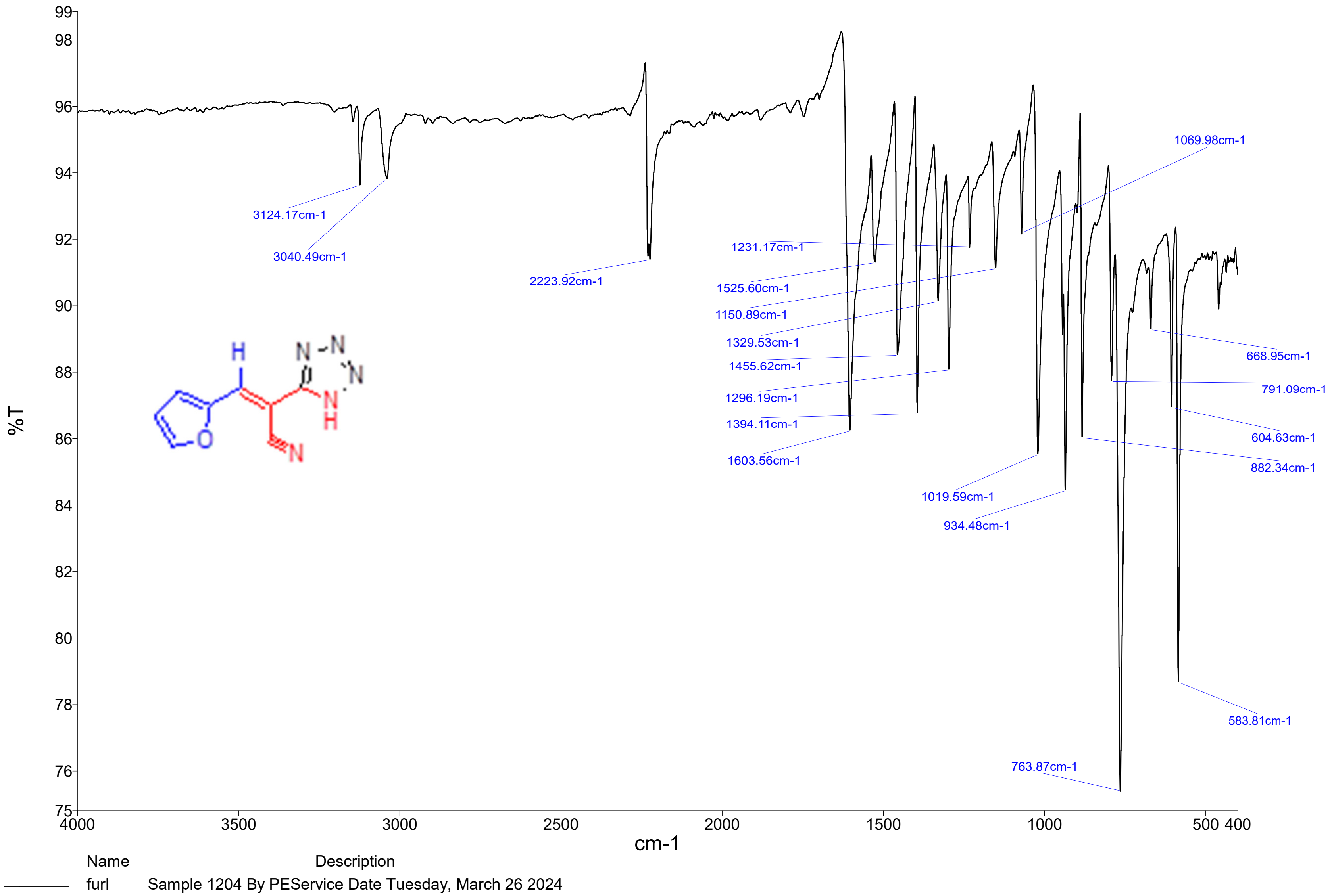

Figure S51: FT-IR spectra of (Z)-3-(furan-2-yl)-2-(1H-tetrazol-5-yl)acrylonitrile **(4m)**

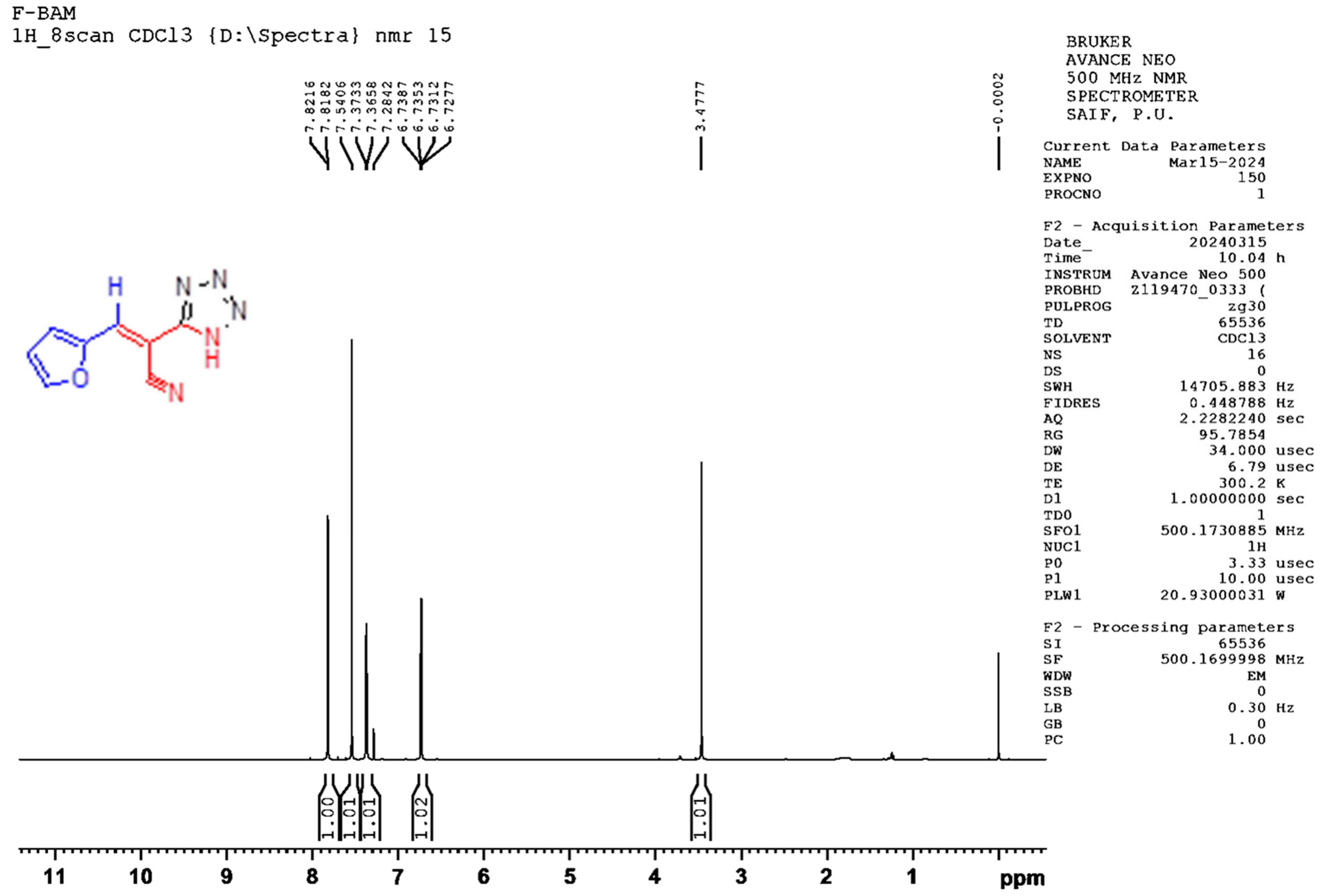

Figure S52: ^{1}H-NMR spectra of (Z)-3-(furan-2-yl)-2-(1H-tetrazol-5-yl)acrylonitrile **(4m)**

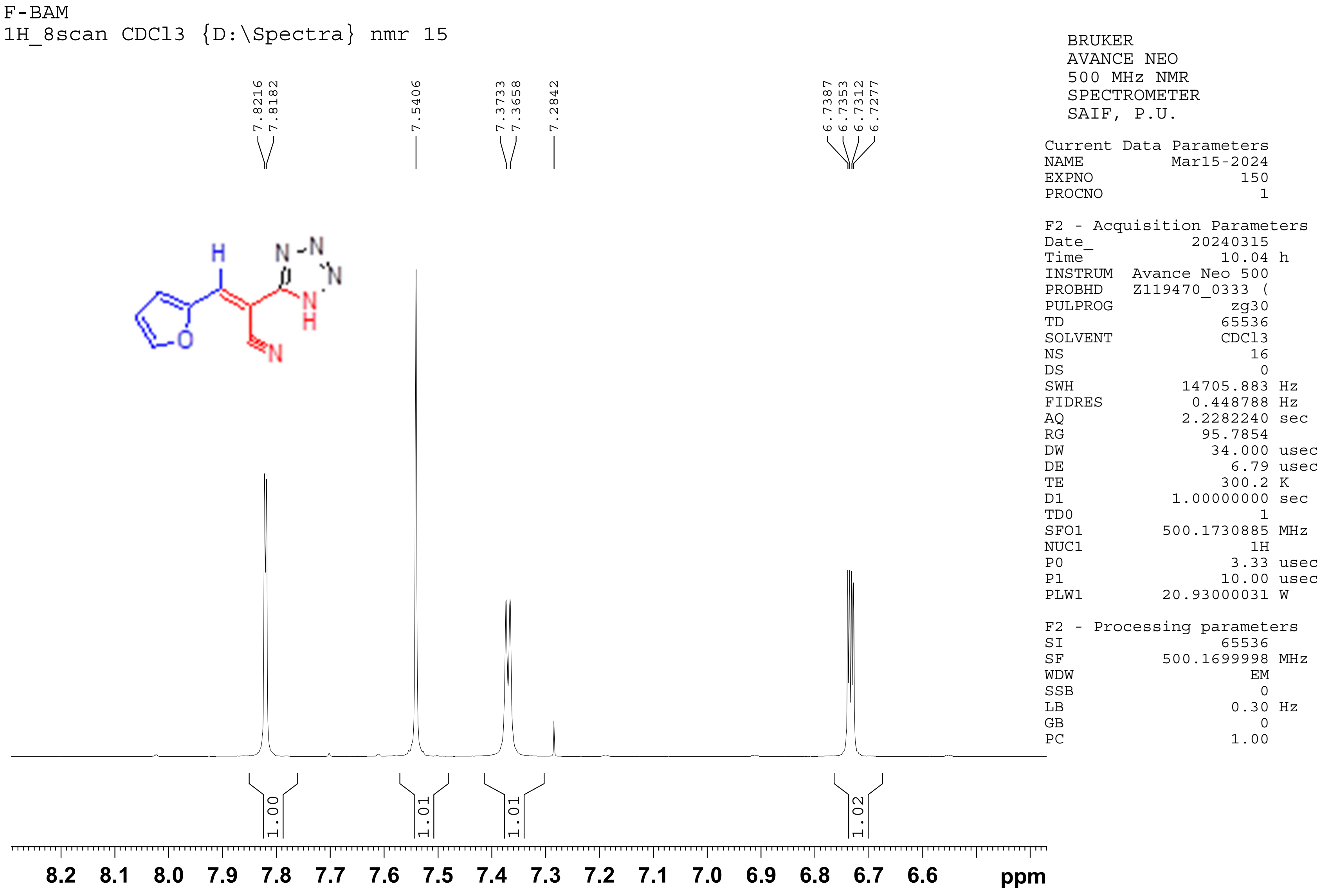

Figure S53: ^{1}H-NMR expanded spectra of (Z)-3-(furan-2-yl)-2-(1H-tetrazol-5-yl)acrylonitrile **(4m)**

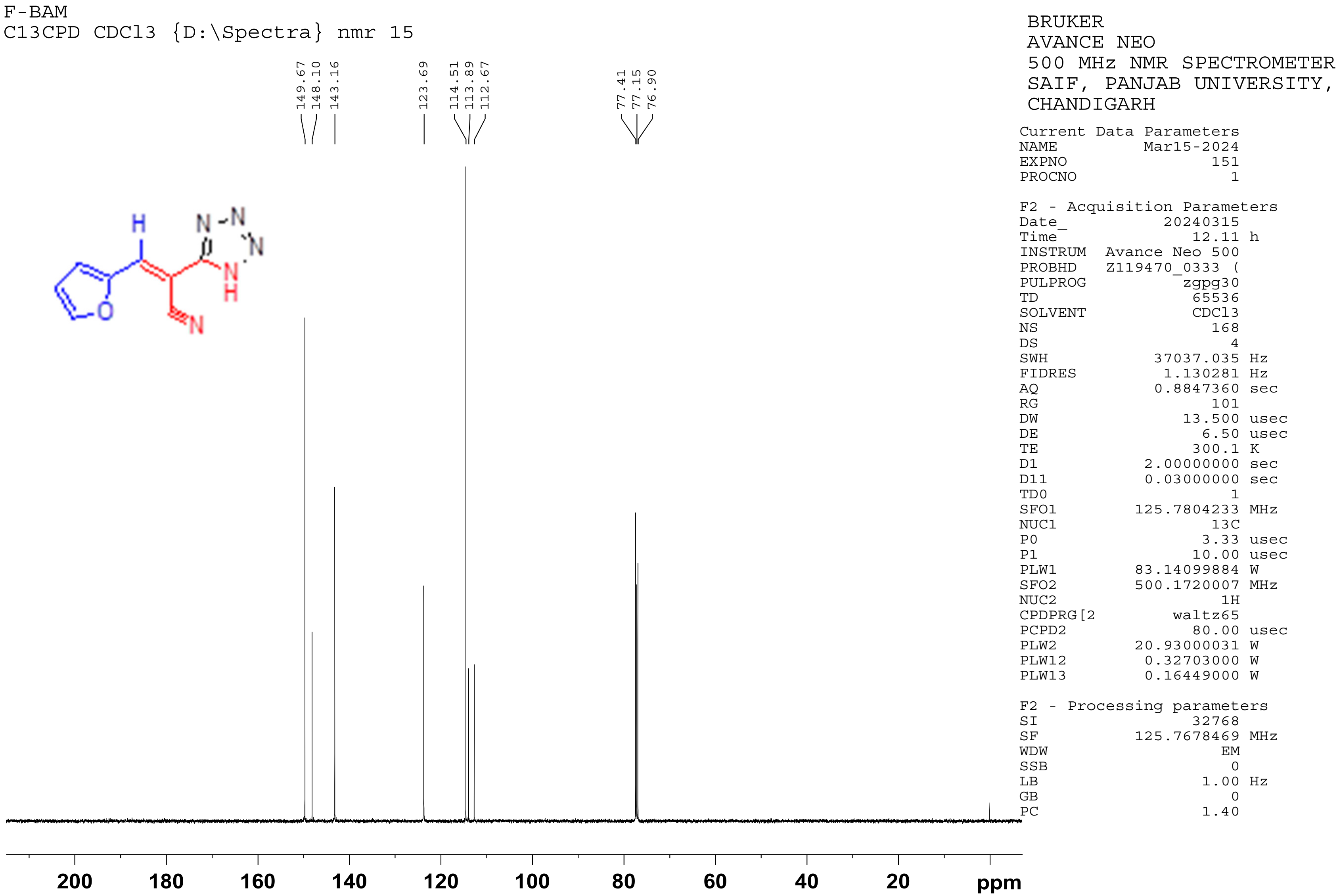

Figure S54: ^{13}C-NMR spectra of (Z)-3-(furan-2-yl)-2-(1H-tetrazol-5-yl)acrylonitrile **(4m)**

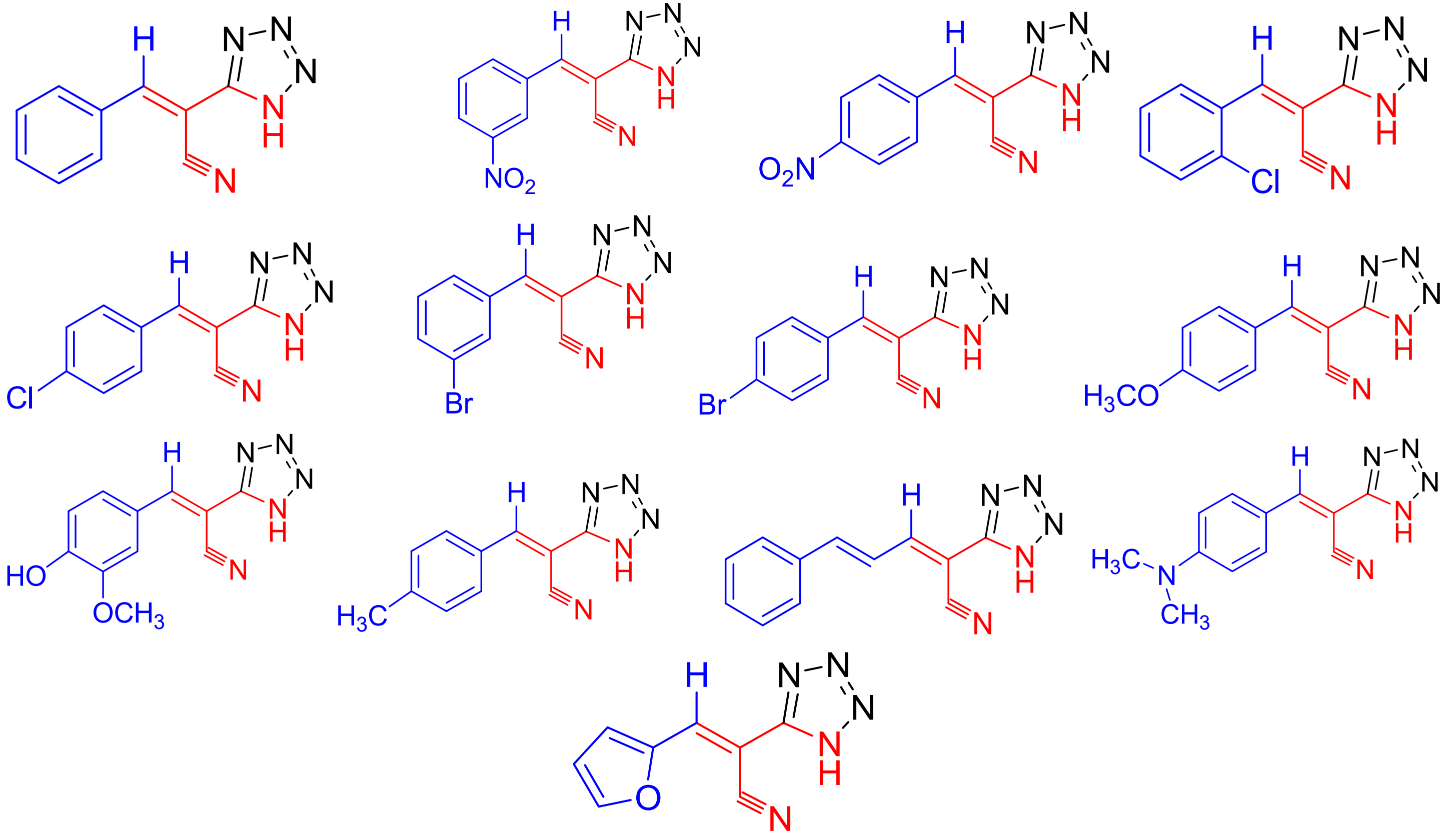

Figure S55: Structures *of (Z)-3-phenyl-2-(1H-tetrazol-5-yl)acrylonitrile* ***5a-m***.
